# Injectable BMSC‐Based Extracellular Matrix‐Mimicking Microtissue for Myocardial Infarction Repair

**DOI:** 10.1002/advs.202500299

**Published:** 2025-09-15

**Authors:** Lina Yao, Huailong An, Cong Fan, Qingsu Lan, Hongyu Zhong, Yun Zhang, Libo Zhou, Panpan Hao

**Affiliations:** ^1^ State Key Laboratory for Innovation and Transformation of Luobing Theory Key Laboratory of Cardiovascular Remodeling and Function Research of MOE NHC, CAMS and Shandong Province Department of Cardiology Qilu Hospital of Shandong University Cheeloo College of Medicine Shandong University Jinan Shandong 250012 P. R. China; ^2^ Department of Medicinal Chemistry Shandong Key Laboratory of Druggability Optimization and Evaluation for Lead Compounds School of Pharmaceutical Sciences Cheeloo College of Medicine Shandong University Jinan Shandong 250012 P. R. China

**Keywords:** biomimetic microstructure, bone marrow‐derived mesenchymal stem cells, induced directional differentiation, long‐term retention, myocardial infarction

## Abstract

After myocardial infarction, the myocardial microenvironment is altered by cardiomyocyte loss, inflammation, and extracellular matrix degradation, creating a hostile environment that severely limits bone marrow‐derived mesenchymal stem cell (BMSC) survival, migration, and differentiation. The BMSCs may differentiate into cardiomyocyte‐like cells in vitro. However, this potential remains significantly limited in vivo because of the lack of supportive signals and conducive microenvironments. To overcome these challenges, this study presents a novel injectable biomimetic microtissue system containing a dual biomimetic extracellular matrix scaffold consisting of Janus Basic Nanotubes, laminin, stromal‐derived factor‐1 alpha, and vascular endothelial growth factor (JLSV) for BMSC delivery (JLSV–BMSC microtissue) designed to mimic the natural myocardial microenvironment. In vitro experiments showed that the microtissue enhanced BMSC survival, proliferation, migration, anti‐apoptotic capacity, and paracrine signaling under oxygen–glucose deprivation conditions. In vivo studies have shown that the microtissue significantly improves BMSC retention at the infarct site, directs their differentiation into cardiomyocytes and endothelial cells, reduces myocardial fibrosis and apoptosis, and promotes angiogenesis, contributing to improved cardiac remodeling and functional recovery. These results suggest that the JLSV–BMSC microtissue is a promising therapeutic strategy for myocardial infarction that addresses the critical challenges of stem cell‐based therapies.

## Graphical abstract

A novel injectable biomimetic extracellular matrix scaffold (JLSV) is designed for enhanced delivery of bone marrow‐derived mesenchymal stem cells (BMSCs). By utilizing janus basic nanotubes, laminin, stromal‐derived factor‐1 alpha, and vascular endothelial growth factor, this innovative JLSV–BMSC microtissue shows promise in myocardial infarction treatment by improving BMSC retention, guiding differentiation, amplifying paracrine signaling, reducing fibrosis, and boosting angiogenesis.

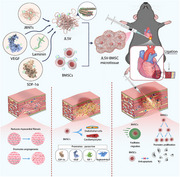

## 1 Introduction

Myocardial infarction (MI) has garnered significant attention owing to its high incidence and mortality^[^ ^1^ ^]^ and often causes heart failure owing to irreversible cardiomyocyte loss and limited regenerative capacity of the adult heart.^[^ ^2^ ^]^ While current therapies, including pharmacological treatments and revascularizing procedures^[^ ^3^ ^]^, alleviate symptoms, restore blood flow, and reduce early mortality, they cannot solve the core problem: cardiomyocyte loss and structural damage to the heart. Once cardiomyocytes are lost, they cannot regenerate naturally, leading to progressive cardiac dysfunction and the onset of heart failure.^[^ ^4^ ^]^ This underscores the urgent need for innovative therapies that can effectively repair and regenerate damaged cardiac tissues, restore cardiac function, and improve long‐term outcomes in patients with MI.

Recently, stem cell‐based therapies have attracted considerable attention as promising solutions for myocardial repair.^[^ ^5^ ^]^ Bone marrow‐derived mesenchymal stem cells (BMSCs) offer several advantages over embryonic stem cells (ESCs) and induced pluripotent stem cells (iPSCs).^[^ ^6^ ^]^ The low immunogenicity of BMSCs reduces the risk of immune rejection. Additionally, the unique immunomodulatory properties of BMSCs promote a supportive microenvironment that enhances tissue repair and regeneration.^[^ ^7^ ^]^ In contrast to ESCs and iPSCs, BMSCs have a substantially lower risk of teratoma formation and arrhythmia.^[^ ^8^ ^]^ In addition, BMSCs stably differentiate into cardiomyocytes and endothelial cells, making them a promising and safe option for repairing damaged cardiac tissue.^[^ ^9^ ^]^

With these properties, BMSCs have a promising potential for treating MI. However, their efficacy is limited by the challenging post‐infarct microenvironment, such as low oxygenation and perfusion, inflammation, and oxidative stress.^[^ ^10^ ^]^ This challenging microenvironment makes it difficult for transplanted BMSCs to survive and differentiate into the cell types required to repair the myocardium at the infarct site.^[^ ^11^ ^]^ These challenges not only undermine the efficacy of current stem cell‐based therapies but also highlight the urgent need for innovative strategies to improve transplanted stem cell retention, survival, and differentiation.^[^ ^12^ ^]^

Several approaches, including stem cell preconditioning,^[^ ^13^ ^]^ genetic modification,^[^ ^14^ ^]^ hydrogels,^[^ ^15^ ^]^ biological patches,^[^ ^16^ ^]^ and biomaterial scaffolds,^[^ ^11^, ^17^ ^]^ have been explored to overcome these challenges. However, these strategies have some limitations. Preconditioning and genetic modification of BMSCs can slightly improve cell survival, but do not provide robust physical support or the optimal microenvironment required for long‐term BMSC embedding and functional integration.^[^ ^9^, ^18^ ^]^ Cardiac patch administration usually requires epicardial fixation by thoracotomy,^[^ ^18^, ^19^ ^]^ an invasive open‐chest procedure that increases the risk of complications and limits clinical applicability.^[^ ^18^, ^20^ ^]^ Conventional hydrogels restrict the movement of BMSCs, impeding their migration into the infarcted area,^[^ ^15a,b^ ^]^ thereby limiting their ability to repair the infarcted myocardium. The integration of biomaterial‐based scaffolds with living cells^[^ ^5^, ^17^ ^]^ and stem cell nanotherapy^[^ ^21^ ^]^ has emerged as an exciting strategy to control cellular functions. However, these stem cell delivery strategies also have several limitations. They cannot adequately mimic the native extracellular matrix (ECM), are not sufficiently bioactive, and cannot adapt well to the dynamic and complex microenvironment of an infarcted heart.^[^ ^22^ ^]^ Therefore, delivery strategies that can mimic the ECM structure and composition to provide a high‐quality microenvironment for BMSCs under the harsh conditions of MI are urgently needed.

**Figure 1:**
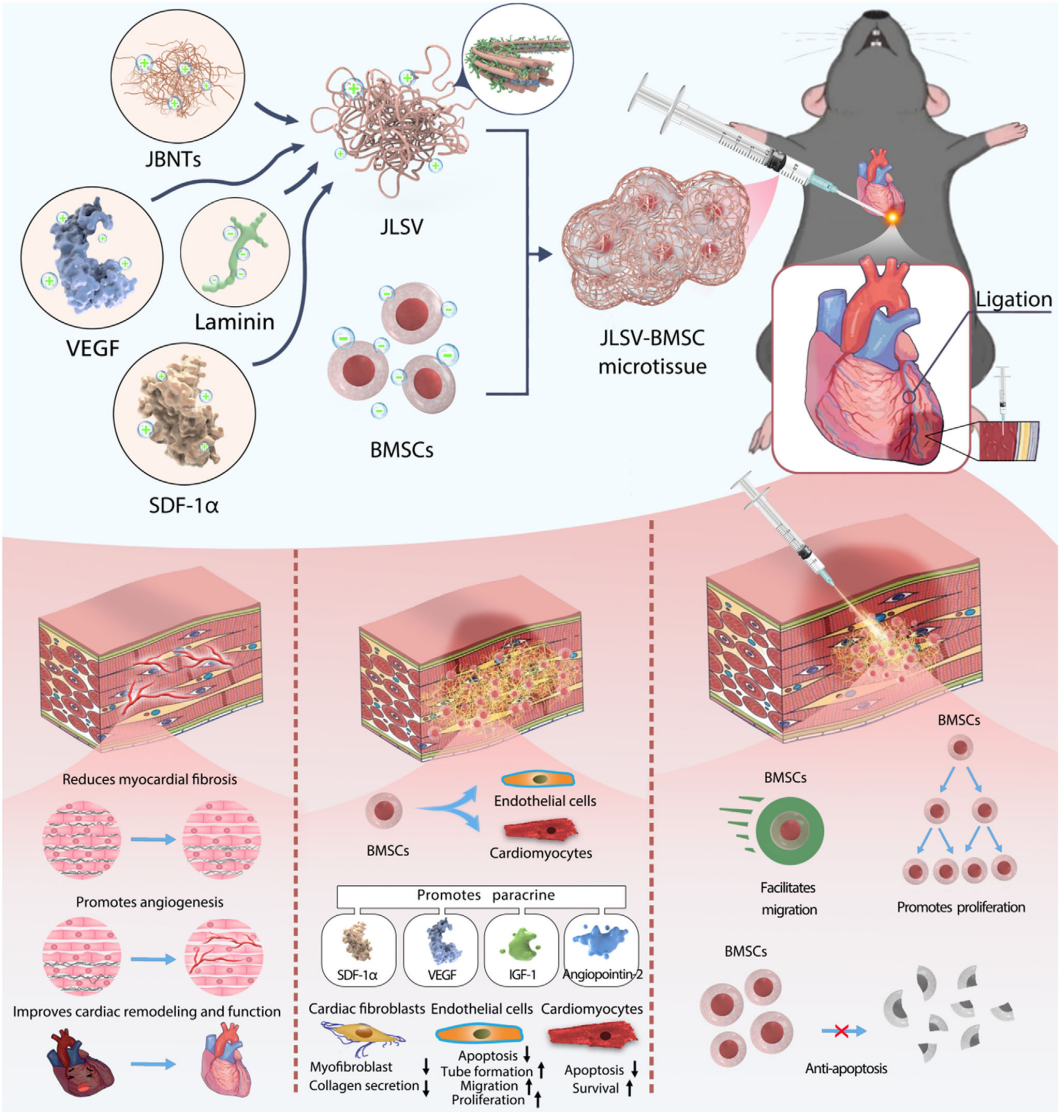
Schematic illustration of a dual biomimetic extracellular matrix scaffold (JLSV) consisting of Janus Basic Nanotubes, laminin, stromal‐derived factor‐1 alpha, and vascular endothelial growth factor for bone marrow‐derived mesenchymal stem cell (BMSC) delivery (JLSV–BMSC microtissue) for treating myocardial infarction. The JLSV–BMSC microtissue provides a beneficial microenvironment for cardiac repair by improving BMSC retention, directing BMSC differentiation, facilitating paracrine signaling modulation, protecting BMSCs from apoptosis, reducing myocardial fibrosis, and promoting angiogenesis.

To overcome these challenges, this study presents a novel injectable system combining a biomimetic scaffold consisting of laminin, stromal‐derived factor‐1 alpha (SDF‐1α), and vascular endothelial growth factor (VEGF) linked to Janus Basic Nanotubes (JBNTs) via electrostatic interactions (JLSV)^[^ ^22^, ^23^ ^]^ and BMSCs to create a 3D microtissue (JLSV–BMSC microtissue) for MI repair. Laminin, an ECM protein, plays a crucial role in regulating cell phenotypes and differentiation.^[^ ^24^ ^]^ SDF‐1α, an important stem cell homing factor, promotes stem cell retention and survival.^[^ ^25^ ^]^ VEGF promotes endothelial cell proliferation and migration ^[^ ^6^, ^26^ ^]^, thereby promoting angiogenesis at infarct sites.^[^ ^27^ ^]^ This study demonstrates that the JLSV–BMSC microtissue creates a dual biomimetic ECM‐like environment, which enhances BMSC viability, proliferation, and differentiation, enabling the effective repair of infarcted myocardium. Unlike traditional hydrogels, microtissues do not restrict BMSC migration, allow cells to migrate into the infarct region, and improve the repair efficacy (**Figure** **1**).

## 2 Results

### 2.1 JLSV–BMSC Microtissue Characterization

**Figure 2:**
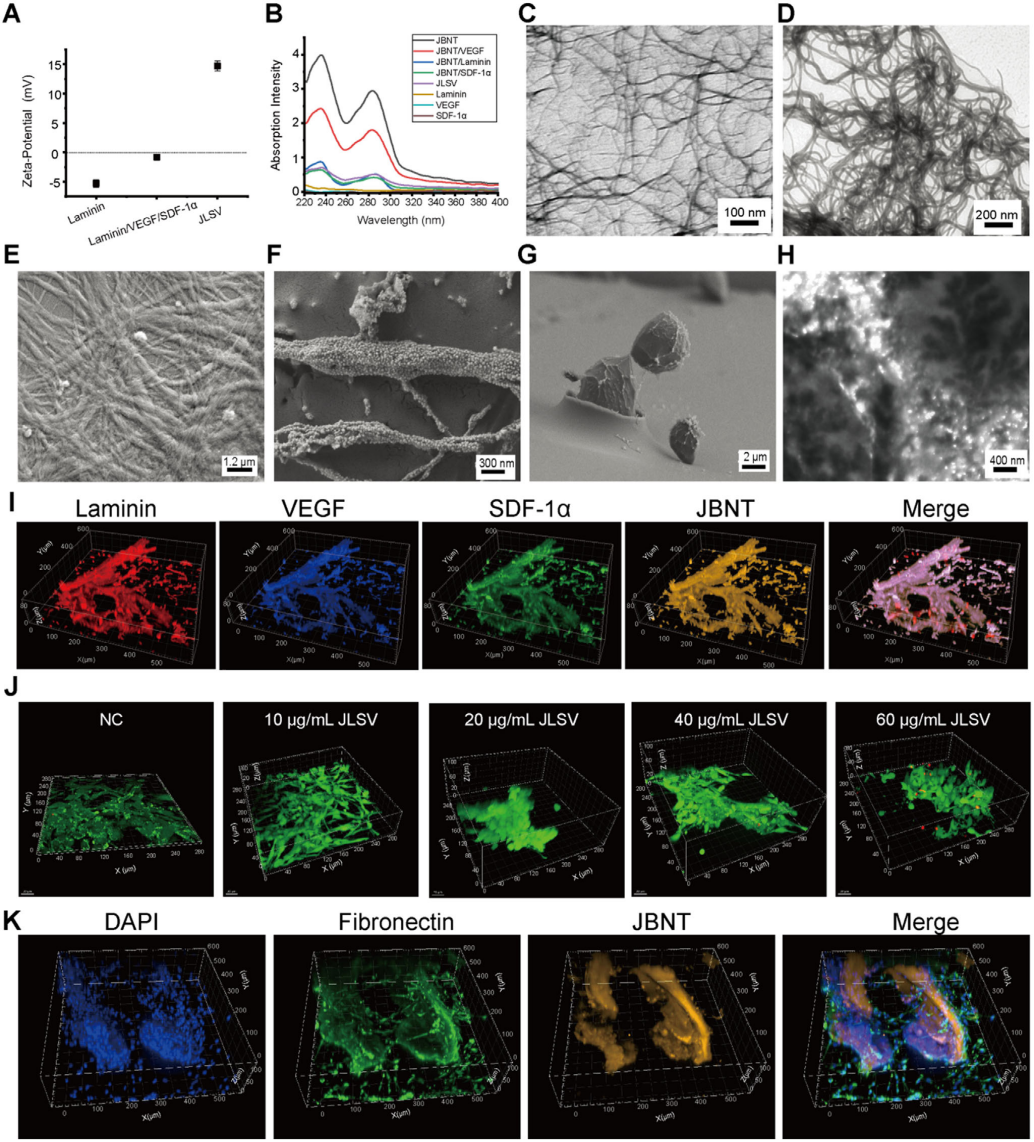
Characterization of a dual biomimetic extracellular matrix scaffold (JLSV) consisting of Janus Basic Nanotubes (JBNTs), laminin, stromal‐derived factor‐1 alpha (SDF‐1α), and vascular endothelial growth factor for bone marrow‐derived mesenchymal stem cell (BMSC) delivery (JLSV–BMSC microtissue). A) Zeta potential measurement of laminin, laminin/VEGF/SDF‐1α mixture, and JLSV. B) Ultraviolet–visible absorption spectra of laminin, VEGF, SDF‐1α, JBNT, JBNT/VEGF, JBNT/laminin, JBNT/SDF‐1α complex, and JLSV. C) Transmission electron microscopy (TEM) image showing the structure of JBNTs. D) TEM image showing the morphology of JLSV. E) Scanning electron microscopy (SEM) image of JLSV. F) Cryo‐SEM image of JLSV. G) Cryo‐SEM image showing JLSV–BMSC microtissue morphology. H) TEM images showing the JLSV–BMSC microtissue. I) 3D confocal images showing laminin–Alex Fluor 555/VEGF–Alex Fluor 674/SDF‐1α–Alex Fluor 488 staining/JBNTs‐Cy7. J) 3D‐reconstructed confocal images showing calcein‐AM (green) /PI (red; propidium iodide) staining of JLSV–BMSC microtissue. K) 3D confocal images showing nuclei counterstained with DAPI (blue), immunocytochemical staining for fibronectin (green), and JBNTs‐Cy7 (yellow).

To successfully assemble the JLSV scaffold, ≈16:4:1 (w/w/w) laminin α4 (111 kDa), VEGF165 (38 kDa), and SDF‐1α (8 kDa) were used based on their molecular weights. This ratio ensured that the molar amounts of each protein within the scaffold were approximately equivalent. The scaffold assembly was facilitated by electrostatic interactions, as verified by zeta potential measurements. The zeta potential of only laminin was negative (–5.28 mV; **Figure** **2**A). After proportionally adding the two positively charged proteins, VEGF and SDF‐1α, under physiological conditions, the zeta potential shifted to –0.582 mV. This change in potential confirmed protein complex formation, while ensuring that the resulting complex remained negatively charged, facilitating further self‐assembly under physiological conditions with positively charged JBNT.

The amount of JBNT used in the scaffold design must be carefully considered from two key perspectives. First, a fully assembled scaffold must carry a net positive charge to facilitate cell adhesion. Second, the proteins within the scaffold should remain within the effective concentration range (ng mL^−1^).^[^ ^26^, ^28^ ^]^ JBNT must also stay within its effective and safe concentration range (10–40 µg mL^−1^).^[^ ^29^ ^]^ By integrating these considerations with the zeta potential results, we ultimately determined the final mass ratio of the scaffold components to be 2000:16:4:1, resulting in a final zeta potential of the scaffold at 14.8 mV.

Ultraviolet–visible absorption spectroscopy confirmed the successful binding of the proteins to JBNTs, as evidenced by the reduction in the peaks at 230 and 280 nm (Figure 2B). The peaks at 230 and 280 nm are characteristic of the intrinsic absorption properties of JBNTs.^[^ ^23a,b^ ^]^ The peaks at 230 and 280 nm are mainly owing to the absorption of peptide bonds and are associated with the π–π transitions of aromatic rings within the JBNT structure, respectively. These absorption features are crucial for understanding the baseline optical properties of JBNTs prior to their interaction with proteins.

When proteins, such as laminin, are grafted onto JBNTs, noncovalent interactions, such as π–π stacking, hydrogen bonding, and electrostatic interactions, take place between the proteins and JBNTs. These interactions change the electronic environment of the JBNTs, affecting their UV–Vis absorption properties. Specifically, π–π stacking interactions between the aromatic rings of JBNTs and aromatic regions of the proteins can redistribute the electron density, commonly reducing the 280 nm absorption peak intensity. Similarly, hydrogen bonds or electrostatic interactions between protein functional groups and JBNTs can induce structural changes in JBNTs, further reducing the intensity of both 230 and 280 nm absorption peaks.

In addition, when JBNTs assemble with proteins, they aggregate or form large molecular complexes, which can alter the optical properties of the system and contribute to the observed decrease in absorption intensity. Thus, the reduction in peak intensity serves as an indirect confirmation of successful protein binding to JBNTs. Under transmission electron microscopy (TEM), the JBNTs exhibited a hair‐like fibrous morphology (Figure 2C). The material particle sizes were measured to ensure efficient injectability and flowability. The results indicated an average particle size of 326.9 nm (Figure S1D, Supporting Information). Additionally, the JBNTs were longer than 327 nm (Figure 2C). This discrepancy is primarily because during the Dynamic Light Scattering (DLS) particle size measurement, we reduced the material concentration to 10 µg mL^−1^ to achieve a uniform material size for easier measurement. However, a higher concentration than that used for size measurement was employed in TEM testing to clearly observe the material structure (200 µg mL^−1^), resulting in greater self‐assembly and larger size. When a material combines with proteins to form a scaffold, its size increases further to the micron level (Figure 2E), rendering it suitable for cell binding. From these results, we can see that JBNT, as a self‐assembled material, varies in size depending on the environment, but the morphology of the material consistently maintains a fibrous structure. Scaffolds formed with proteins are based on relatively weak noncovalent bonds. Materials formed based on noncovalent bonds typically have a low hardness and elastic modulus, ensuring injectability and flowability. The elastic modulus of JLSV was relatively low (Figure S1B,C, Supporting Information), indicating that it is soft and has the potential for injectability. To further verify the injectability of the JBNT, we used a 30G insulin needle for injection (Figure S1F, Supporting Information). Under this needle specification, JBNT demonstrated excellent injectability. The morphology of the JLSV scaffold formed by assembling JBNTs, SDF‐1α, VEGF, and laminin was examined by TEM, scanning electron microscopy (SEM), and cryo‐scanning electron microscopy (cryo‐SEM, Figure 2D–F). In contrast to the hair‐like morphology of JBNTs, the JLSV scaffold assembled into thick bundles after incorporating the three proteins. To further investigate the interactions between JBNTs and these proteins, VEGF, SDF‐1α, laminin, and JBNTs were labeled with blue, green, red fluorescent dyes and a yellow Sulfo‐Cyanine7 (Cy7) fluorescent dye, respectively (Figure 2I). 3D reconstruction by confocal laser scanning microscopy (CLSM) showed the binding of these proteins to the scaffold, with no significant fluorescence detected outside the scaffold. This indicated that all three proteins were successfully anchored to the JBNTs, confirming that JBNTs can simultaneously bind to multiple cytokines via electrostatic adsorption. To clarify the release characteristics of active proteins within JLSV, we conducted a systematic study using a dialysis setup with a 500 kDa membrane. This method allows smaller active proteins to diffuse through the membrane, while retaining larger JLSV scaffold proteins. The study was performed over 1, 3, 7, 14, and 28 days using ELISA kits to measure the protein concentrations inside and outside the membrane. The results showed that the protein concentrations inside the dialysis bag were substantially higher than those outside the bag (Figure S1E, Supporting Information), suggesting a minimal release and diffusion of proteins during the study period. This finding highlights that active proteins remain largely associated with the JLSV scaffold, preserving their biological activity and maintaining a suitable biomimetic ECM environment for BMSC culture and bioinduction.

**Figure 3:**
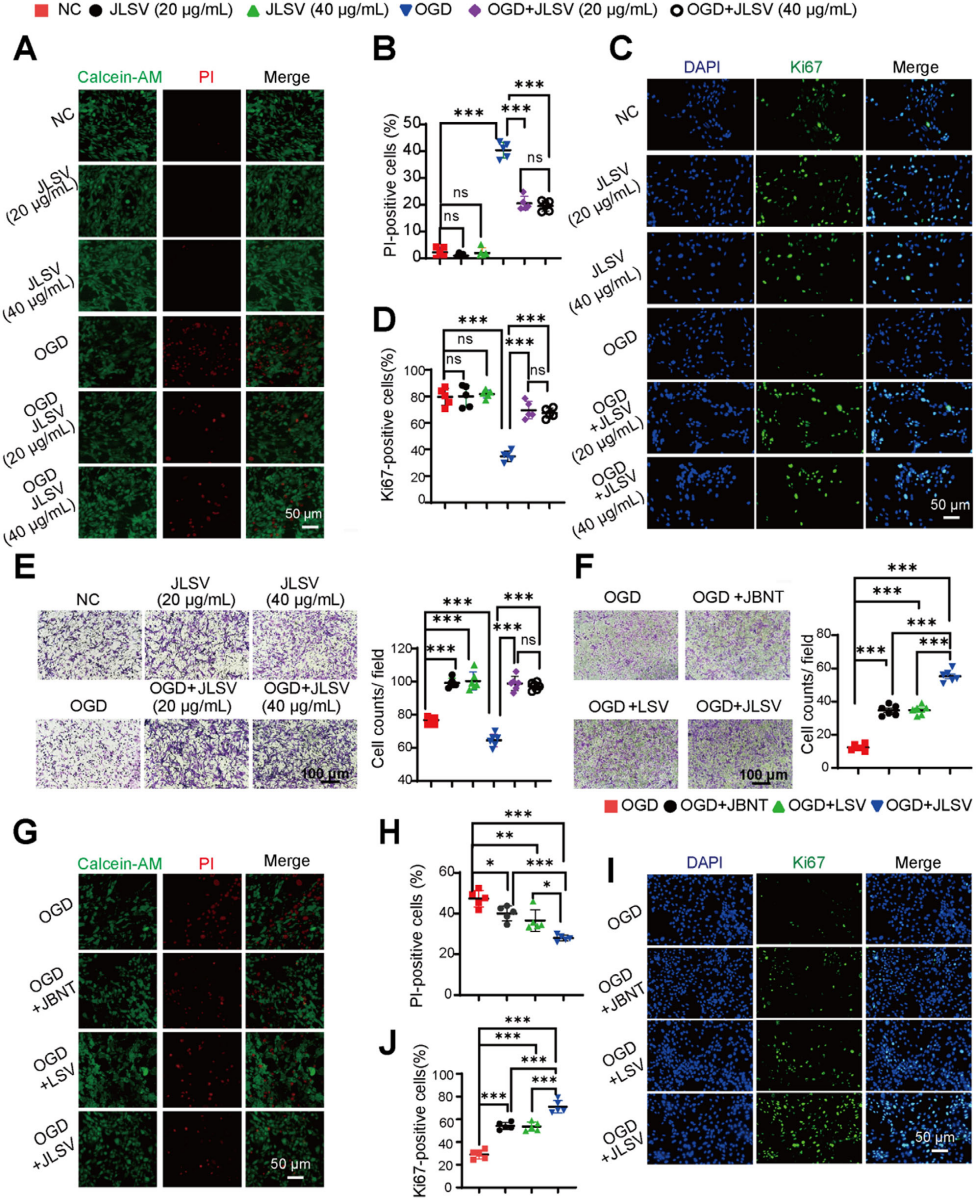
A dual biomimetic extracellular matrix scaffold (JLSV) consisting of Janus Basic Nanotubes, laminin, stromal‐derived factor‐1 alpha, and vascular endothelial growth factor enhances bone marrow‐derived mesenchymal stem cell (BMSC) survival, proliferation, and migration under oxygen–glucose deprivation (OGD) conditions. A and B) Calcein‐AM (live) /propidium iodide (PI, dead) staining was used to assess BMSC viability with or without different concentrations of JLSV after OGD injury or under normal conditions. Representative images are shown in (A), and the quantitative data are summarized in (B). C) Representative images of Ki67 staining (green: Ki67‐positive cells; blue: DAPI‐stained nuclei) were used to evaluate the proliferation ability of BMSCs with or without different JLSV concentrations after OGD injury or under normal conditions. The quantitative data are summarized in (D). E) The BMSC migration assay was performed to evaluate the migration capacity of BMSCs incubated with different concentrations of JLSV or control media under OGD or normal conditions. Quantitative results are summarized. F) BMSC migration was compared after treatment with individual components of JLSV, including JBNT, LSV, complete JLSV, or control media under OGD conditions. G) Calcein‐AM (live) /PI (dead) staining was used to determine the viability of BMSCs treated with individual components of JLSV or control media under OGD conditions. The quantitative results are summarized in (H). I) Representative images of Ki67 staining (green: Ki67‐positive cells; blue: DAPI‐stained nuclei) to assess the proliferation ability of BMSCs treated with individual components of JLSV or control media under OGD conditions. The quantitative data are summarized in (J). All ^*^ *p* < 0.05, ^**^ *p* < 0.01, ^***^ *p* < 0.001; n=5.

The JLSV–BMSC microtissue structure was examined using TEM and cryo‐SEM (Figure 2G,H). The results showed that the JLSV scaffold effectively enveloped BMSCs and facilitated their aggregation, resulting in a cohesive structure. To evaluate the effects of different JLSV concentrations on BMSC aggregation and viability, live‐dead staining and 3D reconstruction were performed using CLSM. Four concentrations of JLSV (10, 20, 40, and 60 µg mL^−1^) were tested, each concentration being composed according to the proportions of the components determined by zeta potential measurements. The 3D reconstruction revealed that 10 µg mL^−1^ BMSCs did not form effectively aggregate. In contrast, 20, 40, and 60 µg mL^−1^ BMSCs successfully formed 3D microtissues (Figure 2J). However, live/dead staining showed that dead cells were observed with 60 µg mL^−1^ BMSCs (Figure S1A, Supporting Information), which may be attributed to the fact that JBNT carries a positive charge under physiological conditions (Figure 2A). The positive charge on the material surface makes it more likely to bind to negatively charged cell membranes. Some material may enter the cells through endocytosis, which is enhanced as the concentration increases, thus increasing cell membrane disruption and cell death.^[^ ^29^, ^30^ ^]^

Thus, a combination of 20 or 40 µg mL^−1^ JLSV and BMSCs successfully formed 3D microtissues, and cell viability remained high. Therefore, 20 and 40 µg mL^−1^ JLSV were selected for further experiments.

To further evaluate the formation of microtissues, we labeled JBNTs with Cy7 and performed immunofluorescent staining for fibronectin, a key extracellular matrix protein. Cell nuclei were counterstained with DAPI, and observations were conducted using confocal microscopy with 3D imaging. Confocal 3D imaging revealed that fibronectin, secreted by BMSCs and anchored to the JLSVs scaffold, was effectively immobilized on the scaffold (Figure 2K). The sequestration of fibronectin by the scaffold, combined with the scaffold‐supported cells, cooperatively promoted tissue structure development and associated functions. These findings collectively demonstrate the successful formation of a robust biomimetic microtissue. Additionally, to investigate whether BMSCs could phagocytize JLSV, we stained the cytoskeleton with rhodamine phalloidin, labeled JBNTs (the crucial component of JLSV) with Cy7, and counterstained nuclei with DAPI (Figure S1H, Supporting Information). The results showed no evidence of BMSCs phagocytizing JLSV. To further investigate the mechanical properties of JLSV, we conducted a rheological assessment of its viscoelastic characteristics using a stress rheometer (Figure S1B,C, Supporting Information). The rheometer measured the changes in the storage modulus (G′) and loss modulus (G″) under various angular frequencies and strain amplitudes. The results indicate that JLSV exhibits G′ greater than G″, suggesting that the elastic behavior of the material is dominant. This implies that it behaved more like a solid, storing most of its applied energy instead of dissipating it. Figure S1B (Supporting Information) shows that the modulus of JLSV increases with frequency across 0.1–100 rad s^−1^, highlighting a key feature of its viscoelastic response. This frequency‐dependent behavior indicates that the material can dynamically respond to mechanical stimuli, which is crucial for its integration into biological tissues. Additionally, the material maintains a constant G' under low‐strain conditions (Figure S1C, Supporting Information), exhibiting solid‐like elasticity that helps preserve form and function within biological environments. As the applied strain increases, G' decreases, indicating a transition toward fluid‐like behavior owing to structural disruption. Based on these findings, JLSV is a flexible solid cell scaffold dispersed in a solution.

### 2.2 In Vivo Metabolism and Degradation of JLSV

To assess the biodegradability and safety of JLSV in vivo, we labeled it with the fluorescent dye Cy7 and imaged using the IVIS Kinetic System. The mice were divided into six groups: Sham+Cy7, Sham+JLSV–Cy7, Sham+JLSV–Cy7–BMSCs, MI+Cy7, MI+JLSV–Cy7, and MI+JLSV–Cy7–BMSCs. The mice received intramyocardial injections and underwent both in vivo and *ex vivo* fluorescence imaging using the IVIS Spectrum in vivo imaging system at five specific time points: 1, 3, 7, 14, and 28 days post‐MI. The fluorescence intensities in the Sham+JLSV–Cy7–BMSCs and MI+JLSV–Cy7–BMSCs groups were consistently higher and more persistent than in the JLSV–Cy7 group alone (Figures S2 and S3, Supporting Information), indicating that the BMSCs enhanced material retention. When materials and cells form microtissues, the material retention duration at the injection site is significantly extended. This also proves from a new perspective that after microtissues are injected into the body, the cells and scaffold still interact closely within the body.

*Ex vivo* imaging of major organs confirmed that the heart consistently exhibited the strongest fluorescent signals, indicating the primary localization of the scaffold at the target site. Although faint signals were detected in the liver and kidney, they were much weaker than those in the myocardium, suggesting that while the scaffold degrades locally within the cardiac tissue, minor degradation products may be cleared via the liver and kidney.

In addition, the fluorescence signal in the myocardium gradually weakened and was almost undetectable by day 28. This suggests that the scaffold underwent gradual and complete degradation within ≈4 weeks post‐injection, matching the tissue remodeling response after early MI. This localized, time‐limited degradation minimizes the risk of long‐term material accumulation and systemic toxicity.

Additionally, to evaluate the systemic biosafety of the degrading scaffold, blood samples were collected from all six animal groups at five time points for comprehensive analyses, including routine blood tests, liver and kidney function tests, and inflammation marker evaluation. No significant differences in liver and kidney functions or routine blood tests were observed among the groups at any time point (Tables S1–S10, Supporting Information), further supporting the biocompatibility and safety of the JLSV scaffold and its degradation products. As shown in Table S11 (Supporting Information), the MI group exhibited significantly elevated IL‐6 and TNF‐α levels at all time points than the Sham group, and the MI+JLSV–BMSC microtissue group showed a significant decrease in IL‐6 and TNF‐α than the MI group, indicating that JLSV–BMSC microtissue effectively alleviates post‐MI inflammation.

Our findings suggest that JLSV–BMSC microtissues undergo controlled biodegradation primarily within the myocardium with minimal systemic distribution and hepatic clearance, supporting their potential to provide sustained regenerative benefits while ensuring biosafety.

### 2.3 JLSV Enhances BMSC Survival, Proliferation, and Migration Under Oxygen–Glucose Deprivation Conditions

To determine the optimal JLSV scaffold concentration for enhancing BMSC survival, migration, and proliferation under ischemic and hypoxic conditions, a series of assays was performed in vitro under oxygen–glucose deprivation (OGD) conditions mimicking ischemic and hypoxic microenvironments. The effects of 20 and 40 µg mL^−1^ JLSV on BMSCs under both normal and OGD conditions were evaluated. Live/dead staining revealed no significant differences in cell viability between the JLSV and vehicle control (phosphate‐buffered saline, PBS) groups under normal conditions, suggesting that the scaffold is safe for BMSC culture. However, under OGD conditions, both JLSV doses significantly improved BMSC viability than PBS, with no significant difference observed between the two concentrations (**Figure** **3**A,B; Figure S4A, Supporting Information).

Ki67 staining was performed to assess the effects of JLSV on BMSC proliferation. Under normal conditions, the percentage of Ki67‐positive cells was similar in all the groups. However, under OGD conditions, the percentage of Ki67‐positive cells was significantly higher in both JLSV groups than in the vehicle control group, with no significant difference between the two JLSV concentrations (Figure 3C,D), suggesting that JLSV promotes BMSC proliferation under OGD conditions. To comprehensively evaluate the pro‐proliferative potential of JLSV under normoxic conditions, we extended the culture duration to 24, 48, and 72 h and quantified cellular proliferation using the CCK‐8 assay. These analyses demonstrated that JLSV enhanced BMSC proliferation throughout the extended culture period (Figure S5G, Supporting Information). A Transwell migration assay was performed to evaluate the effect of JLSV on BMSC migration. Under normal conditions, BMSC migration rates were comparable for both JLSV concentrations and higher than those in the control group. Under OGD conditions, both JLSV concentrations enhanced BMSC migration to a similar extent (Figure 3E).

In summary, both 20 and 40 µg mL^−1^ JLSV significantly improved BMSC survival, proliferation, and migration under OGD conditions. Since both concentrations showed similar efficacy, 20 µg mL^−1^ was chosen as the optimal concentration for the subsequent experiments. In addition, the safety of the JLSV scaffold was confirmed under normal conditions.

To further investigate the individual contributions of the scaffold components under OGD conditions, JLSV was divided into two constructs: one containing the bioactive proteins (laminin, SDF‐1α, and VEGF, abbreviated as LSV) and the other consisting only of JBNTs. In the transwell migration assay, under OGD conditions, all three groups (JLSV, LSV, and JBNT) showed increased migration than the vehicle control group, but both JBNT and LSV groups showed fewer migrated cells than the JLSV group (Figure 3F). Transcriptome sequencing revealed increased expression of integrin α2 (Itga2) mRNA in JLSV‐treated BMSC microtissues compared to BMSC monocultures (Figure S8D, Supporting Information). To further investigate this phenomenon, BMSCs under OGD conditions were treated with JBNT, LSV, JLSV, or control medium. Western blot analysis revealed significantly elevated Itga2 protein expression in the OGD+JBNT, OGD+LSV, and OGD+JLSV groups relative to the OGD control group (Figure S8F, Supporting Information). This Itga2 upregulation may enhance the migratory capacity of JBNT‐treated cells under OGD conditions. We hypothesize that JLSV provides physical anchoring sites for cells, interacting with extracellular matrix receptors (such as integrins) on the cell membrane. Such interactions potentially regulate cytoskeletal dynamics and trigger outside‐in signaling cascades. Live/dead staining showed that the JLSV group had the highest percentage of viable cells (green), whereas both JBNT and LSV groups showed lower viability than the JLSV group under OGD conditions (Figure 3G, H; Figure S4B, Supporting Information), further indicating the superior effect of the full scaffold in improving the BMSC survival under OGD conditions.

Similarly, Ki67 staining under OGD conditions showed that all three groups exhibited increased BMSC proliferation than the vehicle control group. However, both JBNT and LSV groups exhibited lower BMSC proliferation than the JLSV group, suggesting that the complete scaffold provided the necessary synergistic effects to maximize BMSC proliferation (Figure 3I,J).

These results emphasize the crucial role of the combined action of bioactive proteins and JBNTs in the JLSV scaffold. This synergistic effect was likely owing to the ability of JBNTs to anchor multiple cytokines via electrostatic adsorption, which increased their stability in physiological solutions and prolonged their active lifetime. This biological support is critical for promoting BMSC survival, proliferation, and migration under OGD conditions. Furthermore, the JLSV scaffold mimicked the natural ECM morphology and structure, providing physical support for the BMSCs.

In summary, the JLSV scaffold exhibited highly biomimetic structural and biological properties, and significantly enhanced BMSC survival, proliferation, and migration under ischemic and hypoxic conditions. The integrated design of the JLSV scaffold created a supportive environment that enhanced the therapeutic potential of stem cell‐based therapies.

### 2.4 JLSV Reduces BMSC Apoptosis and Promotes Differentiation Under OGD Conditions

The effects of the JLSV scaffold on BMSC apoptosis and differentiation under OGD conditions were further investigated. To assess apoptosis, we analyzed the levels of apoptosis‐related proteins, including Bax, Bcl2, and cleaved caspase 3, by western blotting and complemented these results with the Annexin V–FITC/PI staining assay.

**Figure 4:**
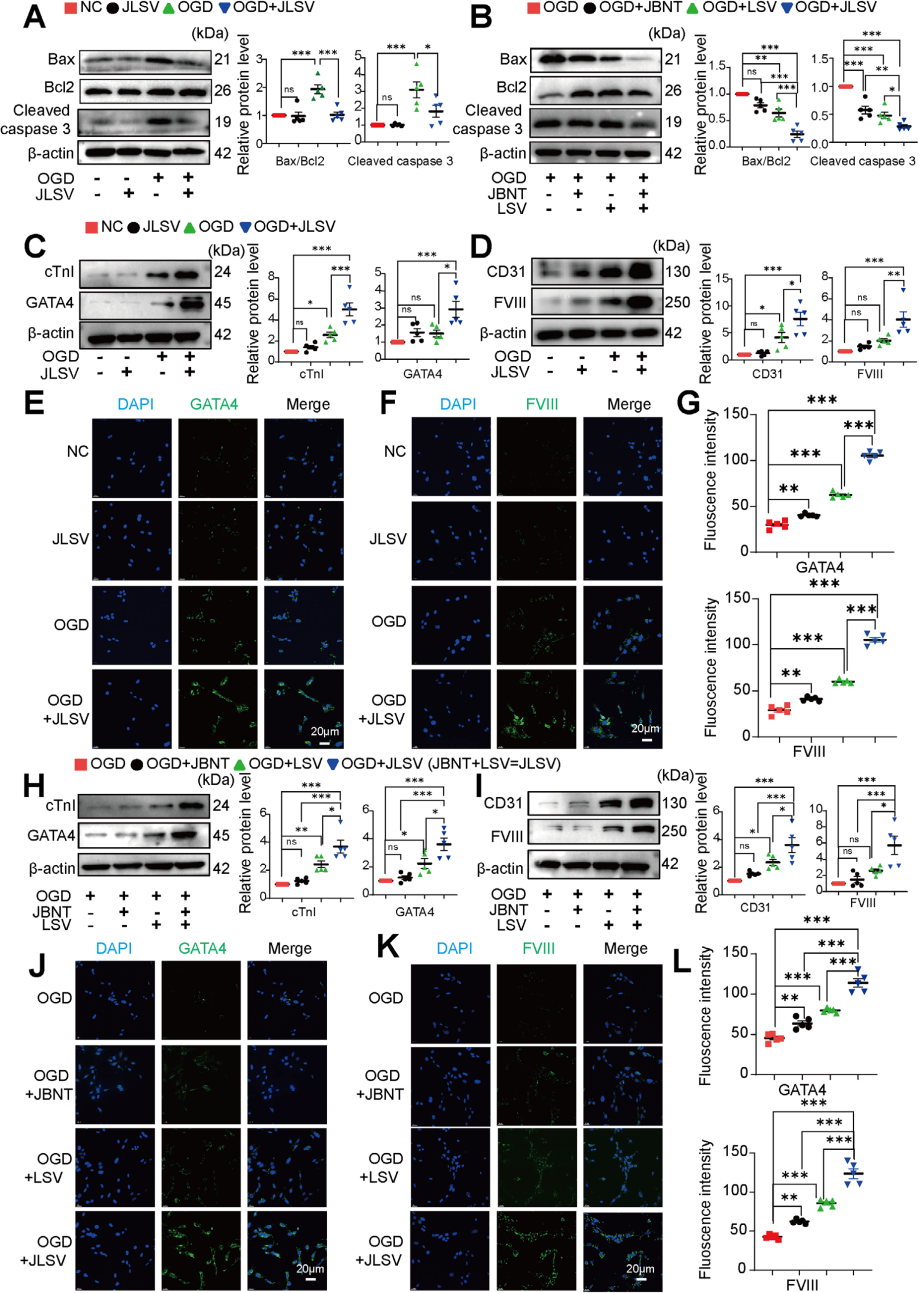
A dual biomimetic extracellular matrix scaffold (JLSV) consisting of Janus Basic Nanotubes (JBNTs), laminin, stromal‐derived factor‐1 alpha, and vascular endothelial growth factor (LSV) for bone marrow‐derived mesenchymal stem cell (BMSC) delivery (JLSV–BMSC microtissue) reduces apoptosis and promotes BMSC differentiation under oxygen–glucose deprivation (OGD) conditions. A) BMSCs were cultured with or without JLSV exposure under either OGD or normal conditions. Bax, Bcl2, and cleaved caspase‐3 protein levels were measured by western blotting. Data represent the mean ± standard error of the mean (n=5). B) BMSCs were incubated with individual components of JLSV, including JBNT, LSV, complete JLSV (JBNT plus LSV), or control media under OGD conditions. Bax, Bcl2, and cleaved caspase‐3 protein levels were determined by western blotting. Data represent the mean ± standard error of the mean, n=5. C) BMSCs were cultured with or without JLSV exposure under OGD or normal conditions. Protein levels of cardiomyocyte markers (GATA4 and cTnI) were measured by western blotting. Data represent the mean ± standard error of the mean, n=5. D) BMSCs were cultured with or without JLSV exposure under OGD or normal conditions. Protein levels of endothelial cell markers (FVIII and CD31) were measured by western blotting. Data represent the mean ± standard error of the mean, n=5. E) BMSCs were cultured with or without JLSV exposure under OGD or normal conditions. Immunocytochemical staining was performed to detect cardiomyocyte markers (GATA4, green), with nuclei counterstained with DAPI (blue). F) BMSCs were cultured with or without JLSV exposure under OGD or normal conditions. Immunocytochemical staining was performed to detect endothelial cell markers (FVIII, green), with nuclei counterstained with DAPI (blue). G) Quantitative data were obtained from five independent images per group and expressed as mean ± standard error of the mean, n=5. H) BMSCs were incubated with individual components of JLSV, including JBNT, LSV, complete JLSV, or control media under OGD conditions. Protein levels of cardiomyocyte markers (GATA4 and cTnI) were measured by western blotting. Data represent the mean ± standard error of the mean, n=5. I) BMSCs were incubated with individual components of JLSV, including JBNT, LSV, complete JLSV, or control media under OGD conditions. Protein levels of endothelial cell markers (FVIII and CD31) were measured by western blotting. Data represent the mean ± standard error of the mean, n=5. J) BMSCs were incubated with individual components of JLSV, including JBNT, LSV, complete JLSV, or control media under OGD conditions. Immunocytochemical staining was performed to detect cardiomyocyte markers (GATA4, green), with nuclei counterstained with DAPI (blue). K) BMSCs were incubated with individual components of JLSV, including JBNT, LSV, complete JLSV, or control media under OGD conditions. Immunocytochemical staining was performed to detect endothelial cell markers (FVIII, green), with nuclei counterstained with DAPI (blue). L) Quantitative data were obtained from five independent images per group and expressed as mean ± standard error of the mean, n=5.

Under normal conditions, the expression of these apoptotic markers was not significantly different between the BMSCs cultured with the JLSV scaffold and the control group, suggesting that the scaffold did not induce apoptosis in a normal environment, further supporting its safety profile. In contrast, BMSCs cultured with the JLSV scaffold under OGD conditions showed a significant reduction in apoptosis markers than the vehicle control group. Western blot analysis showed a significant decrease in the Bax/Bcl2 ratio and cleaved caspase 3 protein levels, while Annexin V–FITC/PI staining showed a significant decrease in both early and late apoptotic cells. These results suggested that the scaffold exerted a protective effect and protected BMSCs from apoptosis under OGD conditions (**Figures** **4**A and S5A, Supporting Information).

To investigate the effect of JLSV on oxidative stress, we examined the production of superoxide anions using dihydroethidium staining (DHE). The results showed that superoxide anion levels were significantly reduced in BMSCs cultured with the JLSV scaffold than in the vehicle control OGD group (Figure S5C, D, Supporting Information). Overall, these results suggested that the JLSV scaffold improved BMSC survival by reducing oxidative stress and apoptosis, thereby increasing overall cell viability and functional capacity under OGD conditions. To assess the stability of JLSV under oxidative stress, we labeled the scaffold with Alexa Fluor 555, which emits a detectable red fluorescence. JLSV samples were incubated with PBS containing 5 mM hydrogen peroxide (H_2_O_2_) to simulate oxidative stress. We evaluated the changes in morphology and fluorescence intensity 1, 3, 7, 14, and 28 days

After 28 days, the JLSV group displayed minimal changes in both structural morphology and fluorescence intensity than the vehicle control group (Figure S1G, Supporting Information). This suggests that JLSV retained its structural integrity in the presence of H_2_O_2_, demonstrating its resistance to oxidative degradation, which typically intensifies in post‐MI environments. Next, we investigated the roles of the individual components of the JLSV scaffold by dividing it into two constructs: one consisting only of bioactive proteins (LSV) and the other consisting exclusively of JBNTs. We examined the effects of these individual components on BMSC apoptosis under OGD conditions and compared the results with those of the full JLSV scaffold. The results showed that JBNT, LSV, and JLSV protected BMSCs from apoptosis, whereas LSV and JBNT showed limited efficacy in reducing BMSC apoptosis under OGD conditions. Quantitative analyses of Bax/Bcl2, cleaved caspase 3, and Annexin V–FITC/PI staining confirmed that the individual components were less effective in attenuating apoptosis than the complete JLSV scaffold (Figure 4B and Figure S5B, Supporting Information). DHE staining showed similar results, emphasizing the superior ability of the complete scaffold to reduce oxidative stress (Figure S5E, F, Supporting Information). These results emphasized the need for synergy between proteins and JBNTs, as neither component alone replicated the improved cell survival observed with the full scaffold.

In addition to cell survival, we investigated the ability of the JLSV scaffold to promote the BMSC differentiation into specific cell lineages. Western blotting and immunocytochemical staining were performed to measure the expression of key differentiation markers. GATA4 and cardiac troponin I (cTnI), two essential transcription factors and structural proteins in cardiomyocytes, respectively, were quantified. Two important endothelial cell markers, factor VIII (FVIII) and CD31, were analyzed.

Western blotting and immunocytochemical staining analyses showed a significant increase in GATA4 and cTnI expression in BMSCs cultured with the JLSV scaffold than in the BMSCs cultured with the negative control, indicating increased BMSC differentiation toward the cardiomyocyte lineage (Figure 4C, E, and Figure S6A, C, Supporting Information). Similarly, the increased FVIII and CD31 levels indicated that the JLSV scaffold promoted BMSC differentiation toward the endothelial lineage (Figure 4D, F, and Figure S6B, C, Supporting Information). These results were confirmed by the strong fluorescent signals of these markers, supporting the conclusion that the JLSV scaffold promoted differentiation. Remarkably, this differentiation occurred even under OGD‐induced stress, demonstrating its potential to promote the development of lineage‐specific cells in unfavorable environments. To investigate whether BMSCs exhibit mature cardiomyocyte characteristics in vitro within the JLSV microenvironment, we cultured JLSV–BMSC microtissues for up to 21 days. To examine their differentiation status, we conducted immunofluorescence staining with cardiomyocyte‐specific markers, including α‐actinin, cTNI, and connexin 43 (CX43) (Figure S7, Supporting Information). These markers were positively expressed, confirming that BMSCs underwent cardiomyogenic differentiation at the protein level. However, the absence of spontaneous beating suggests that these cells remain in an immature functional state, which may be primarily owing to constraints of the in vitro culture environment. Unlike the dynamic in vivo microenvironment^[^ ^31^ ^]^, which is rich in complex biomechanical^[^ ^32^ ^]^, electrical^[^ ^33^ ^]^, and biochemical signals^[^ ^34^ ^]^, our static in vitro conditions may not provide the necessary support for the full maturation of differentiated cells.

To further investigate the effects of the individual components of the JLSV scaffold on BMSC differentiation under OGD conditions, we divided the scaffold into LSV and JBNT constructs. Western blotting and immunocytochemical staining analyses consistently revealed lower of GATA4, cTnI, FVIII, and CD31 levels in BMSCs cultured with the individual components than in those exposed to the integrated JLSV scaffold (Figure 4H–L and Figure S6E, F, Supporting Information). These results suggest that under OGD conditions, the bioactive protein or JBNT partially promoted BMSC differentiation, but their effects were less pronounced than those of the complete JLSV scaffold.

The observed synergy probably results from the complementary roles of the two components. JBNTs provide a structural matrix that promotes cell adhesion and migration, whereas bioactive proteins provide molecular signals that activate specific signaling pathways. This bionic strategy is an essential component of the scaffold design, which, in combination with stem cells, offers a promising approach for creating microtissues. These microtissues not only reduce stem cell apoptosis but also promote targeted differentiation. This offers a potential solution for the problems of poor cell maintenance and differentiation in harsh microenvironments.

### 2.5 RNA Sequencing Reveals How JLSV Facilitates BMSC Differentiation into Cardiomyocytes

To investigate how JLSV promotes BMSC differentiation into cardiomyocytes, we conducted transcriptome analysis to compare untreated BMSCs with JLSV–BMSC microtissues (Figure S8A–D, Supporting Information). Gene Ontology (GO) enrichment analysis revealed that genes related to cell differentiation, angiogenesis, and tissue development were significantly upregulated after JLSV treatment. Kyoto Encyclopedia of Genes and Genomes (KEGG) pathway analysis showed that JLSV affected several signaling pathways regulating stem cell fate and differentiation, including the PI3K/AKT, AMPK, calcium, Rap1, ECM–receptor interaction, and WNT signaling pathways. Notably, the PI3K/AKT pathway exhibited the highest priority for gene enrichment, highlighting its central role in JLSV‐induced cardiomyogenic differentiation. RNA‐sequencing results revealed that JLSV upregulated genes associated with cardiomyocyte and endothelial cell differentiation, including placental growth factor (PGF)^[^ ^35^ ^]^ and fibroblast growth factor 2 (Fgf2).^[^ ^31^, ^36^ ^]^

To confirm the involvement of the PI3K/AKT pathway, we used an OGD model to simulate ischemic conditions and categorized the BMSCs into four groups: (1) OGD only, (2) OGD+AKT inhibitor, (3) OGD+JLSV, and (4) OGD+JLSV+AKT inhibitor. Western blot analysis revealed that JLSV significantly increased AKT phosphorylation at Ser473 (Figure S8E, Supporting Information), which is specifically regulated by mTOR complex 2 (mTORC2). This activation coincided with the enhanced expression of the cardiomyocyte‐specific markers cTnI and GATA4, indicating stimulated cardiac differentiation. Importantly, an AKT inhibitor (MK‐2206 2HCl) decreased AKT phosphorylation and cardiomyogenic marker expression, reinforcing the causal role of PI3K/AKT pathway in JLSV‐induced differentiation. Western blot analysis revealed that Pgf and Fgf2 protein expression was elevated in the OGD+JLSV group compared to the OGD group. This upregulation was reversed by co‐treatment with AKT inhibition. These results suggest that JLSV upregulates these proteins to modulate differentiation through the PI3K/AKT pathway.

In addition to intracellular signaling, we propose that JLSV enhances differentiation through ECM‐related and biomechanical mechanisms. As a biomimetic nanoscale scaffold, JLSV mimics the myocardial ECM, facilitating integrin signaling. The significant ECM–receptor interaction enrichment in the KEGG analysis supports the importance of ECM‐related signaling in JLSV‐induced cardiomyogenesis.

### 2.6 JLSV–BMSC Microtissue Promotes the Crosstalk of BMSCs and Other Cells in the Infarcted Myocardium Through Regulating Paracrine

**Figure 5:**
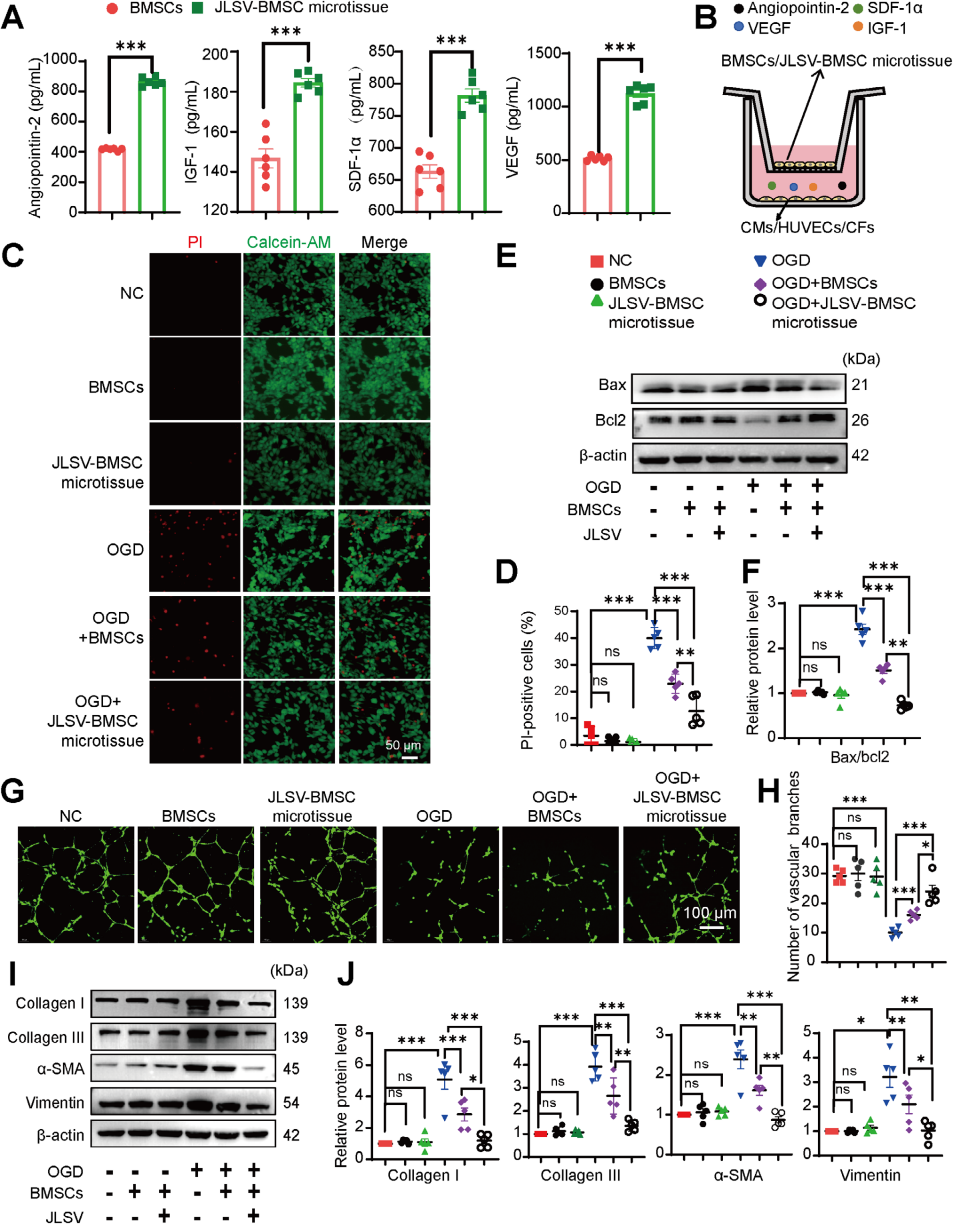
A dual biomimetic extracellular matrix scaffold (JLSV) consisting of Janus Basic Nanotubes (JBNTs), laminin, stromal‐derived factor‐1 alpha (SDF‐1α), and vascular endothelial growth factor (VEGF) for bone marrow‐derived mesenchymal stem cell (BMSC) delivery (JLSV–BMSC microtissue) promotes the crosstalk of BMSCs and other cells in the infarcted myocardium through regulating paracrine. A) Angiopoietin‐2, IGF‐1, VEGF, and SDF‐1α concentrations were measured using ELISA. Data represent the mean ± standard error of the mean (n=5). B) A schematic diagram illustrating the co‐culture of BMSCs or JLSV–BMSC microtissue with cardiomyocytes, human umbilical vein endothelial cells (HUVECs), and cardiac fibroblasts. C) Calcein‐AM (live) /propidium iodide (PI, dead) staining was used to assess the viability of cardiomyocytes cultured with BMSCs or JLSV–BMSC microtissue under oxygen–glucose deprivation (OGD) or normal conditions, and the quantitative results are summarized in (D). Data represent the mean ± standard error of the mean, n=5. ^*^ *p* < 0.05, ^**^ *p* < 0.01, ^***^ *p* < 0.001. E) Cardiomyocytes were cultured with BMSCs or JLSV–BMSC microtissue under OGD or normal conditions, and Bax and Bcl2 protein levels were measured by western blotting. The quantitative results are summarized in (F). Data represent the mean ± standard error of the mean, n=5. ^*^ *p* < 0.05, ^**^ *p* < 0.01, ^***^ *p* < 0.001. G) A tube formation assay was performed to evaluate the angiogenic effects of HUVECs cultured with BMSCs or JLSV–BMSC microtissue under OGD or normal conditions. Representative images are shown, and the quantitative results are presented in H). Data represent the mean ± standard error of the mean, n=5. ^*^ *p* < 0.05, ^**^ *p* < 0.01, ^***^ *p* < 0.001. I) Cardiac fibroblasts were cultured with BMSCs or JLSV–BMSC microtissue under OGD or normal conditions, and alpha‐smooth muscle actin (α‐SMA), vimentin, collagen I, and collagen III protein levels were measured by western blotting. The quantitative results are summarized in (J). Data represent the mean ± standard error of the mean, n=5. ^*^ *p* < 0.05, ^**^ *p* < 0.01, ^***^ *p*<0.001.

To evaluate the effects of JLSV–BMSC microtissue on the paracrine activity of BMSCs, an ELISA test was performed to measure the secretion of four key proteins: SDF‐1α, VEGF, insulin‐like growth factor 1 (IGF‐1), and angiopoietin‐2. Two experimental groups were studied: BMSCs cultured alone and JLSV–BMSC microtissues. The results showed significantly higher levels of all four proteins in the JLSV–BMSC microtissue group than in the BMSC group (**Figure** **5**A). To determine whether JLSV mediates paracrine secretion of SDF‐1α and VEGF from microtissues, we performed ELISA in conditioned media from three groups: BMSC‐only, JLSV‐only, and JLSV‐BMSC microtissues. The BMSC+JLSV group exhibited the highest secretion levels of both cytokines—significantly exceeding those from BMSC‐only and JLSV‐only groups (Figure S9G, Supporting Information). And the concentrations of SDF‐1α and VEGF secreted by the JLSV‐BMSC microtissues exceeded the additive total from separate BMSC‐only and JLSV‐only cultures (Figure S9H, Supporting Information). These results indicate that the JLSV scaffold enhances paracrine secretion of SDF‐1α and VEGF by BMSCs. These results suggest that the JLSV–BMSC microtissue enhances the paracrine function of BMSCs, which likely contributes to improved therapeutic outcomes by promoting the secretion of factors critical for cell survival, angiogenesis, and tissue repair.

To further investigate the effects of enhanced paracrine activity on cardiomyocyte AC16 cells, human umbilical vein endothelial cells (HUVECs), and cardiac fibroblasts under ischemic and hypoxic conditions, we used an indirect Transwell co‐culture system (Figure 5B). BMSCs or JLSV–BMSC microtissues were co‐cultured with cardiomyocytes, HUVECs, and cardiac fibroblasts in a Transwell system. The protective effects of BMSCs or JLSV–BMSC microtissues on cardiomyocytes were evaluated by analyzing apoptotic markers (Bax and Bcl2) using western blotting and live/dead cell staining. Treatment with JLSV–BMSC microtissues significantly protected cardiomyocytes from OGD‐induced apoptosis, as evidenced by a significant decrease in the Bax/Bcl2 ratio than BMSC treatment alone (Figure 5E, F). Live/dead staining confirmed these results, showing a higher survival rate of cardiomyocytes treated with JLSV‐BMSC microtissues under OGD conditions than in those treated with BMSCs alone (Figure 5C, D; Figure S10, Supporting Information). These results indicate that JLSV–BMSC microtissue provides better protection against OGD‐induced cardiomyocyte apoptosis than treatment with BMSCs alone.

Tube formation assays were performed to evaluate the angiogenic potential of HUVECs. JLSV–BMSC microtissues significantly promoted tube formation by HUVECs under OGD conditions, with better results than the BMSC treatment group (Figure 5G, H). Western blot analysis of apoptosis‐related markers in HUVECs showed that the JLSV–BMSC microtissue protected against OGD‐induced apoptosis of HUVECs, with a stronger effect than that of BMSC treatment alone (Figure S9A, B, Supporting Information). In addition, Ki67 immunocytochemical staining showed a significant reduction in endothelial cell proliferation under OGD conditions than that under normal conditions. Both BMSC and JLSV–BMSC microtissue treatments reversed this effect; however, JLSV–BMSC microtissue showed a more pronounced increase in endothelial cell proliferation than BMSC alone (Figure S9D, C, Supporting Information). The Transwell migration assay showed that HUVEC migration was significantly reduced under OGD conditions than that under normal conditions. While both BMSC and JLSV–BMSC microtissue treatments reversed this reduction, JLSV–BMSC microtissues significantly promoted endothelial cell migration to a greater extent than BMSC treatment (Figure S9F, E, Supporting Information). These results suggest that JLSV–BMSC microtissues are more effective than BMSC in promoting angiogenesis, reducing apoptosis, and increasing the proliferation and migration of endothelial cells under OGD conditions.

Western blot analysis was used to investigate the expression of proteins, including the fibrotic markers type I and type III collagen, and myofibroblast markers alpha‐smooth muscle actin (α‐SMA) and vimentin, in cardiac fibroblasts under OGD conditions. Both BMSC and JLSV–BMSC microtissue reduced the levels of these markers. However, JLSV–BMSC microtissue more significantly reduced fibrosis and myofibroblast markers than BMSC (Figure 5I,J). Immunocytochemical staining of α‐SMA and vimentin as well as morphological assessment of cardiac fibroblasts, showed that JLSV–BMSC microtissue more effectively reversed fibroblast transition to myofibroblastic phenotype, characterized by elongated and contractile morphology, than BMSCs (Figure S9I, Supporting Information). The reduced fluorescence intensity of α‐SMA and vimentin, as well as the western blot analysis described above, confirmed that the JLSV–BMSC microtissue significantly attenuated the transition of fibroblasts to myofibroblast phenotype.

Under normal conditions, the co‐culture of BMSCs or JLSV–BMSC microtissue with cardiac fibroblasts, HUVECs, and cardiomyocytes showed no statistically significant differences in the relevant phenotypes than the vehicle control group, further confirming the safety of the JLSV–BMSC microtissue.

In summary, the JLSV–BMSC microtissue enhanced the paracrine activity of BMSCs, increasing the secretion of key pro‐survival and angiogenic factors. This enhanced paracrine activity protects cardiomyocytes from apoptosis, promotes HUVEC proliferation and angiogenesis, and attenuates collagen production in cardiac fibroblasts under OGD conditions. The ability of the JLSV–BMSC microtissue to improve cell survival, reduce fibrosis, and stimulate vascular growth underscores its potential as a valuable tool for regenerative therapies for ischemic tissues.

### 2.7 JLSV–BMSC Microtissue Improves Retention and Differentiation of BMSCs into Cardiomyocytes and Endothelial Cells In Vivo

To determine the optimal dosage of BMSCs in JLSV–BMSC microtissues for use in animal models, we first reviewed existing studies to identify effective and safe dosages specifically for treating MI. These studies generally recommend using 2 × 10^5^ to 1 × 10^6^ cells for direct myocardial injection in mouse models.^[^ ^7^, ^37^ ^]^ Based on our in vitro experiments, we considered the role of JLSV as a biomimetic extracellular scaffold that helps BMSCs survive in the ischemic and hypoxic environment of MI. To explore whether JLSV nanoscaffolds can achieve improved therapeutic effects using fewer BMSCs, we created mouse cohorts with JLSV microtissues containing varying numbers of BMSCs and conducted in vivo experiments at low (4 × 10^4^), medium (2 × 10^5^), and high (1 × 10^6^) doses. Sham‐operated and untreated MI mice were used as controls to evaluate the therapeutic outcomes. We used Masson's trichrome, α‐SMA and von Willebrand Factor (vWF) immunofluorescence, and TUNEL staining to analyze changes in myocardial fibrosis, neovascularization in the infarct area, and cardiomyocyte apoptosis, respectively (Figure S11, Supporting Information). The results showed that mice receiving low‐dose microtissue treatment showed significantly less improvement than the medium‐ and high‐dose groups. Notably, the medium BMSC dose (2 × 10^5^) resulted in a similar cardiac recovery as the high dose (1 × 10^6^), indicating a dose‐dependent threshold, beyond which additional cells do not provide additional therapeutic benefits. This could be owing to the unique ECM properties of JLSV, which enhance BMSC activity and function and reduce the need for higher doses to achieve improved therapeutic effects. Based on these results, we selected a medium dose (2 × 10^5^) for subsequent experiments. To comprehensively evaluate the retention and differentiation potential of transplanted BMSCs at the infarct sites in vivo, we used two different strategies to precisely track the transplanted cells. First, the transplanted BMSCs were labeled with the red fluorescent dye PKH26, and fluorescence imaging was performed using the IVIS Kinetic System, which provides a comprehensive overview of cell survival over time. Second, the cells were genetically engineered to express red fluorescent protein (RFP), which allowed the accurate localization and assessment of cell behavior in the infarct region. This approach enabled reliable monitoring of the fate of the injected stem cells over the study course. To further assess stem cell differentiation, tissue sections were analyzed with immunofluorescence staining in combination with colocalization of the transfected RFP‐positive BMSCs to assess stem cell differentiation in vivo.

**Figure 6:**
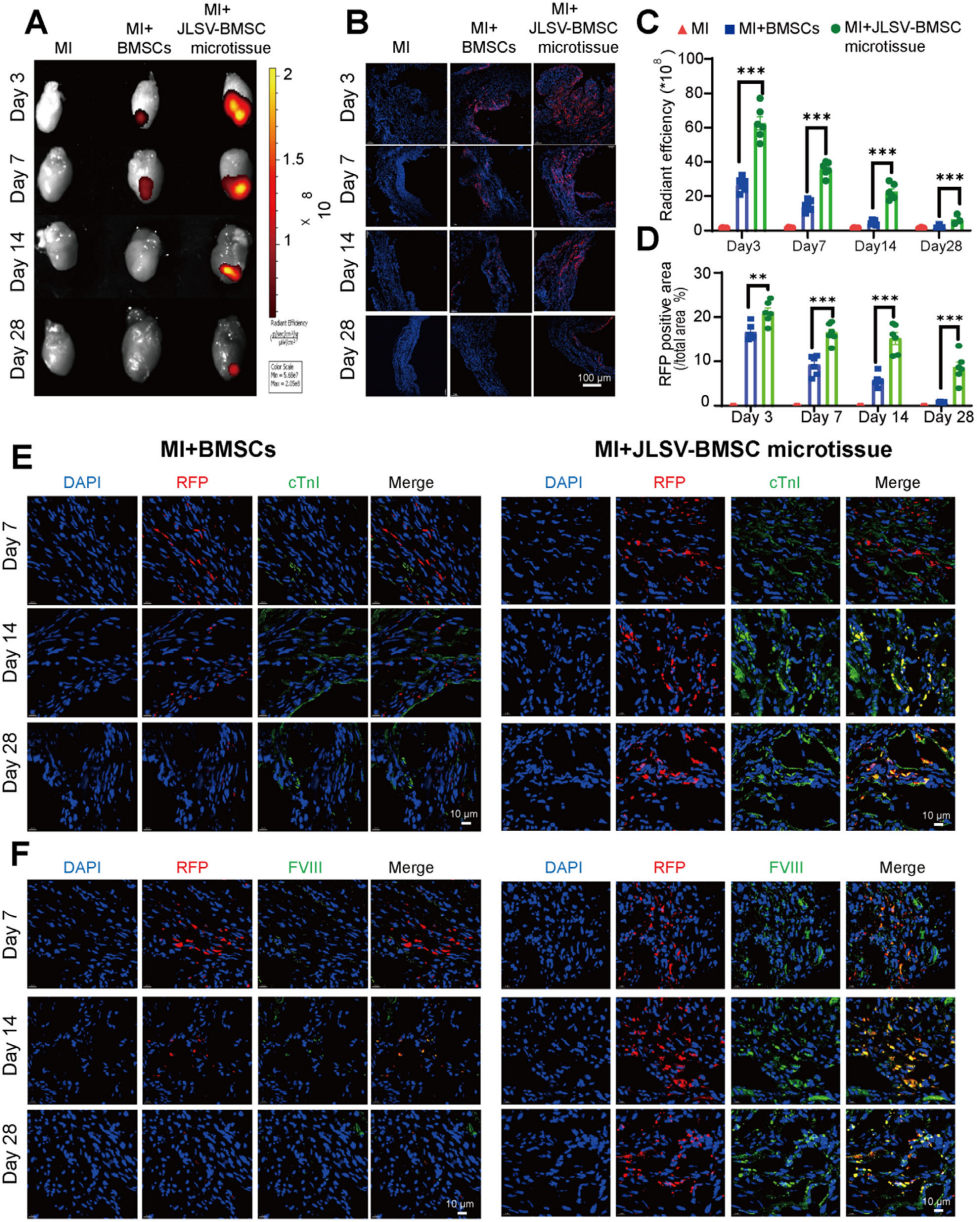
A dual biomimetic extracellular matrix scaffold (JLSV) consisting of Janus Basic Nanotubes, laminin, stromal‐derived factor‐1 alpha, and vascular endothelial growth factor for bone marrow‐derived mesenchymal stem cell (BMSC) delivery (JLSV–BMSC microtissue) improves BMSC retention and differentiation into cardiomyocytes and endothelial cells in vivo. A) The quantity of intramyocardial transplanted BMSCs or JLSV–BMSC microtissue was indicated by the radiant efficiency of fluorescence intensity in PKH26 signal images. Representative images are shown. B) Confocal microscopy images showing red fluorescence protein (RFP) ‐expressing BMSCs (red) in frozen heart slices 3, 7, 14, and 28 days after transplantation, with nuclei counterstained with DAPI (blue). Representative images are shown. C) Quantitative analysis of the radiant efficiency of fluorescence intensity over time was used to assess cell retention at the infarct site in excised hearts. Data are expressed as the mean ± standard error of the mean from six mice per group. ^*^ *p* < 0.05, ^**^ *p* < 0.01, ^***^ *p* < 0.001. D) To evaluate cell retention, a quantitative analysis was performed by calculating the RFP/DAPI ratio in heart slices 3, 7, 14, and 28 days after transplantation. Data are expressed as the mean ± standard error of the mean from six mice per group. ^*^ *p* < 0.05, ^**^ *p* < 0.01, ^***^ *p* < 0.001. E). Confocal immunofluorescence images of frozen heart sections showing RFP‐expressing BMSCs (red) colocalized with the cardiomyocyte‐specific marker cTnI (green) 7, 14, and 28 days after transplantation, with nuclei counterstained with DAPI (blue). Representative images are shown. F) Confocal immunofluorescence images of frozen heart sections showing RFP‐expressing BMSCs (red) colocalized with the endothelial cell‐specific marker FVIII (green) 7, 14, and 28 days after transplantation, with nuclei counterstained with DAPI (blue). Representative images are shown.

In the first strategy, in vivo imaging was performed at different time points (3, 7, 14, and 28 days) after transplantation to monitor the retention of PKH26‐labeled BMSCs in the infarcted myocardial region. In vivo imaging results showed that the imaging signals were consistently stronger in the JLSV–BMSC microtissue group than in the BMSC group at all time points, indicating prolonged cell retention. Although in vivo imaging signals were not observed in either group on day 28, the JLSV–BMSC microtissue group still showed detectable fluorescence signals on day 14 after MI, whereas the BMSC group showed no detectable fluorescence signals at this time point (Figure S12, Supporting Information). To obtain a precise assessment of cell retention, *ex vivo* imaging was performed in which the hearts were sampled at specific time intervals. The *ex vivo* results showed that the fluorescence intensity was consistently higher in the JLSV–BMSC microtissue group than in the BMSC group at all time points, indicating prolonged retention. While no fluorescence signals were detected in the BMSC group after day 14, the JLSV–BMSC microtissue group showed detectable fluorescence signals on day 28 (**Figure** **6**A,C).

For the second strategy, the hearts of mice injected with RFP‐expressing BMSCs were harvested at specific time intervals (days 3, 7, 14, and 28), and frozen sections were prepared to quantify and visualize the retention of transplanted cells. The harvested hearts were cut into frozen 5‐µm slices, which were analyzed by confocal microscopy. A detailed analysis of these slices revealed a significantly higher number of RFP‐expressing BMSCs in the JLSV–BMSC microtissue group than in the BMSC group at all observed time points. Quantitative evaluation of the ratio of RFP to DAPI (nuclear staining) confirmed that the JLSV–BMSC microtissue group had a significantly higher retention of transplanted cells within the infarct zone, as evidenced by a higher RFP/DAPI ratio (Figure 6B,D). This result suggests that JLSV–BMSC microtissue plays a crucial role in maintaining BMSC retention at the site of infarct injury.

To assess whether the retained BMSCs differentiated into cardiomyocytes or endothelial lineages, mice from the two groups were collected on days 3, 7, 14, and 28 and processed for immunofluorescence staining to detect specific cardiac and endothelial markers. Confocal microscopy was used to investigate the colocalization of RFP‐expressing BMSCs with these markers. For myocardial differentiation, we analyzed cTnI and GATA4, which are key markers of cardiomyocyte differentiation. We focused on FVIII and CD31, which are important markers of vascular development and endothelial differentiation.

Colocalization analysis revealed a significantly higher number of RFP‐positive cells and a clear differentiation trend in the JLSV–BMSC microtissue group. During the 4‐week observation period, the JLSV–BMSC microtissue group exhibited earlier differentiation, starting as early as day 7, and showing more development toward cardiomyocytes and endothelial cells (Figure 6E,F, Figures S13–S16, Supporting Information). These results suggest that JLSV–BMSC microtissue not only improves cell maintenance but also actively promotes BMSC differentiation into important cell types required for cardiac repair.

In summary, the JLSV–BMSC microtissues significantly improved the transplanted BMSC retention and differentiation. This effect can be attributed to the mechanical and biological properties of the JLSV–BMSC microtissue, which create an optimal environment for the long‐term survival and differentiation of BMSCs. These dual benefits underscore the potential of the JLSV–BMSC microtissue as a powerful tool for enhancing stem cell‐based therapies targeting damaged myocardial tissue repair.

### 2.8 JLSV–BMSC Microtissue Improves Cardiac Function, Promotes Angiogenesis, and Protects the Myocardium from Apoptosis Post‐MI

We investigated the effects of JLSV–BMSC microtissue on cardiac function recovery in mice with MI. Specifically, we aimed to determine whether treatment with JLSV–BMSC microtissue led to better cardiac function recovery than treatment with JLSV or BMSCs alone. Additionally, we evaluated the safety of BMSCs, JLSV, and JLSV–BMSC microtissues. Animals were divided into two experimental cohorts.

The first cohort consisted of six groups: Sham, Sham+BMSCs, Sham+JLSV–BMSC microtissues, MI+saline, MI+BMSCs, and MI+JLSV–BMSC microtissues. The second cohort comprised six groups: Sham, Sham+JLSV, Sham+JLSV–BMSC microtissue, MI+saline, MI+JLSV, and MI+JLSV–BMSC microtissue. In both cohorts, transthoracic echocardiography was used to monitor the changes in cardiac function after infarction.

**Figure 7:**
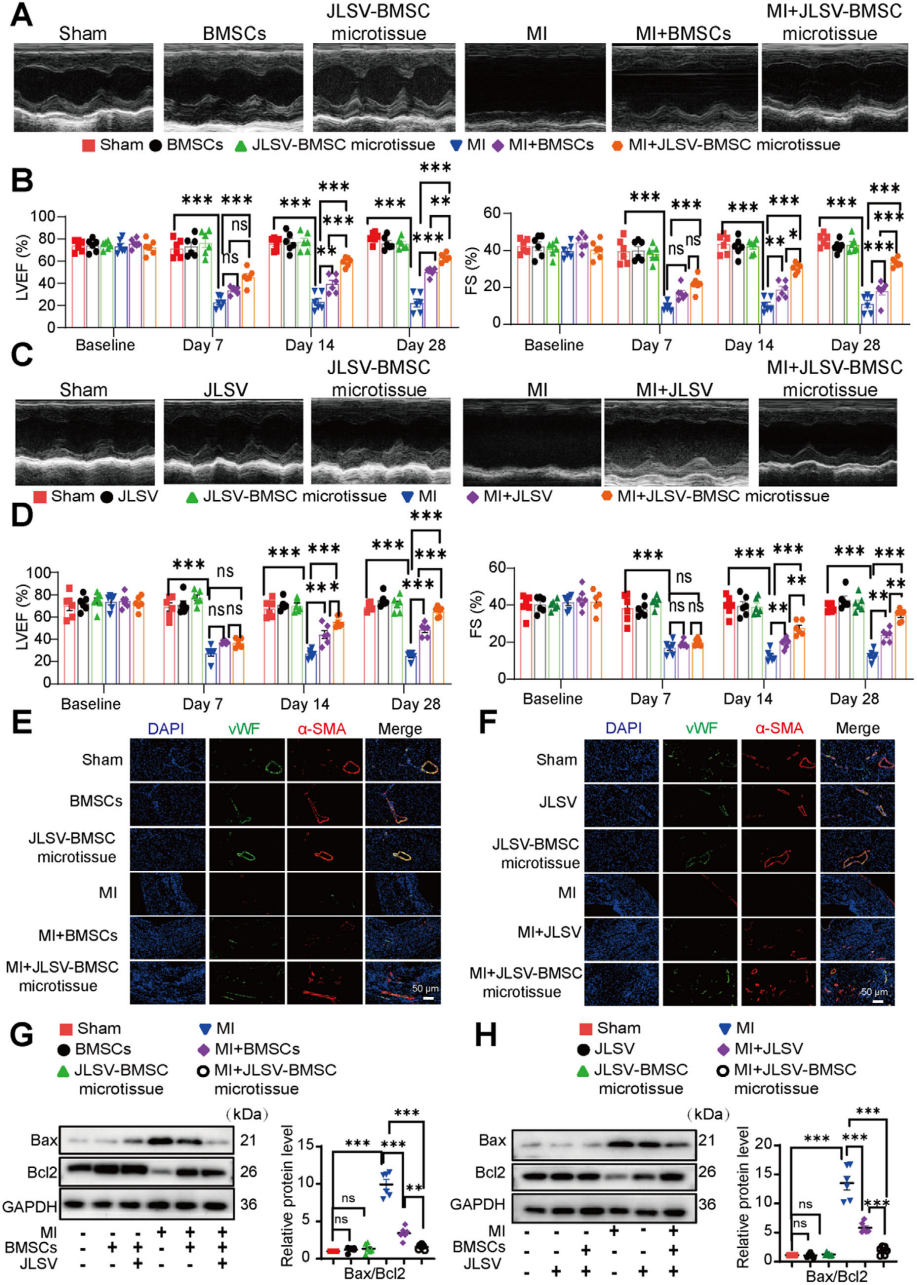
A dual biomimetic extracellular matrix scaffold (JLSV) consisting of Janus Basic Nanotubes, laminin, stromal‐derived factor‐1 alpha, and vascular endothelial growth factor for bone marrow‐derived mesenchymal stem cell (BMSC) delivery (JLSV–BMSC microtissue) improves cardiac function, promotes angiogenesis, and protects the myocardium from apoptosis post‐myocardial infarction (MI). A) Representative echocardiographic images on day 28 from Sham, Sham+BMSCs, Sham+JLSV–BMSC microtissue, MI+saline, MI+BMSCs, and MI+JLSV–BMSC microtissue groups. B) Quantitative analysis of left ventricular ejection fraction (LVEF) and fractional shortening (FS) at baseline, day 7, day 14, and day 28. Data are expressed as the mean ± standard error of the mean from six mice per group. ^*^ *p* < 0.05, ^**^ *p* < 0.01, ^***^ *p* < 0.001. C) Representative echocardiographic images at day 28 from Sham, Sham+JLSV, Sham+JLSV–BMSC microtissue, MI+saline, MI+JLSV, and MI+JLSV–BMSC microtissue groups. D) Quantitative analysis of LVEF and FS at baseline, day 7, day 14, and day 28. Data are expressed as the mean ± standard error of the mean from six mice per group. ^*^ *p* < 0.05, ^**^ *p* < 0.01, ^***^ *p* < 0.001. E) Representative immunofluorescence images of alpha‐smooth muscle actin (α‐SMA) ‐positive and von Willebrand factor (vWF) ‐positive blood vessels in the infarct region on day 28 after implantation. Images are shown in the following groups: Sham, Sham+BMSCs, Sham+JLSV–BMSC microtissue, MI+saline, MI+BMSCs, and MI+JLSV–BMSC microtissue. F) Representative immunofluorescence images of α‐SMA‐positive and vWF‐positive blood vessels in the infarct region on day 28 after implantation. Images are shown in the Sham, Sham+JLSV, Sham+JLSV–BMSC microtissue, MI+saline, MI+JLSV, and MI+JLSV–BMSC microtissue groups. G) Representative western blot images for Bax and Bcl2 in tissue from Sham, Sham+BMSC, Sham+JLSV–BMSC microtissue, MI+saline, MI+BMSCs, and MI+JLSV–BMSC microtissue groups. Quantitative analysis of Bax/Bcl2 expression is expressed as the mean ± standard error of the mean from six mice per group. ^*^ *p* < 0.05, ^**^ *p* < 0.01, ^***^ *p* < 0.001. H) Representative western blot images for Bax and Bcl2 in tissue from Sham, Sham+JLSV, Sham+JLSV–BMSC microtissue, MI+saline, MI+JLSV, and MI+JLSV–BMSC microtissue groups. Quantitative analysis of Bax/Bcl2 is expressed as the mean ± standard error of the mean from six mice per group. ^*^ *p* < 0.05, ^**^ *p* < 0.01, ^***^ *p* < 0.001.

In the first cohort, we observed a significant decrease in myocardial contractility 4 weeks after infarction in the MI+saline group, as evidenced by a decrease in the left ventricular ejection fraction (LVEF) and fractional shortening (FS), indicating negative remodeling. Both BMSC and JLSV–BMSC microtissue groups showed improved cardiac function with partial recovery of LVEF and FS than the MI+saline group. However, the JLSV–BMSC microtissue group showed higher LVEF and FS values than the BMSC group, suggesting that the JLSV–BMSC microtissue group showed improved contractility and effective prevention of left ventricular dilation (**Figure** **7**A,B). In the second cohort, the MI+saline group exhibited significant cardiac dysfunction, characterized by decreased LVEF and FS, indicating unfavorable remodeling. Both the JLSV and JLSV–BMSC microtissue groups showed moderate improvements in cardiac function than the MI+saline group. However, the JLSV–BMSC microtissue group showed more significant improvement than the JLSV group (Figure 7C,D). In both animal cohorts, the cardiac function was not significantly different between the Sham, Sham+BMSCs, Sham+JLSV, and Sham+JLSV–BMSC microtissue groups, confirming the in vivo safety of the microtissue grafts.

In addition to evaluating improvements in cardiac function, we evaluated the ability of these treatments to promote angiogenesis and reduce myocardial apoptosis, as these processes are critical for effective cardiac repair. Immunofluorescence staining for α‐SMA and vWF was performed to assess angiogenesis. Apoptosis‐related markers and TUNEL staining were performed to assess myocardial apoptosis.

In the first cohort, limited angiogenesis was observed in the MI+saline group, with sparse α‐SMA‐positive and vWF‐positive blood vessels. Both BMSC and JLSV–BMSC microtissue groups showed increased angiogenesis. However, the JLSV–BMSC microtissue group showed a significantly greater increase in α‐SMA‐positive and vWF‐positive blood vessel density than the BMSC group (Figure 7E). In addition, widespread apoptosis of myocardial tissue was detected in the MI+saline group, as characterized by western blot analysis, which revealed a pronounced increase in Bax and a decrease in Bcl2, and high levels of apoptotic cells, as shown by TUNEL staining. Both BMSC and JLSV–BMSC microtissue groups showed less apoptosis than the MI+saline group. However, the JLSV–BMSC microtissue group showed significantly lower levels of apoptosis than the BMSC group (Figure 7G), and TUNEL staining revealed similar results (Figure S17A, Supporting Information). In the second cohort, the MI+saline group showed limited angiogenesis with sparse α‐SMA‐positive and vWF‐positive blood vessels and widespread apoptosis indicated by increased Bax levels, decreased Bcl2 levels, and TUNEL staining. Both JLSV and JLSV–BMSC microtissue groups exhibited reduced apoptosis and improved angiogenesis than the MI+saline group. Notably, the JLSV–BMSC microtissue group showed a significantly higher density of α‐SMA‐positive and vWF‐positive blood vessels (Figure 7F), lower Bax levels, and higher Bcl2 levels than the JLSV group, and TUNEL staining showed that fewer apoptotic cells were present than in the JLSV group, highlighting their superior therapeutic efficacy (Figure 7H and Figure S17B, Supporting Information**)**. In both animal cohorts, no significant differences were observed between the Sham, Sham+BMSCs, Sham+JLSV, and Sham+JLSV–BMSC microtissue groups in terms of angiogenesis or apoptosis‐related markers, as determined by western blot analysis of tissue proteins, confirming the in vivo safety of the microtissue.

These results clearly show that JLSV–BMSC microtissue effectively promotes the repair of the infarcted region. Cardiac ultrasound showed that the JLSV–BMSC microtissue significantly improved cardiac function than BMSC or JLSV. Histological analyses and western blotting consistently showed that the JLSV–BMSC microtissue effectively promotes angiogenesis and reduces apoptosis, thereby improving myocardial repair at the infarct site. These results underscore the superior repair ability of JLSV–BMSC microtissue in MI and emphasize its overall therapeutic efficacy compared to BMSCs or JLSV.

### 2.9 JLSV–BMSC Microtissue Alleviates Cardiac Fibrosis in Mice with MI

We investigated the therapeutic efficacy of the JLSV–BMSC microtissue in reducing myocardial fibrosis compared to that of BMSCs or JLSV, while confirming their safety profile. Two independent animal cohorts were used to ensure a comprehensive evaluation. The first cohort consisted of six groups: Sham, Sham+BMSCs, Sham+JLSV–BMSC microtissues, MI+saline, MI+BMSCs, and MI+JLSV–BMSC microtissues. The second cohort comprised Sham, Sham+JLSV, Sham+JLSV–BMSC microtissues, MI+saline, MI+JLSV, and MI+JLSV–BMSC microtissues.

**Figure 8:**
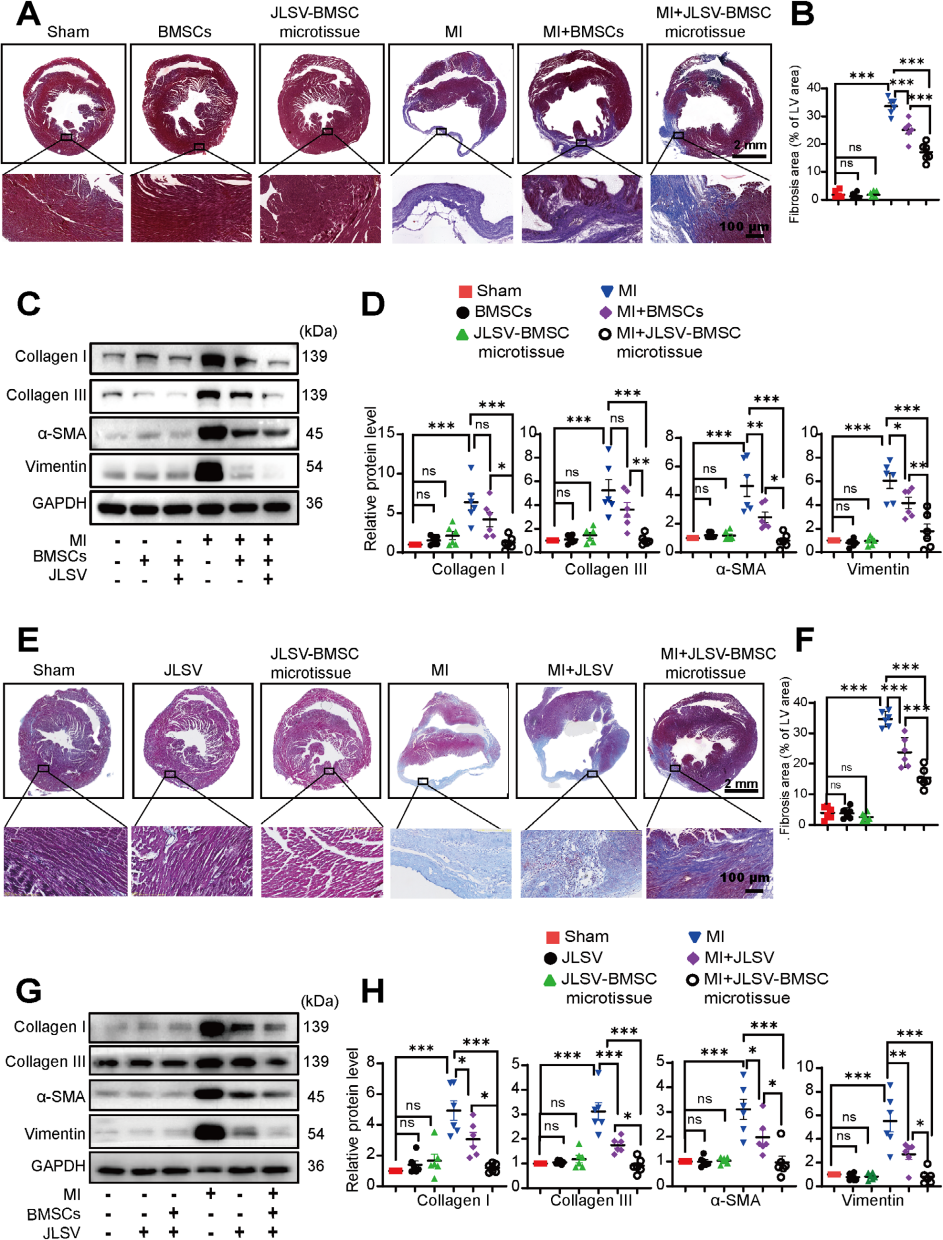
A dual biomimetic extracellular matrix scaffold (JLSV) consisting of Janus Basic Nanotubes, laminin, stromal‐derived factor‐1 alpha, and vascular endothelial growth factor for bone marrow‐derived mesenchymal stem cell (BMSC) delivery (JLSV–BMSC microtissue) alleviates cardiac fibrosis in mice with myocardial infarction (MI). A) Masson's trichrome staining of the whole heart in the following groups: Sham, Sham+BMSCs, Sham+JLSV–BMSC microtissue, MI+saline, MI+BMSCs, and MI+JLSV–BMSC microtissue. Representative images of the infarct zone on day 28 post‐MI. B) Quantitative analysis of the fibrosis area using Masson's trichrome staining. Data are expressed as mean ± standard error of the mean from six mice per group. ^*^ *p* < 0.05, ^**^ *p* < 0.01, ^***^ *p* < 0.001. C) Representative western blot images showing collagen I, collagen III, alpha‐smooth muscle actin (α‐SMA), and vimentin expression in tissue from the following groups: Sham, Sham+BMSCs, Sham+JLSV–BMSC microtissue, MI+saline, MI+BMSCs, and MI+JLSV–BMSC microtissue. D) Quantitative analysis of collagen I, collagen III, α‐SMA, and vimentin expression. Data are expressed as mean ± standard error of the mean from six mice per group. ^*^ *p* < 0.05, ^**^ *p* < 0.01, ^***^ *p* < 0.001. E) Masson's trichrome staining of the whole heart in the following groups: Sham, Sham+JLSV, Sham+JLSV–BMSC microtissue, MI + saline, MI + JLSV, and MI + JLSV–BMSC microtissue. Representative images of the infarct zone on day 28 post‐MI. F) Quantitative analysis of the fibrosis area using Masson's trichrome staining. Data are expressed as mean ± standard error of the mean from six mice per group. ^*^ *p* < 0.05, ^**^ *p* < 0.01, ^***^ *p* < 0.001. G) Representative western blot images showing the expression of collagen I, collagen III, α‐SMA, and vimentin in tissue from the following groups: Sham, Sham+BMSCs, Sham+JLSV–BMSC microtissue, MI+saline, MI+BMSCs, and MI+JLSV–BMSC microtissue. H) Quantitative analysis of collagen I, collagen III, α‐SMA, and vimentin expression. Data are expressed as mean ± standard error of the mean from six mice per group. ^*^ *p* < 0.05, ^**^ *p* < 0.01, ^***^ *p* < 0.001.

In the first cohort, Masson's trichrome and Sirius red staining showed that the MI+saline group had extensive myocardial fibrosis, characterized by dense collagen deposits. Western blot analysis confirmed elevated collagen I, collagen III, α‐SMA, and vimentin levels, indicating severe fibrotic remodeling associated with fibroblast activation into myofibroblast and collagen deposits. Fibrosis was partially reduced in both the BMSC and JLSV–BMSC microtissue groups. Masson's trichrome and Sirius red staining indicated smaller fibrotic areas, and western blot analysis showed decreased expression of fibrosis markers collagen I and collagen III, and myofibroblast markers vimentin and α‐SMA than in the MI+saline group. Notably, the JLSV–BMSC microtissue group showed a greater reduction in myocardial fibrosis than the BMSC group. Masson's trichrome and Sirius red staining showed a marked decrease in the fibrotic area within the infarct zone (**Figure** **8**A,B, Figure S17C, Supporting Information), and western blot analysis also confirmed lower collagen I, collagen III, α‐SMA, and vimentin expression levels in the JLSV–BMSC microtissue group than in the BMSC group (Figure 8C,D).

In the second cohort, the MI+saline group exhibited widespread myocardial fibrosis, as evidenced by dense collagen deposits in the infarct and peri‐infarct areas. Western blot analysis revealed elevated collagen I, collagen III, α‐SMA, and vimentin levels, confirming severe fibrotic remodeling. Both JLSV and JLSV–BMSC microtissue groups showed a reduction in fibrosis, as reflected by smaller fibrotic areas in Masson's trichrome and Sirius red staining and lower expression of fibrosis‐related markers in western blot analysis. Importantly, the JLSV–BMSC microtissue group showed a greater reduction in the fibrotic area (Figure 8E,F, Figure S17D, Supporting Information) and expression of fibrosis markers (Figure 8G,H) than the JLSV group, highlighting its superior therapeutic efficacy.

In both animal cohorts, no significant differences in fibrosis‐related markers or staining were observed between the Sham, Sham+BMSCs, Sham+JLSV, and Sham+JLSV–BMSC microtissue groups, confirming the in vivo safety of the microtissue in noninjured cardiac tissue.

These results emphasize the superior therapeutic potential of the JLSV–BMSC microtissue in reducing myocardial fibrosis, outperforming both BMSCs and JLSV nanoscaffolds independently.

## 3 Discussion

In this study, we developed a novel biomimetic microtissue system called JLSV–BMSC microtissue. This microtissue integrates a flexible injectable nanoscaffold with bioactive molecules to enhance BMSC survival, proliferation, and differentiation, particularly in the harsh microenvironment of the infarcted myocardium. The microtissue was designed to mimic the ECM, providing essential physical support for BMSCs while anchoring multiple cytokines to deliver critical biological support.

Current therapeutic strategies for reperfusing occluded vessels in patients with acute MI mainly include intravenous pharmacological thrombolysis, percutaneous coronary intervention (PCI), and coronary artery bypass grafting (CABG). Our research offers a complementary biological approach that can potentially reduce residual risks. The myocardial injection method is difficult to implement in PCI, but feasible in the clinical setting of CABG surgery. Notably, some intramyocardial biomaterials, such as cell‐laden collagen scaffolds, are safe and feasible in clinical trials.^[^ ^38^ ^]^ Our strategy could be particularly beneficial for patients who may not fully benefit from traditional methods owing to limitations, such as advanced disease states or specific contraindications.

Recent studies have shown that the myocardial microenvironment is primarily composed of biological factors produced by non‐cardiomyocytes.^[^ ^39^ ^]^ The sudden and widespread cardiomyocyte loss post‐MI triggers acute inflammation, leading to muscle fiber disorganization and destruction.^[^ ^22^, ^40^ ^]^ Vascular occlusion further leads to local myocardial ischemia and hypoxia ^[^ ^41^ ^]^ while the ECM undergoes progressive degradation, resulting in structural integrity and contractile function loss.^[^ ^42^ ^]^ These pathological changes significantly impair BMSC survival and migration.^[^ ^43^ ^]^ The BMSCs can be induced in vitro to differentiate into cardiomyocyte‐like cells with sarcomere structures capable of spontaneous contraction.^[^ ^44^ ^]^ Moreover, the efficiency of BMSC differentiation into cardiomyocytes can be improved by combining different chemical compounds. However, the BMSCs transplanted directly into the myocardium rarely differentiate into cardiomyocytes. This limitation is likely owing to the lack of key signaling cues and a supportive microenvironment.^[^ ^45^ ^]^ Consequently, the efficacy of BMSC‐based therapies is highly dependent on the recipient myocardial microenvironment quality. Improving this microenvironment is crucial for unleashing the full regenerative potential of BMSCs.

Synthetic DNA base pairs formed JBNTs that mimicked the natural helical nanostructure and surface chemistry of collagen in the ECM of cardiomyocytes.^[^ ^23a,b^ ^]^ Integrating bioactive proteins, including SDF‐1α, VEGF, and laminin, creates a favorable biological environment that promotes BMSC activities critical for myocardial repair. Thus, the JLSV–BMSC microtissue structurally mimics the native ECM, provides essential physical support for cell attachment ^[^ ^46^ ^]^, and facilitates repair factor release and BMSC differentiation stimuli.^[^ ^23b,c^ ^]^

In vitro experiments confirmed the efficacy of the microtissue in promoting proliferation, migration, and differentiation, while protecting against BMSC apoptosis under OGD conditions. Comprehensive safety was demonstrated and its biocompatibility was confirmed. Furthermore, we investigated the protective effect of each component of JLSV on BMSCs and found that integrated JLSV as a coherent system provided the most effective protection for BMSCs. These experiments showed that JLSV–BMSC microtissues enhance paracrine signaling effects than BMSCs alone. Specifically, the microtissue enhanced cardiomyocyte and HUVEC viability by decreasing their apoptosis, promoting HUVEC proliferation and tube formation, and attenuating the transition of fibroblasts to the myofibroblast phenotype, while decreasing collagen secretion. This demonstrates the ability of the JLSV–BMSC microtissue to modulate cellular responses under hypoxic stress.

In addition to improving cell viability in vitro, in vivo experiments have demonstrated that the microtissue plays an important role in improving BMSC retention in the infarcted myocardium. Imaging and histological analyses confirmed that the JLSV–BMSC microtissue remained at the infarct site longer than the BMSCs administered alone. These two approaches allowed precise cell retention and distribution monitoring, further confirming the role of microtissue in maintaining the presence of BMSCs over extended periods. Therefore, the safety and functional sustainability of the JLSV as a therapeutic biomaterial should be confirmed. We conducted both in vivo and *ex vivo* real‐time optical imaging by tagging JLSV with the fluorescent dye Cy7 to monitor their presence over 28 days. The imaging results showed that the scaffold was primarily localized in the heart, indicating effective retention, where it can facilitate myocardial repair. The fluorescence intensity gradually decreased and was nearly undetectable after 28 days. This timeline ensures that the scaffold supports the essential phases of cardiac remodeling without long‐term accumulation. Moreover, blood tests showed no significant differences in liver and kidney function and routine blood tests across all groups and time points, confirming the biocompatibility and systemic safety of the JLSV scaffold. The ability of the microtissue to mimic ECM‐like properties also facilitates targeted BMSC differentiation into cardiomyocytes and endothelial cells,^[^ ^47^ ^]^ as evidenced by the increased expression of differentiation markers, including GATA4, cTnI, FVIII, and CD31, in immunofluorescence colocalization analysis. To explore the mechanisms of JLSV‐induced BMSC differentiation into cardiomyocytes, we conducted a transcriptomic analysis. Adding JLSV to BMSCs upregulated cell differentiation‐associated genes and activated some key signaling pathways, particularly the PI3K/AKT pathway. However, owing to the complex in vivo environment, the detailed mechanisms by which JLSV induces BMSC differentiation require further investigation. Additionally, the microtissue reduced apoptosis and fibrosis while promoting angiogenesis, contributing to significant improvements in cardiac function, as evidenced by improved LVEF and FS in the treated animals. Finally, this study demonstrated the safety profile of the JLSV–BMSC microtissue. Echocardiography and histological examination revealed no adverse effects on the myocardium. The delivered JLSV–BMSC microtissue exhibited high biocompatibility with no evidence of fibrosis or other pathological changes, supporting its broad applicability in cardiac regeneration.

In conclusion, the JLSV–BMSC microtissue represents a promising therapeutic platform for myocardial repair, addressing long‐standing challenges associated with cell maintenance, differentiation, and integration in unfavorable myocardial microenvironments. These results highlight the role of this microtissue as a physical support and a sustainable source of bioactive proteins crucial for long‐term repair, demonstrating its ability to effectively mimic the myocardium microenvironment in terms of both physical structure and biological function. Furthermore, the synergistic effect of structural support and biological cues in the microtissue design was crucial for BMSC differentiation, emphasizing the importance of composite microtissues. The ability of microtissues to facilitate precise cellular differentiation has significant therapeutic potential and enables targeted and effective tissue regeneration to restore myocardial function after infarction.

Despite these promising results, this study has several limitations. First, while our results show significant therapeutic benefits, the underlying mechanisms by which JLSV–BMSC microtissue repairs damaged myocardium are not yet fully understood. For example, the specific signaling pathways and cellular interactions facilitated by microtissues remain to be fully elucidated. Future research should aim to uncover these mechanistic pathways to deepen our understanding of how biomimetic microtissues support myocardial repair at the molecular level. Although we have not yet achieved the induction of cardiomyocyte beating in vitro, after injecting the microtissues in vivo, the stem cells within these microtissues survived for over 28 days. This extended survival offers stem cells the opportunity to differentiate in a true in vivo environment. While we do not yet have definitive evidence that stem cells in microtissues differentiate into fully mature cardiomyocytes, the current results indicate that these microtissues exert significant efficacy in treating MI. Additionally, cardiac tissue section staining revealed the differentiation of transplanted BMSCs into the cardiomyocyte lineage, suggesting the potential of stem cells to differentiate into mature cardiomyocytes. In future studies, we will explore this highly promising aspect in detail. Although this study focused on structural and functional recovery, long‐term studies are required to evaluate the sustained effects and potential adjustments to the scaffold composition. Such studies would provide further insights into the applicability and efficacy of scaffolds for clinical use. Addressing these limitations could enhance the therapeutic effect of JLSV–BMSC microtissues and expand their utility for a broad range of applications in regenerative medicine targeting ischemic tissues.

## 4 Experimental Section

### Zeta Potential Test

Three groups of samples were prepared. For the laminin group, 40 µL 160 ng mL^−1^ laminin (7340‐A4, R&D) was added to 10 µL deionized water to obtain a 160 ng mL^−1^ laminin solution. For the laminin/VEGF/SDF‐1α (LSV) group, 10 µL 40 ng mL^−1^ VEGF (100‐20, Pepro Tech), 2.5 µL 200 ng mL^−1^ SDF‐1α (300‐28A, Pepro Tech), 4 µL 2 µg mL^−1^ laminin were added to 23.5 µL deionized water and pipetted several times. The final laminin, SDF‐1α, and VEGF concentrations in the LSV group were 160, 10, and 40 ng mL^−1^, respectively. For the JLSV group, 4 µL 2 µg mL^−1^ laminin, 1 µL 2 µg mL^−1^ VEGF, 0.25 µL 2 µg mL^−1^ SDF‐1α, and 1 µL 1 mg mL^−1^ JBNTs were added to 43.75 µL deionized water and pipetted several times. The final JBNT, laminin, SDF‐1α, and VEGF concentrations in the JLSV group were 20 µg mL^−1^, 160 ng mL^−1^, 10 ng mL^−1^, and 40 ng mL^−1^, respectively. The zeta potentials of the three groups were measured using a Zetasizer Nano ZS (Malvern Panalytical).

### JLSV and JLSV‐BMSC Microtissue Fabrication

The G^C and A^T units of JBNTs were synthesized as described previously ^[^ ^23b^ ^]^, maintaining a 1:1 molar ratio of the nitrogenous base and amino acids. The JLSV were assembled by mixing JBNT, laminin, VEGF, and SDF‐1α (2 × 10^3^:16:4:1 mass ratio) in deionized water, followed by sonication using a Sonicator (Q Sonica; Sonicators) at 100% amplitude for 2 min and 30 s. The prepared JLSV scaffold was mixed with BMSCs. The final concentration of JLSV was 20 µg mL^−1^, and that of BMSCs was 2 × 10^5^ cells mL^−1^. A sterile pipette was used to gently pipette up and down to ensure thorough mixing.

### UV–Vis Absorption Spectra Measurement

Groups of samples were prepared. For the JBNT group, 1 µL 1 mg mL^−1^ JBNTs was added to 49 µL deionized water to obtain a 20 µg mL^−1^ JBNT solution. For the laminin group, 40 µL 200 ng mL^−1^ laminin was added to 10 µL deionized water to obtain a 160 ng mL^−1^ laminin solution. For the VEGF group, 10 µL 200 ng mL^−1^ VEGF was added to 40 µL deionized water to obtain a 40 ng mL^−1^ VEGF solution. For the SDF‐1α group, 2.5 µL 200 ng mL^−1^ SDF‐1α was added to 47.5 µL deionized water to obtain a 10 ng mL^−1^ SDF‐1α solution. For the JBNT/VEGF group, 1 µL 2 µg mL^−1^ VEGF and 1 µL 1 mg mL^−1^ JBNTs were added to 48 µL deionized water. For the JBNT/laminin group, 4 µL 2 µg mL^−1^ laminin and 1 µL 1 mg mL^−1^ JBNTs were added to 45 µL deionized water. For the JBNT/SDF‐1α group, 0.25 µL 2 µg mL^−1^ SDF‐1α and 1 µL 1 mg mL^−1^ JBNTs were added to 48.75 µL deionized water. For the JLSV group, 4 µL 2 µg mL^−1^ laminin, 1 µL 2 µg mL^−1^ VEGF, 0.25 µL 2 µg mL^−1^ SDF‐1α, and 1 µL 1 mg mL^−1^ JBNTs were added to 43.75 µL deionized water and thoroughly mixed by pipetting several times. The final JBNT, laminin, SDF‐1α, and VEGF concentrations in the JLSV group were 20 µg mL^−1^, 160 ng mL^−1^, 10 ng mL^−1^, and 40 ng mL^−1^, respectively. The absorbance spectra of each sample group were measured using a NanoDrop spectrophotometer (Thermo Fisher Scientific, USA).

### TEM Characterization

First, 10 µL 1 mg mL^−1^ JBNTs were diluted with 40 µL distilled water to obtain a 200 µg mL^−1^ JBNT solution. Next, 4 µL 2 µg mL^−1^ laminin, 1 µL 2 µg mL^−1^ VEGF, 0.25 µL 2 µg mL^−1^ SDF‐1α, and 1 µL 1 mg mL^−1^ JBNTs were added to 43.75 µL deionized water and thoroughly mixed several times to prepare the JLSV sample. Then, 1 × 10^5^ BMSCs were added to 50 µL JLSV solution and thoroughly mixed several times to prepare the JLSV–BMSC microtissue sample. The three grids were cleaned using a Harrick Plasma PDC‐32G plasma cleaner before negative staining. The negative staining procedure was performed as follows: 3 µL JBNT solution (200 µg mL^−1^), 3 µL JLSV solution, and 3 µL JLSV–BMSC microtissue solution were each dropped onto separate grids and allowed to stand for 2 min. Then, 100 µL 0.5% uranyl acetate solution was pipetted onto each grid to rinse the solution. Excess liquid was removed from the grids using a filter paper, and the grids were allowed to dry. Finally, Lab6 20–120 kV TEM (Talos F200X, USA) was used to characterize the samples.

### Cryo‐SEM

The samples were rapidly frozen in liquid nitrogen, transferred to a cryo‐preparation chamber (Quorum PP3010T, UK), and sublimated at −90 °C for 10 min to remove surface frost. After sputter‐coating with platinum, the samples were imaged using a Zeiss Gemini SEM 500 (Germany) at 3 kV.

### Rheological Characterization

The rheological properties of hydrogel samples were assessed at 37 °C using a Thermo Scientific HAAKE RheoStress 1 rheometer equipped with an 8 mm diameter plate. To prepare JLSV samples for testing, 400 µL precursor solution was injected into a cylindrical mold with an 11 mm base diameter. The samples were then removed from the mold and placed between the upper and lower plates of the rheometer, with a 3 mm gap between the plates. Strain sweeps were performed from 0.1% to 100%, followed by frequency sweeps from 0.1 to 100 Hz at 1% constant strain.

### JLSV Storage

The two main components of JLSV were JBNTs and three active proteins (SDF1‐α, laminin, and VEGF). The JBNTs were stored at 4 °C, whereas the three proteins were stored at −80 and −20 °C for long‐ and short‐term storage, respectively. During preparation, the proteins were thawed at 4 °C and then assembled on an ice tray in the correct proportions. Once assembled, the JLSV scaffold can be stored at 4 °C for short‐term use, typically within 24 h.

### BMSC Isolation and Culture

BMSCs were isolated and cultured as previously described.^[^ ^48^ **^]^** Bone marrow was flushed from the femurs and tibias of 4‐week‐old C57BL/6 mice (Beijing Vital River Laboratory Animal Technology) with Minimum Essential Medium α (α‐MEM; Gibco, USA) without fetal bovine serum (FBS; Gibco, USA). The cell pellet was resuspended in α‐MEM supplemented with 15% FBS and cultured at 37 °C in a humidified incubator with 5% CO_2_ (Thermo Fisher, USA). BMSCs from passage five, which showed optimal growth, were used for subsequent experiments. The cells were treated with JLSV for 12 h under normoxic or OGD conditions.

### Cardiac Fibroblast Isolation and Culture and Cardiomyocyte Culture

Cardiac fibroblasts were isolated from neonatal mice as previously described ^[^ ^49^ ^]^ and cultured in DMEM (Dulbecco's Modified Eagle Medium) (Gibco, USA) with 10% FBS and 1% penicillin/streptomycin (Gibco, USA) at 37 °C under 5% CO_2_ in a humidified incubator. Cardiomyocyte AC16 cells were cultured in DMEM/F12 supplemented with 10% fetal bovine serum (FBS, Gibco, USA), 100 U mL^−1^ penicillin, and 100 µg mL^−1^ streptomycin.

### OGD Injury

The BMSCs were subjected to OGD injury as previously described.^[^ ^50^ ^]^ Briefly, the BMSCs were exposed to glucose‐free DMEM (Gibco, USA) and incubated in a hypoxic chamber (94% N_2_, 5% CO_2_, 1% O_2_; Thermo Fisher, USA) for 12 h at 37 °C. The control groups were cultured under normoxic conditions (95% air and 5% CO_2_; Thermo Fisher Scientific) in complete medium.

### Assay for Tube Formation

Matrigel matrix (BD Biosciences, USA) was thawed on ice and placed in 48‐well plates to form a gel layer. The cells were seeded onto Matrigel‐coated plates. After 12 h incubation under hypoxic conditions, the cells were incubated with a working solution containing 2 µm calcein‐AM (Thermo Fisher Scientific, USA) at 37 °C for 30 min, and tube formation was visualized using a confocal microscope (Zeiss LSM 880, Germany). The total area of branch structures per unit area was quantified using Image‐Pro Plus 6.0 (Media Cybernetics, Rockville, MD, USA).

### Live/Dead Cell Staining

Cell viability was determined using the Live/Dead Viability/Cytotoxicity Kit (Thermo Fisher Scientific, USA) according to the manufacturer's instructions. The cells were incubated with a working solution containing 2 µm calcein‐AM and 4 µM PI at 37 °C for 30 min. Calcein‐AM stains living cells green by intracellular esterase activity, whereas PI stains dead cells red by binding to DNA. Fluorescent images were captured using a confocal microscope (Zeiss LSM 880, Germany). The numbers of live (green) and dead (red) cells were quantified in five randomly selected fields per sample using Image‐Pro Plus 6.0. All experiments were performed in quintuplicate, and data were analyzed in a blinded manner.

### Annexin V–PI Staining

After treatment, the BMSCs were trypsinized the BMSCs were directly centrifuged before performing the Annexin V–PI staining protocol (BD, USA). The protocol briefly includes washing cells with cold 1× PBS once, suspending 1 × 10^6^ cells with 1× binding buffer (100 µL), adding Annexin V (5 µL) and PI (5 µL), and finally completing the volume to 500 µL with 1× binding buffer. The cells were kept in the dark until assessment and analyzed by flow cytometry within 1 h. The cells in forward scatter‐side scatter (FSC‐SSC) plots were gated according to untreated cells, and the percentages classified into the four regions were calculated.

### Immunocytochemistry (Confocal Microscopy)

The cells were fixed in 4% paraformaldehyde for 15 min, permeabilized with 0.1% Triton X‐100 in PBS for 10 min, and blocked in 5% goat serum for 30 min at room temperature. Next, the cells were incubated with primary antibodies or PBS (served as a negative control) overnight at 4 °C. Appropriate Alexa Fluor‐conjugated secondary antibodies were used for fluorescent staining. The nuclei were stained with DAPI (Abcam, ab104139). Confocal images were acquired using a Zeiss LSM 710 confocal laser scanning microscope (Carl Zeiss). The following primary antibodies were used: alpha‐smooth muscle actin (α‐SMA; 1:200, Abcam), vimentin (1:200, Affinity), cardiac troponin I (cTnI; 1:200, Proteintech), GATA4 (1:200, Proteintech), factor VIII (FVIII; 1:200, Affinity), and CD31 (1:200, Abcam), Ki67 (1:200, Abcam).

### Cell Co‐Culture

BMSCs or JLSV–BMSC microtissues were co‐cultured with cardiac fibroblasts, HUVECs, or cardiomyocytes in a Transwell system (Corning, USA). BMSCs or JLSV–BMSC microtissues (1×10^5^ cells per well) were seeded in the upper chamber (pore size 0.4 µm), while cardiac fibroblasts, HUVECs, or cardiomyocytes (2 × 10^5^ cells per well) were seeded in the lower chamber. The cells were cultured in 10% FBS‐supplemented DMEM under normoxic or OGD conditions for 12 h. The Transwell membrane allowed soluble factors to diffuse between the two chambers without direct cell contact. At the end of the co‐culture period, the cells were harvested for further analysis.

### Transwell Migration Assay

Cell migration was investigated using a Transwell system with inserts having 8 µm pore size (Corning, USA). In brief, 5 × 10^4^ cells were seeded in 200 µL serum‐free medium in the upper chamber, while 600 µL complete medium containing 10% FBS as a chemoattractant was added to the lower chamber. The cells were cultured under normoxic or OGD conditions for 12 h. After incubation, non‐migrating cells on the upper side of the membrane were removed using a cotton swab. The cells that migrated to the underside were fixed in 4% paraformaldehyde and stained with crystal violet. The migrated cells were counted in five randomly selected fields per membrane under a light microscope.

### VEGF, Angiopoietin‐2, IGF‐1, and SDF‐1α Concentration Measurement

VEGF, SDF‐1α, IGF‐1, and angiopoietin‐2 concentrations in BMSC culture supernatants were quantified using ELISA kits (Keyanyun, Shandong, China) according to the manufacturer's protocol. All measurements were performed in a blinded manner to avoid bias. Standard curves were generated for each factor, and the concentrations were calculated using linear regression.

### Animals

Adult male C57BL/6J mice (8–12 weeks old, 20–25 g; Beijing Vital River Laboratory Animal Technology) were housed in a temperature‐controlled facility with a 12‐h light‐dark cycle and unrestricted access to water and standard rodent chow, as previously described. All animal experiments were performed in accordance with the guidelines approved by the Animal Care and Use Committee of Qilu Hospital, Shandong University (Protocol ID: DWLL‐2022‐117), and conformed to the *Guide for the Care and Use of Laboratory Animals* (National Academies Press, USA).

### Animal Models with MI

MI was induced in mice by permanent ligation of the left anterior descending coronary artery (LAD). Successful occlusion was confirmed by pallor of the anterior wall of the left ventricle (LV). Male mice (8–12 weeks old, 20–25 g) were randomly divided into two cohorts. In the first cohort, the mice were assigned to the following groups: Sham, Sham+BMSCs, Sham+JLSV–BMSC microtissue, MI, MI+BMSCs, and MI+JLSV–BMSC microtissue. The second cohort was assigned to the following groups: Sham, Sham+JLSV, Sham+JLSV–BMSC microtissue, MI, MI+JLSV, and MI+JLSV–BMSC microtissue.

### RFP and PKH26 Labeling

The BMSCs were transfected with a lentiviral vector encoding RFP cDNA for stable expression. In addition, they were labeled with the red fluorescent dye PKH26 (Sigma) according to the manufacturer's protocol. The labeling efficiency was confirmed using fluorescence microscopy.

### BMSC Transplantation

BMSCs were transplanted immediately after MI induction. The volume of 2 × 10⁵ cells was controlled at 10 µL. The intramyocardial injection was strategically executed at three distinct sites: one injection directly targeted the ischemic area and the remaining two injections were administered to the surrounding border zones of the ischemia region using a Hamilton syringe with a 30‐gauge needle.

### In Vivo Metabolism and Degradation of JLSV

Cy7 fluorescent dye‐labeled JLSV was injected intramyocardially into the heart. The mice were divided into six groups: Sham, Sham+JLSV, Sham+JLSV–BMSCs, MI, MI+JLSV, and MI+JLSV–BMSCs. The fluorescence distribution was analyzed using an IVIS Spectrum in vivo imaging system (PerkinElmer, USA) on days 1, 3, 7, 14, and 28 post‐MI. Subsequently, the mice were euthanized, and their major organs were collected for *ex vivo* imaging. The DIR signal intensity at 780 nm was measured after excitation with a 750 nm laser.

### BMSC Engraftment and Differentiation Evaluation

In the first strategy, the transplanted BMSCs were labeled with the red fluorescent dye PKH26, and bioluminescence imaging (BLI) was performed using the IVIS Kinetic System (Caliper, Hopkinton, MA, USA) to monitor cell retention. The mice were imaged in vivo or *ex vivo* 3, 7, 14, and 28 days post‐transplantation. Peak signals (photons/s/cm^2^/sr) from predefined regions of interest (ROI) were quantified using Living Image 4.0 software (Caliper, MA, USA). In the second strategy, the transplanted BMSCs were transfected with a lentiviral vector encoding RFP mice were euthanized at each time point, and their hearts were fixed in 4% paraformaldehyde. Frozen heart sections (5 µm) were prepared for analysis. BMSCs expressing RFP were identified using intrinsic red fluorescence microscopy. The transplantation efficiency was quantified by calculating the percentage of RFP‐positive cells relative to DAPI‐positive nuclei in five sections per heart. To evaluate BMSC differentiation, cTnI (1:200, Proteintech), GATA4 (1:200, Proteintech), FVIII (1:200, Affinity), and CD31 (1:200, Abcam) were identified in the frozen heart sections using immunofluorescence staining.

### Blood Biochemistry, Blood Routine, and Inflammatory Cytokine Level Detection

Blood samples were collected on days 1, 3, 7, 14, and 28 post‐MI. Blood biochemistry analysis was conducted using an automatic biochemical analyzer. The parameters analyzed included alanine aminotransferase (ALT, U L^−1^), aspartate aminotransferase (AST, U L^−1^), AST/ALT ratio, albumin (ALB, g L^−1^), alkaline phosphatase (ALP, U L^−1^), total bilirubin (TBIL, µM), direct bilirubin (DBIL, µM), gamma‐glutamyl transferase (GGT, U L^−1^), blood urea (UREA, mg dL^−1^), and creatinine (CREA, mg dL^−1^). Routine blood tests were performed using an automatic blood cell analyzer. The parameters analyzed included white blood cell count (WBC, 10^9^ L^−1^), lymphocyte count (10^9^ L^−1^), monocyte count (10^9^ L^−1^), neutrophil count (10^9^ L^−1^), percentage of lymphocytes (%), percentage of monocytes (%), percentage of neutrophils (%), red blood cell count (RBC, 10^9^ L^−1^), hemoglobin (g L^−1^), hematocrit (HCT, %), mean corpuscular volume (MCV, fL), mean corpuscular hemoglobin (MCH, pg), mean corpuscular hemoglobin concentration (MCHC, g L^−1^), red blood cell distribution width (RDW, %), platelet count (PLT, 10^9^ L^−1^), mean platelet volume (MPV, fL), platelet distribution width (PDW, %) and platelet hematocrit (PCT, %). Serum CRP, TNF‐a, and IL‐6 levels were detected by ELISA kits.

### Echocardiographic Measurements

Transthoracic echocardiography using a Visual Sonics Vevo 2100 system with an MS‐400 linear transducer was used to measure ventricular remodeling and cardiac function. 2D parasternal images in M‐mode and grayscale images along the long axis were acquired for each mouse. The left ventricular end‐systolic volume (LVESV) and end‐diastolic volume (LVEDV) were calculated to determine the LVEF and FS.

### Histopathological Staining

Isolated heart tissues were immediately fixed in a 4% paraformaldehyde solution, followed by routine dehydration, paraffin embedding, and serial sectioning (5 µm).

Masson's trichrome staining was performed using a modified Masson's trichrome staining kit (Sigma–Aldrich).

Sirius red staining was performed using a Sirius red staining kit (Sigma, GER), according to established protocols.

### Immunofluorescence

Heart sections were deparaffinized in xylene and rehydrated in graded ethanol. Antigen retrieval was performed according to the methods recommended in the Antibody Manual. Endogenous peroxidase activity was quenched with 3% H_2_O_2_ for 15 min, and the sections were blocked in 5% goat serum for 30 min. Incubation with primary antibodies was performed overnight at 4 °C in a humidified chamber. The next day, the slides were incubated with Alexa Fluor‐conjugated secondary antibodies for 1 h at room temperature and mounted using a mounting medium containing DAPI (Abcam, ab104139).

### Western Blot Analysis

Cells or tissues were lysed in protein extraction buffer (Sigma) according to the manufacturer's instructions. Equal amounts of protein were separated on a 12% SDS–PAGE gel at 90 V for 1.5 h and transferred to PVDF (polyvinylidene fluoride) membranes (Millipore) at 150 mV for 1.5 h. The membranes were blocked with 5% nonfat milk (BD Biosciences) in 1× TBST for 1 h at room temperature and incubated overnight at 4 °C with the following primary antibodies: rabbit anti‐mouse α‐SMA (1:1000, Abcam), vimentin (1:1000, Affinity), collagen I (1:1000, Affinity), collagen III (1:1000, ABclonal), Bax (1:1000, Proteintech), Bcl2 (1:1000, Proteintech), cTnI (1:1000, Proteintech), GATA4 (1:1000, Proteintech), FVIII (1:1000, Affinity), and CD31 (1:1000, Abcam). After washing, the membranes were incubated with horseradish peroxidase‐conjugated secondary antibodies for 1 h at room temperature. The protein bands were visualized using an enhanced chemiluminescence system (Amersham Imager 800 RGB; GER, Tanon, China). All experiments were performed in quintuplicate, and data analysis was blinded.

### Statistical Analysis

All statistical analyses were performed using GraphPad Prism 5.0 (San Diego, CA, USA). Quantitative data were presented as mean ± standard error of the mean. The Shapiro–Wilk test was used to assess data distribution normality. For comparisons among three or more groups, one‐way analysis of variance (ANOVA) followed by the Bonferroni post‐hoc test was performed. Comparisons between two groups were performed using the Student's *t*‐test. Statistical significance was set at *p* < 0.05. ^*^ *p* < 0.05, ^**^ *p* < 0.01, and ^***^ *p* < 0.001 indicate statistical significance. All experiments were performed in quintuplicate, and data analysis was blinded.

## Conflict of Interest

The authors declare no conflict of interest.

## Author Contributions

P. H., L. Z., and L. Y. conceived the project and designed the experiments. L. Y. performed most of the cellular, biochemical, and animal experiments. H. A. and Q. L. partially contributed to the biochemical experiments. C. F. and H. Z. partially contributed to the animal experiments. Q. L. provided the technological support for biochemical and animal experiments. P. H. L. Z. and L. Y. edited the manuscript. P. Hao, L. Z., and Y. Z. provided financial support. All authors approved the final manuscript.

## Supporting information



Supporting Information

## Data Availability

The data that support the findings of this study are available from the corresponding author upon reasonable request.
