# Supplementary material for: Injectable BMSC‐Based Extracellular Matrix‐Mimicking Microtissue for Myocardial Infarction Repair
Source: Adv Sci (Weinh). 2025 Sep 15;13(3):e00299. doi: 10.1002/advs.202500299 (PMC12806379; doi:10.1002/advs.202500299)
Supplement: Supplementary file 1 — Supporting Information [file ADVS-13-e00299-s001.docx]

Supporting Information

Injectable BMSC-Based Extracellular Matrix-Mimicking Microtissue for Myocardial Infarction Repair

*Lina Yao, Huailong An, Cong Fan, Qingsu Lan, Hongyu Zhong, Yun Zhang, Libo Zhou*, Panpan Hao**


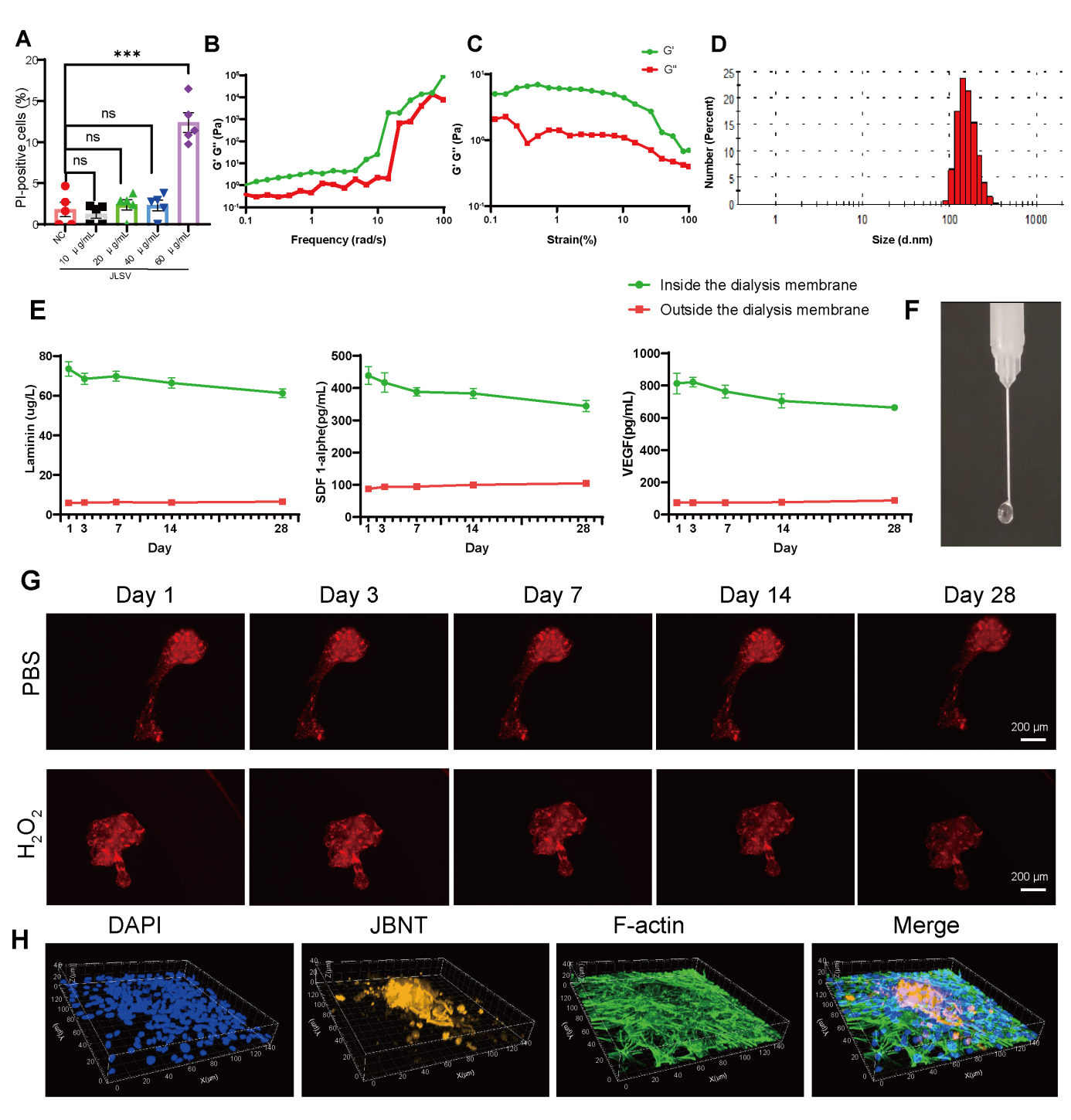


**Supplementary Figure 1.** Mechanical properties and material stability of JLSV. A) Quantitative analysis of PI-positive cells (%) presented in Figure 2J. B) Frequency-dependent variations of storage modulus (G') and loss modulus (G") for JLSV. C) Amplitude sweep measurements of JLSV. D) Particle size distribution of JBNTs in PBS solution. E) ELISA quantification of laminin, VEGF, and SDF-1α concentrations in the inner and outer dialysis bag compartments after incubating JLSV for 1, 3, 7, 14, and 28 days. F) Representative photograph demonstrating JLSV injectability. G) Fluorescence microscopy images of Alexa Fluor 555-labeled JLSV treated with hydrogen peroxide or PBS (control). H) 3D confocal microscopy images showing DAPI-stained nuclei (blue), Cy7-labeled JBNTs (yellow), and phalloidin-stained F-actin filaments (green).


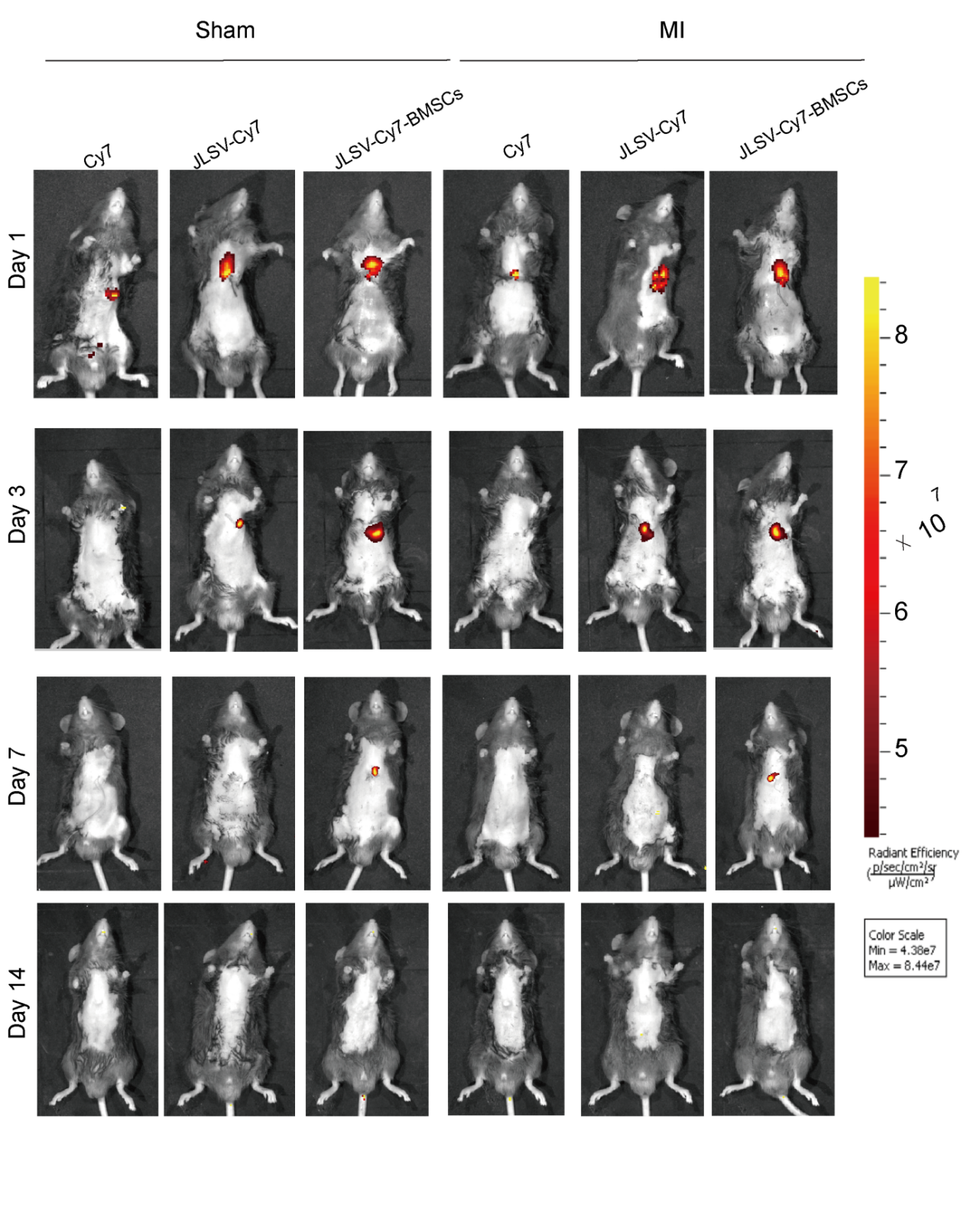


**Supplementary Figure 2.** *In vivo* degradation and metabolic imaging of JLSV. *In vivo* fluorescence imaging was performed at 1, 3, 7, and 14 days post-transplantation. The degradation status of both JLSV and JLSV-BMSC microtissues was quantified by measuring Cy7 fluorescence intensity.


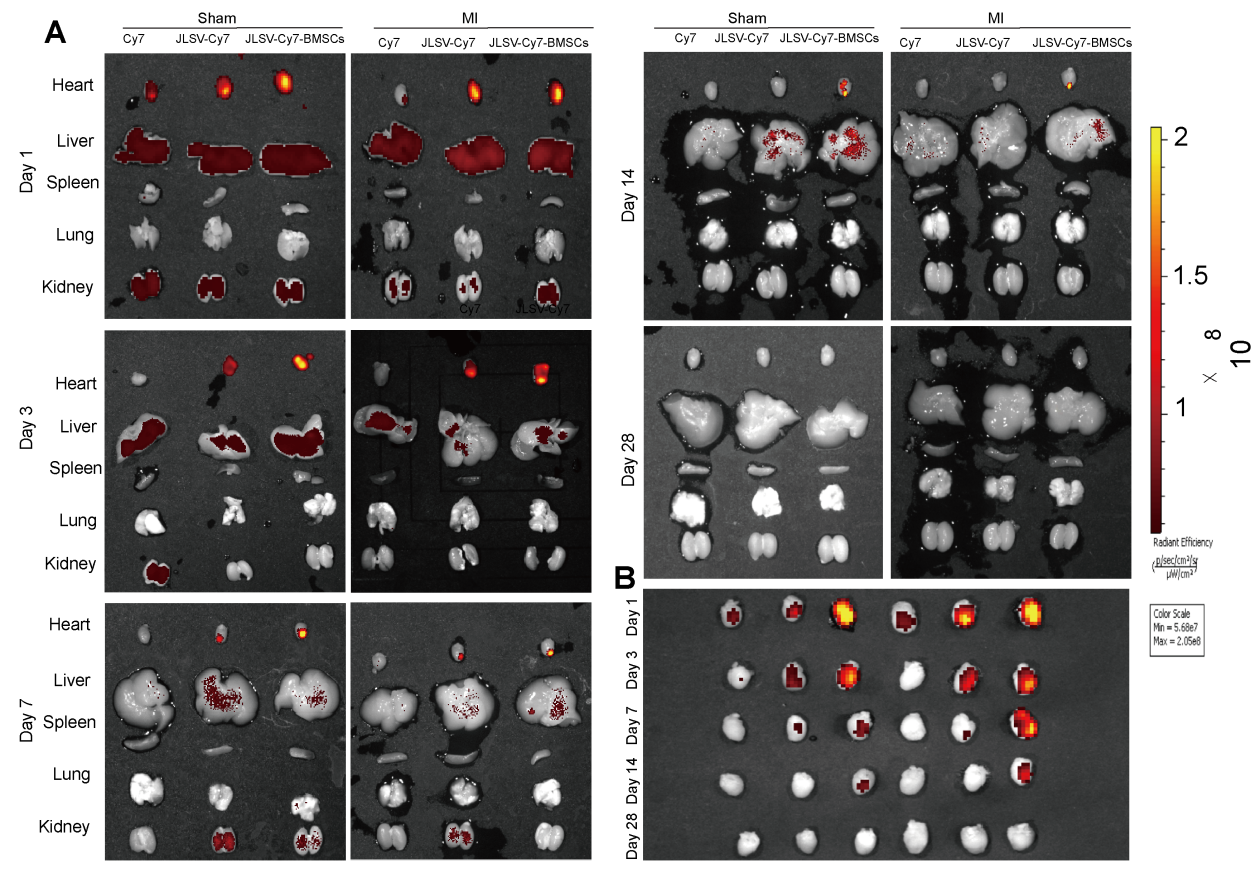

**Supplementary Figure 3.** *In Vivo* Degradation and Metabolic Imaging of JLSV. The retention and degradation kinetics of JLSV and JLSV-BMSC microtissues transplanted into the myocardium were quantified by measuring the radiance efficiency of Cy7 fluorescence signals. Representative *ex vivo* IVIS images are presented.


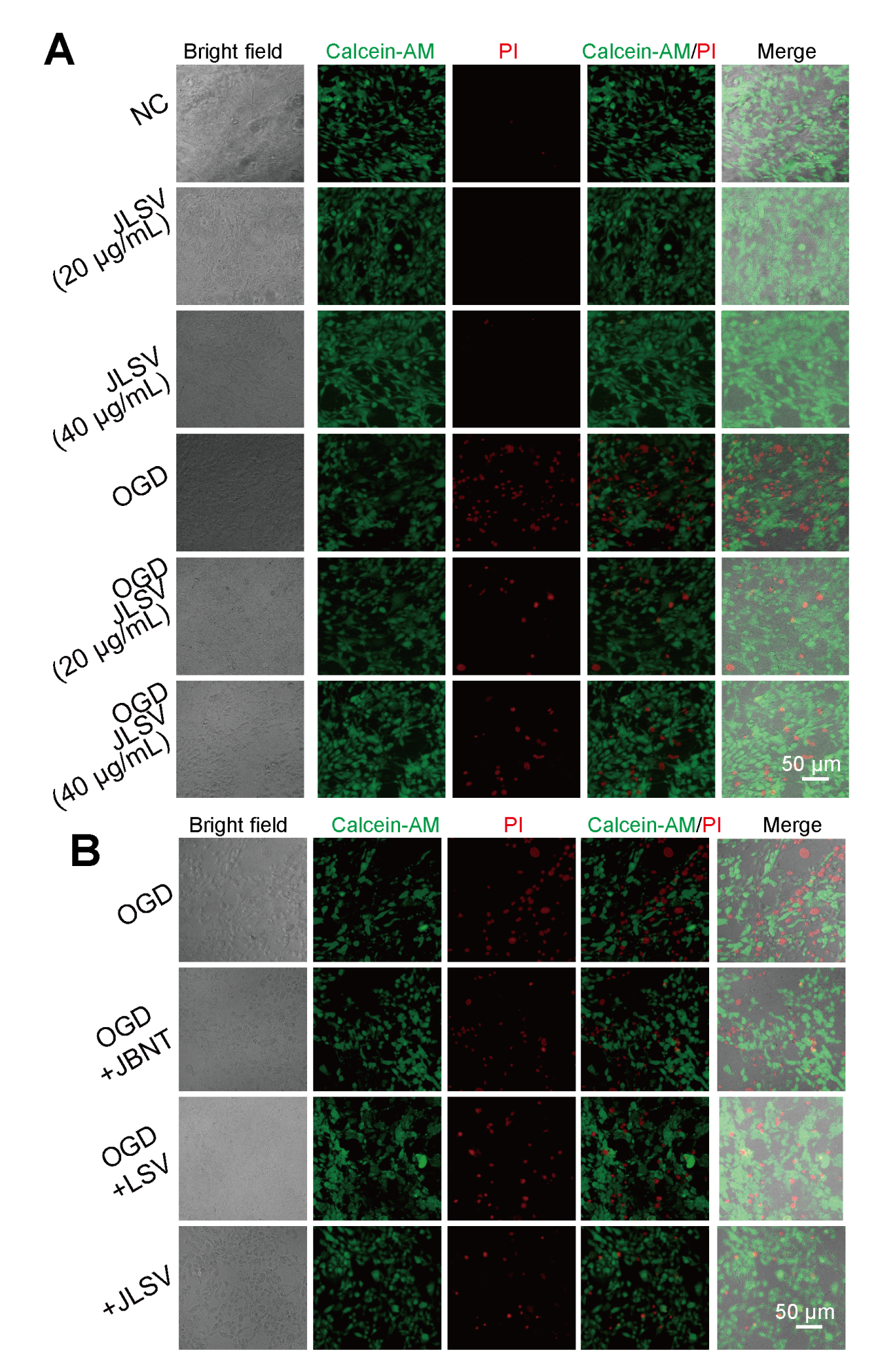


**Supplementary Figure 4** JLSV enhances BMSC survival. A) Calcein-AM (live)/propidium iodide (PI, dead) dual staining combined with bright-field microscopy was employed to evaluate BMSC viability with or without various concentrations of JLSV following OGD injury or under normoxic conditions. B) The same staining approach was used to assess BMSC viability when treated with individual JLSV components versus control media under OGD conditions.


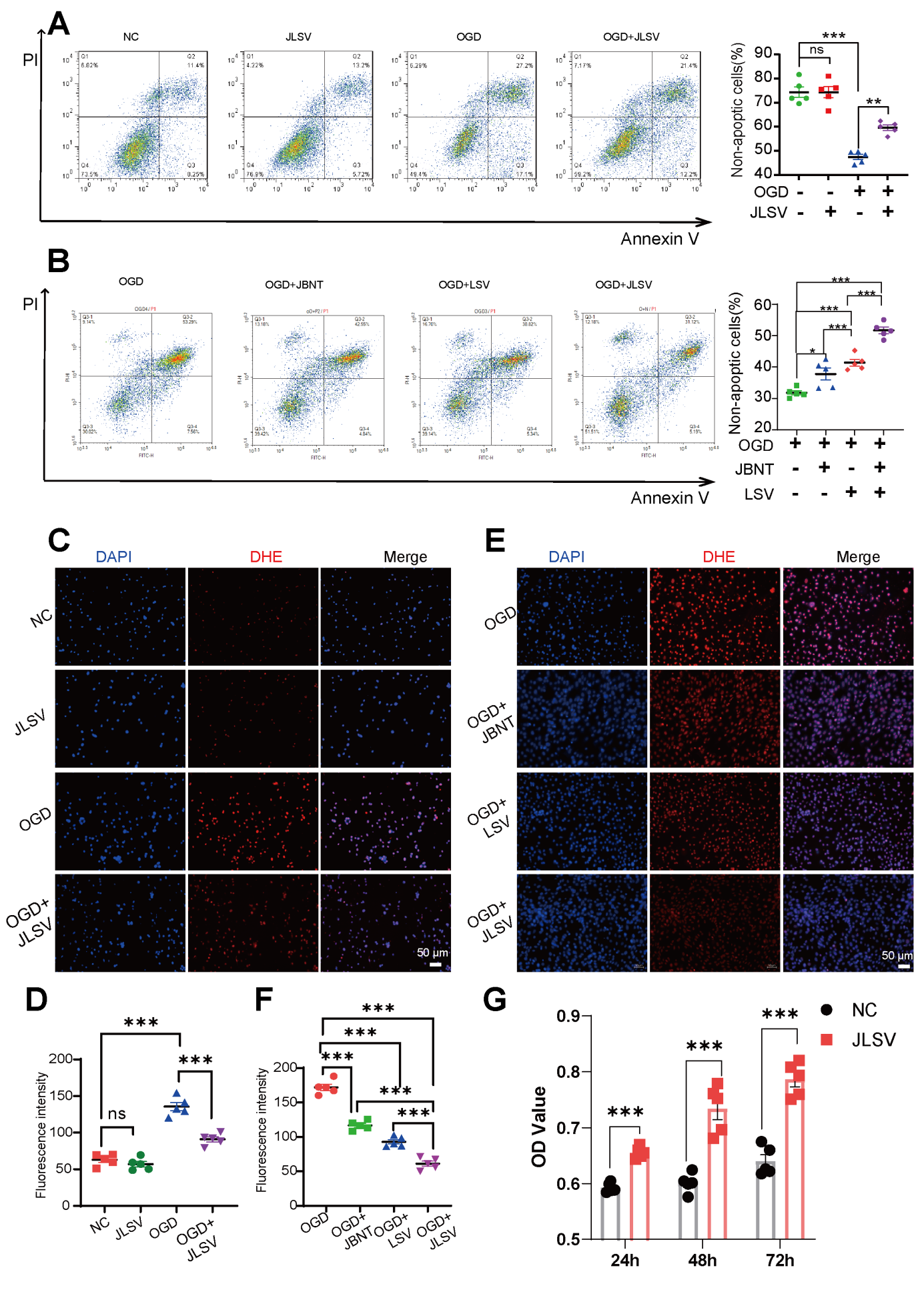


**Supplementary Figure 5.** JLSV Reduces Apoptosis and Oxidative Stress in BMSCs Under OGD Conditions A) Flow cytometric analysis of apoptosis in BMSCs cultured with or without JLSV under OGD or normoxic conditions. Apoptotic cells were classified into early apoptotic (Annexin V^+^/PI^-^) and late apoptotic (Annexin V^+^/PI^+^) populations. Data represent mean ± SEM (n=5). *p < 0.05, **p < 0.01, ***p < 0.001. B) Apoptosis assessment in BMSCs treated with JLSV components (JBNT, LSV), complete JLSV, or control media under OGD conditions. Data presentation as in A. C) Representative DHE staining images showing ROS levels in BMSCs cultured with or without JLSV under OGD or normoxic conditions. D) Quantification of DHE fluorescence intensity from 5 independent experiments. *p < 0.05, **p < 0.01, ***p < 0.001. E) Representative DHE staining of BMSCs treated with JLSV components or complete JLSV under OGD conditions. F) Quantification of DHE fluorescence intensity from 5 independent experiments. *p < 0.05, **p < 0.01, ***p < 0.001. G) CCK-8 assay results demonstrating the proliferative activity of BMSCs treated with JLSV or control media under normoxic conditions (24-72 h).

.
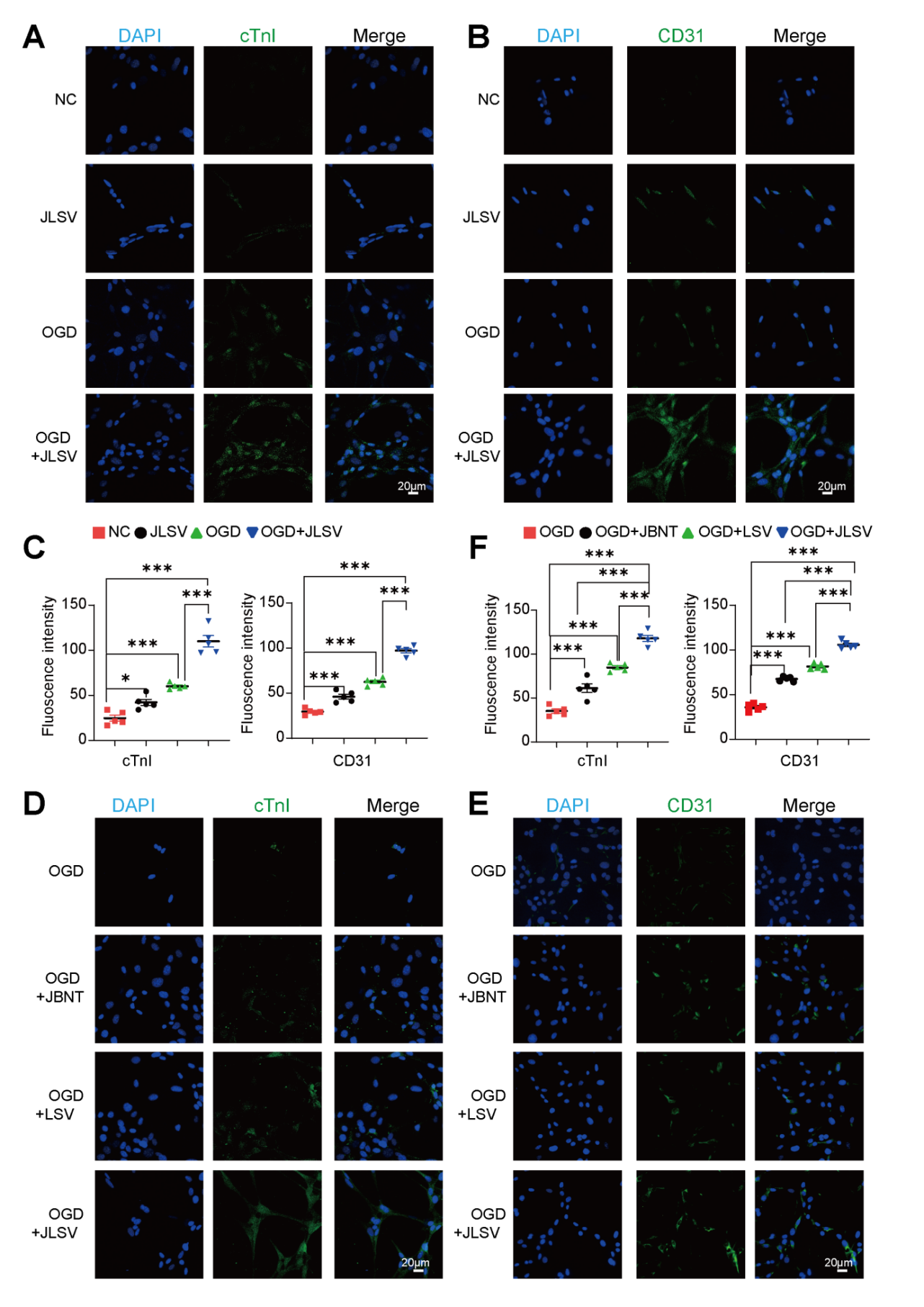


**Supplementary Figure 6.** JLSV Promotes BMSC Differentiation Under OGD Conditions

1. Immunofluorescence analysis of cardiomyogenic differentiation (cTnI^+,^ green) in BMSCs cultured ± JLSV under OGD or normoxic conditions. Nuclei were counterstained with DAPI (blue). B) Parallel immunofluorescence assessment of endothelial differentiation (CD31^+^, green) under identical culture conditions. C) Quantification of marker-positive cells from five independent fields per group (mean ± SEM, n=5). D) Cardiomyogenic differentiation (cTnI^+^,) in BMSCs treated with JLSV components (JBNT, LSV), complete JLSV, or control media under OGD. E) Endothelial differentiation (CD31^+^) in BMSCs treated with the same conditions as in D. F) Quantitative analysis as described in C.


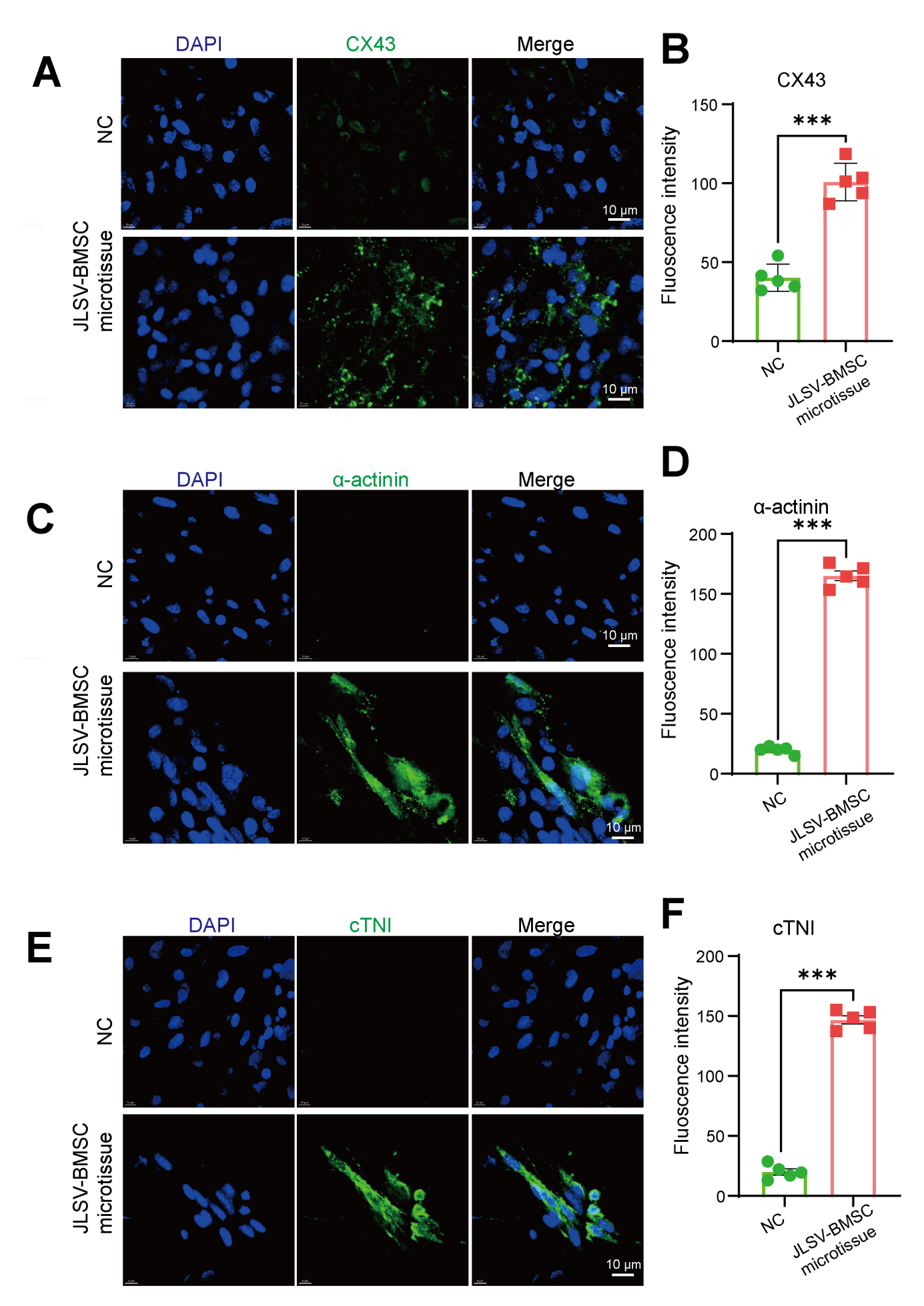


**Supplementary Figure 7.** After 21 days of *in vitro* culture, BMSCs begin differentiating towards a cardiomyocyte lineage. A) BMSCs were cultured for 21 days, and immunocytochemical staining was used to detect CX43 (green), with cell nuclei stained using DAPI (blue). B) Quantitative data were obtained from five independent images per group and expressed as mean ± standard error of the mean, n=5. C) BMSCs were cultured for 21 days, and immunocytochemical staining was used to detect α-actinin (green), with cell nuclei stained using DAPI (blue). D) Quantitative data were obtained from five independent images per group and expressed as mean ± standard error of the mean, n=5. E) BMSCs were cultured for 21 days, and immunocytochemical staining was used to detect cTnI (green), with cell nuclei stained using DAPI (blue). F) Quantitative data were obtained from five independent images per group and expressed as mean ± standard error of the mean, n=5.


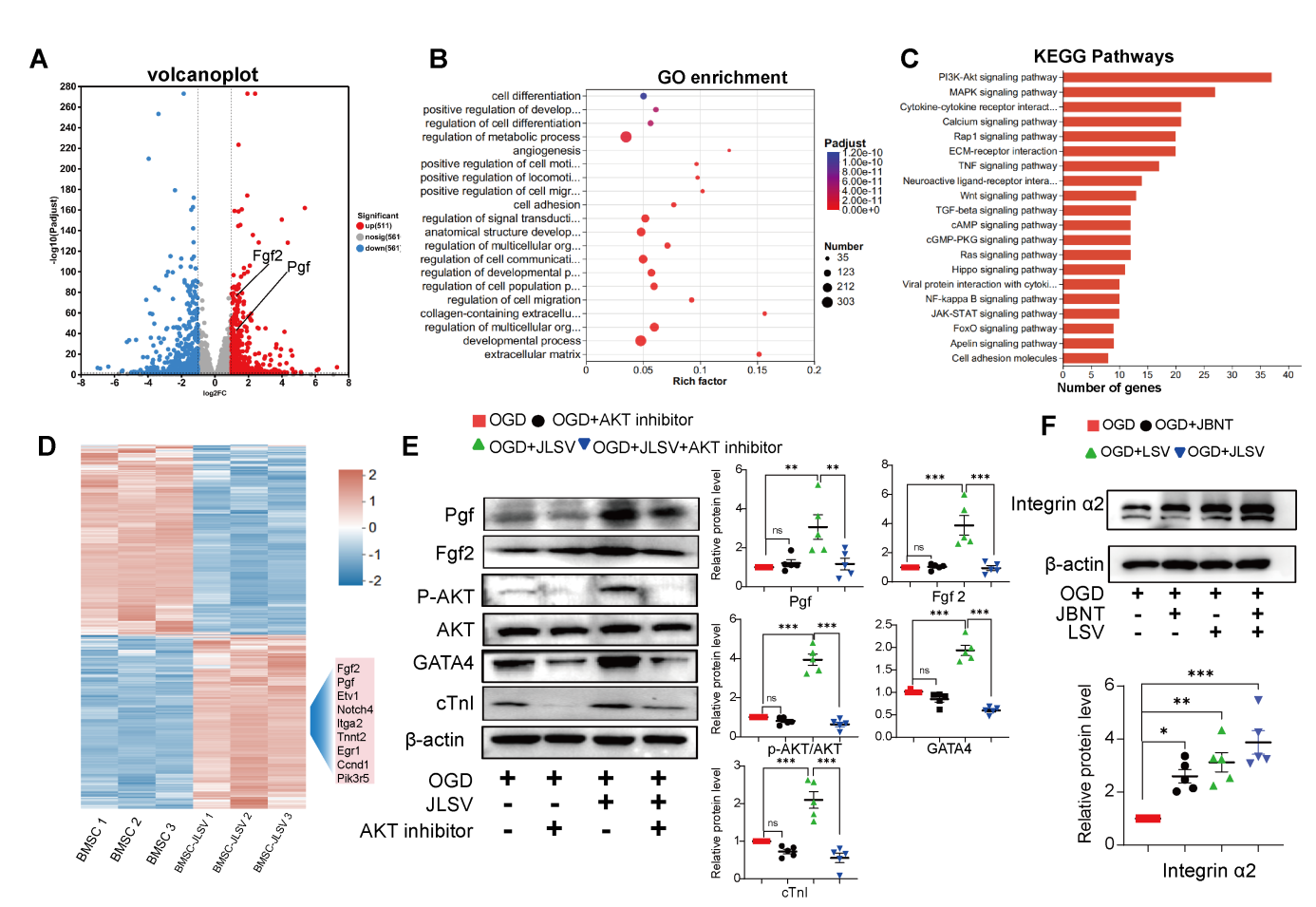

**Supplementary Figure 8.** RNA sequencing revealed the potential mechanisms by which JLSV facilitates the differentiation of BMSCs into cardiomyocytes. A) Volcano plot showing all differentially expressed genes (DEGs). B) Top 20 enriched Gene Ontology terms (FDR < 0.1, p < 0.05). C) Top 20 enriched Kyoto Encyclopedia of Genes and Genomes (KEGG) pathways (FDR < 0.1, p < 0.05). D) Hierarchical clustering heatmap and dendrogram of samples based on the all DEGs, with genes clustered by row and samples by column. E) BMSCs were incubated with JLSV, AKT inhibitor or control media under OGD conditions, and protein levels of p-AKT, AKT, GATA4, cTnI, Fgf2 and Pgf were measured. F) BMSCs were incubated with JBNT, LSV, complete JLSV or control media under OGD conditions. Itga2 protein level was determined by Western blotting. Data are expressed as the mean ± SEM, n = 5. Statistical significance is indicated as *p < 0.05, **p < 0.01, ***p < 0.001.


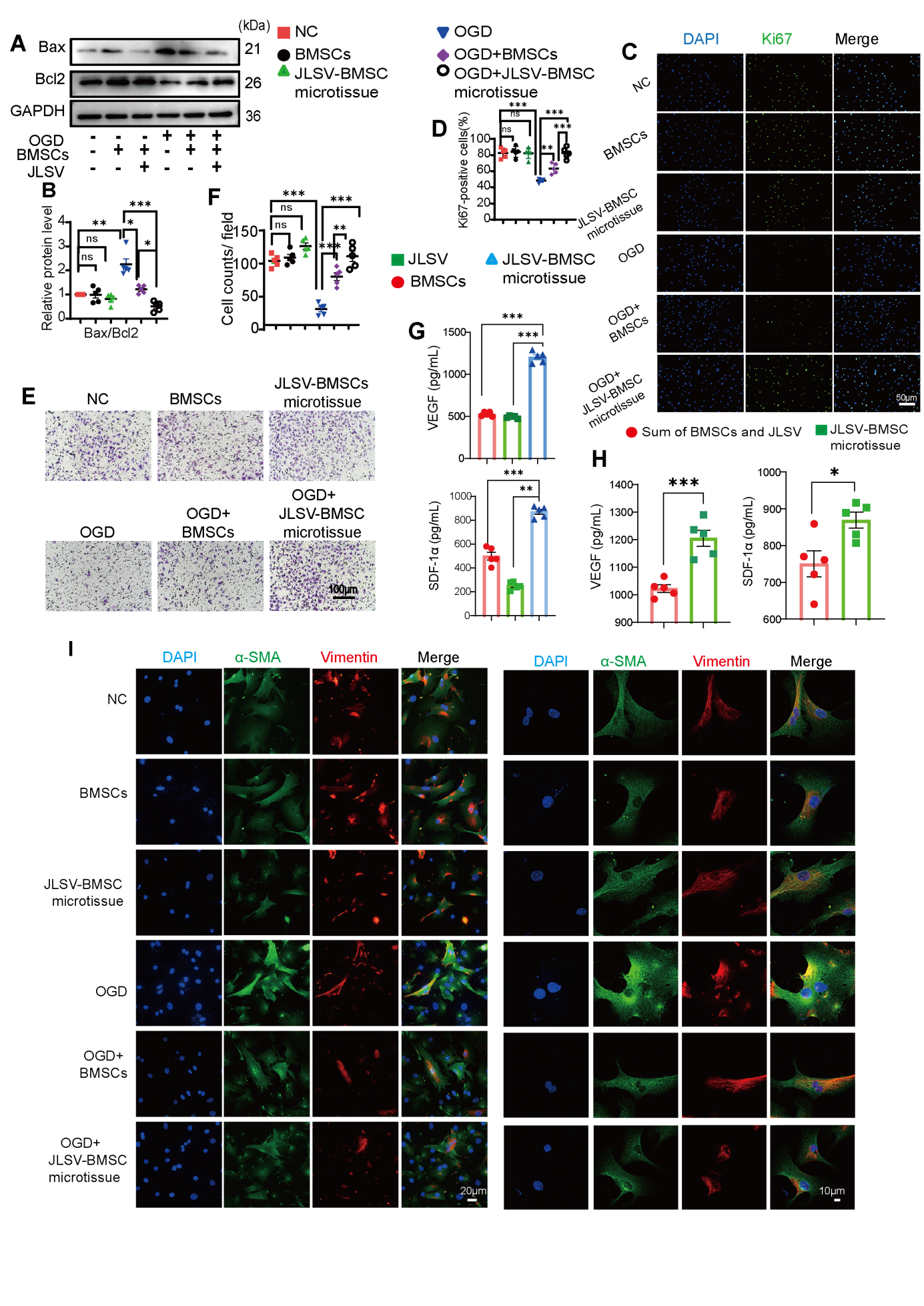


**Supplementary Figure 9.** JLSV-BMSC Microtissue Enhances Paracrine-Mediated Cellular Crosstalk A) Apoptosis regulation in HUVECs co-cultured with BMSCs or JLSV-BMSC microtissues under OGD/normoxia, assessed by Bcl-2/Bax ratio via Western blot. B) Quantification of apoptotic markers from five biological replicates (mean ± SEM). *p < 0.05, **p < 0.01, ***p < 0.001. C) Ki67 immunofluorescence (green) demonstrating HUVEC proliferation under different co-culture conditions. Nuclei were counterstained with DAPI (blue). D) Proliferation quantification from five independent experiments (mean ± SEM). *p < 0.05, **p < 0.01, ***p < 0.001. E) Transwell migration assay showing crystal violet-stained HUVECs (8 μm pore membrane). F) Migrated cell counts from five replicates (mean ± SEM). *p < 0.05, **p < 0.01, ***p < 0.001. G) Quantification of VEGF and SDF-1α in different culture groups (mean ± SEM, n=5). H) Comparative analysis of VEGF and SDF-1α secretion between the JLSV-BMSC microtissue group and the sum of the BMSC group and the JLSV group (mean ± SEM, n=5). I) Cardiac fibroblast activation assessed by α-SMA (green) and vimentin (red) immunofluorescence under different co-culture conditions. Nuclei were counterstained with DAPI (blue).


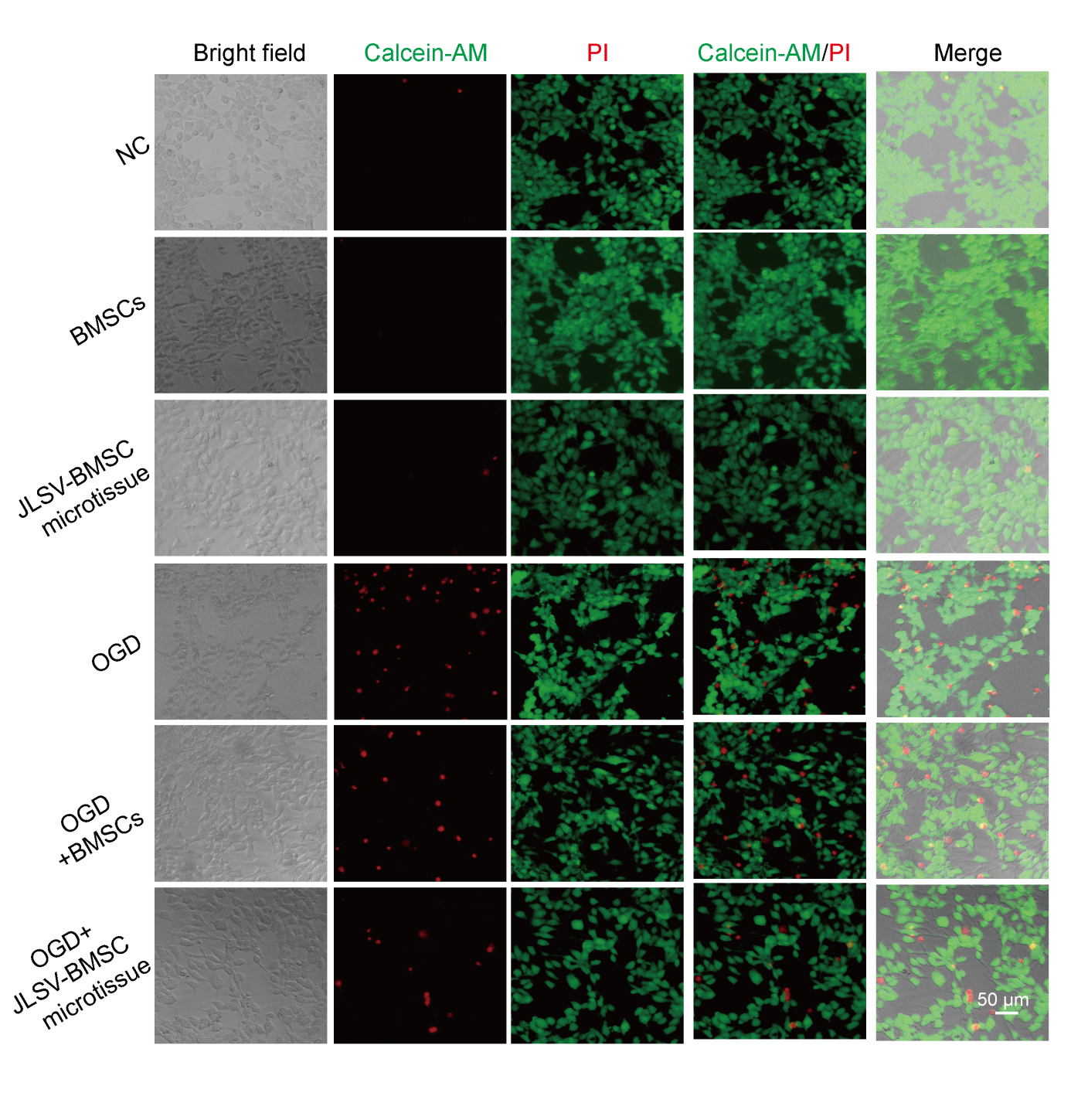


**Supplementary Figure 10** Cell viability was assessed using calcein-AM/PI dual staining (green: live cells; red: dead cells) and bright-field imaging in cardiomyocytes co-cultured with BMSCs or JLSV-BMSC microtissues under OGD or normoxic conditions.


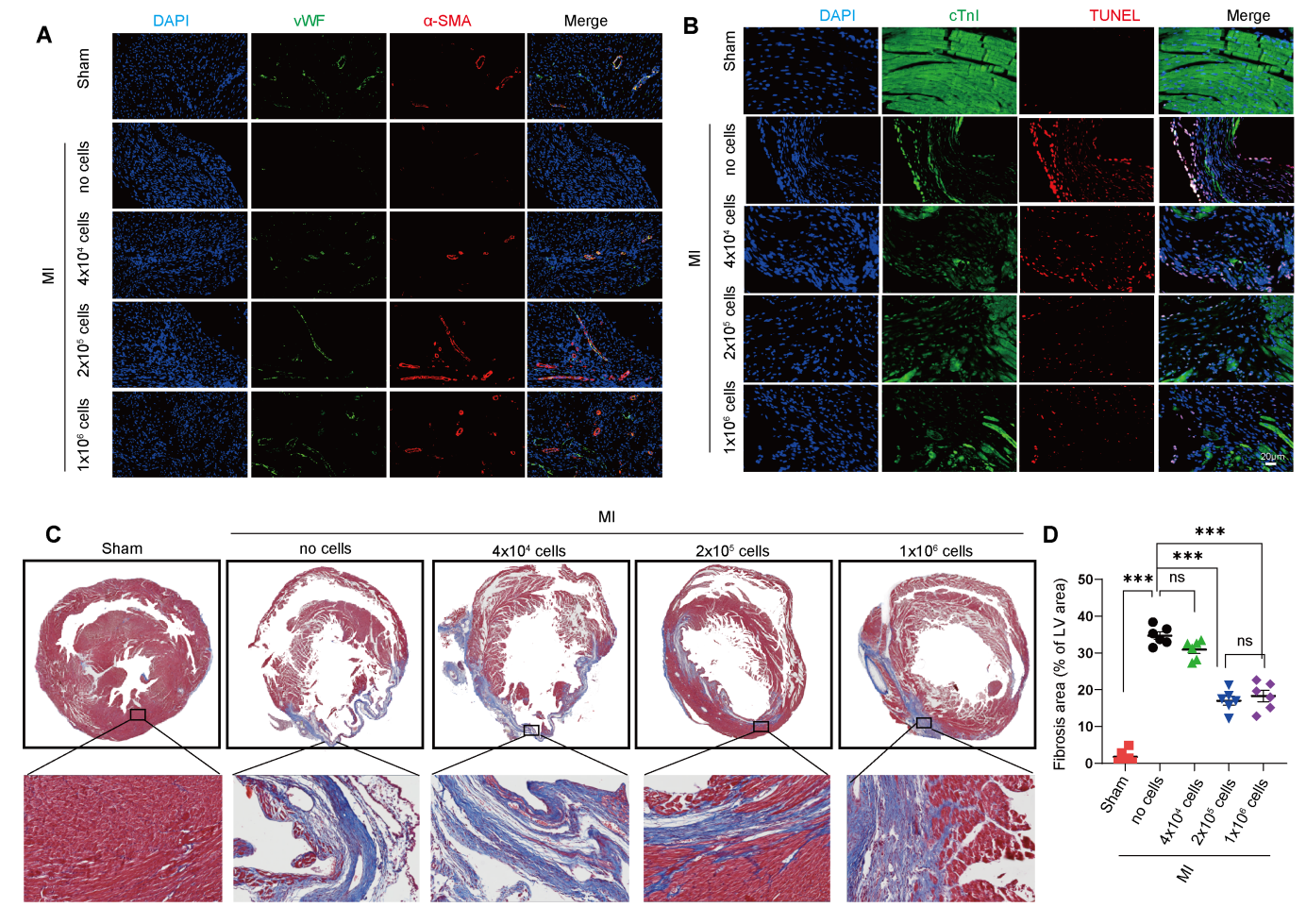

**Supplementary Figure 11.** Selection optimal dose of JLSV-BMSCs in mice. A) Representative immunofluorescence images of α-SMA-positive and vWF-positive blood vessels in the infarct region after 28 days post-implantation. Images are shown in the following groups: Sham, myocardial infarction (MI), MI + microtissue with 4×10^4^ cells, MI + microtissue with 2×10^5^ cells, and MI + microtissue with 1×10^6^ cells. B) Representative TUNEL staining images in the following groups: Sham, MI, MI + microtissue with 4×10^4^ cells, MI + microtissue with 2×10^5^ cells, MI + microtissue with 1×10^6^ cells. C) Masson’s trichrome staining of the whole heart in the following groups: Sham, MI, MI + microtissue with 4×104 cells, MI + microtissue with 2×10^5^ cells, MI + microtissue with 1×10^6^ cells. D) Quantitative analysis of the fibrosis area using Masson’s trichrome staining. Data are expressed as mean ± SEM from 5 independent mice per group. *p < 0.05, **p < 0.01, ***p < 0.001.


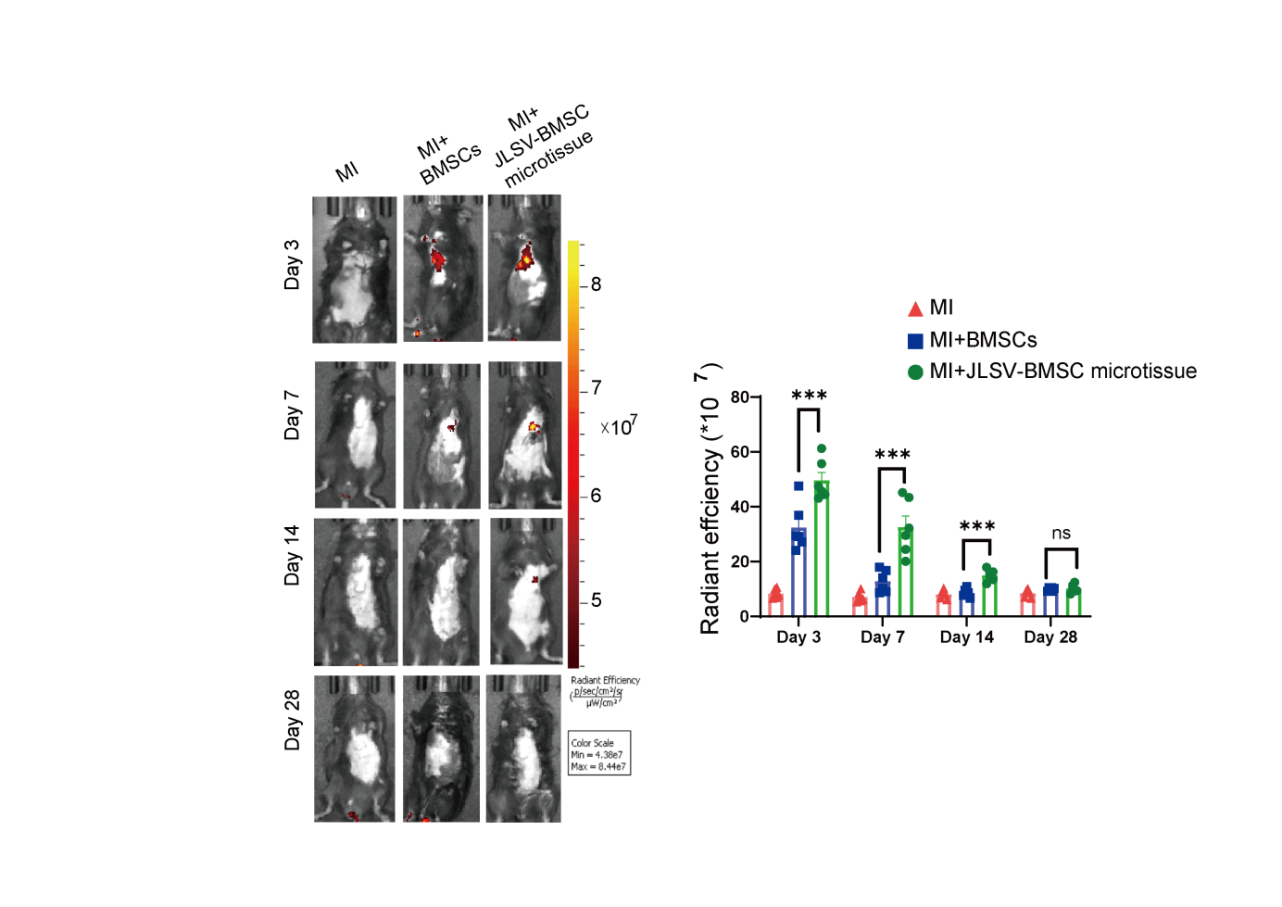


**Supplementary Figure 12.** *In Vivo* Fluorescence Imaging and Quantitative Analysis of BMSC Retention A) In vivo fluorescence imaging was performed at 3, 7, 14, and 28 days post-transplantation to track BMSC localization. B) Quantitative analysis of fluorescence intensity (expressed as radiant efficiency) was conducted to evaluate BMSC retention at the infarct site over time. Data represent mean ± SEM (n=6 mice per group). Statistical significance: *p<0.05, **p<0.01, ***p<0.001.


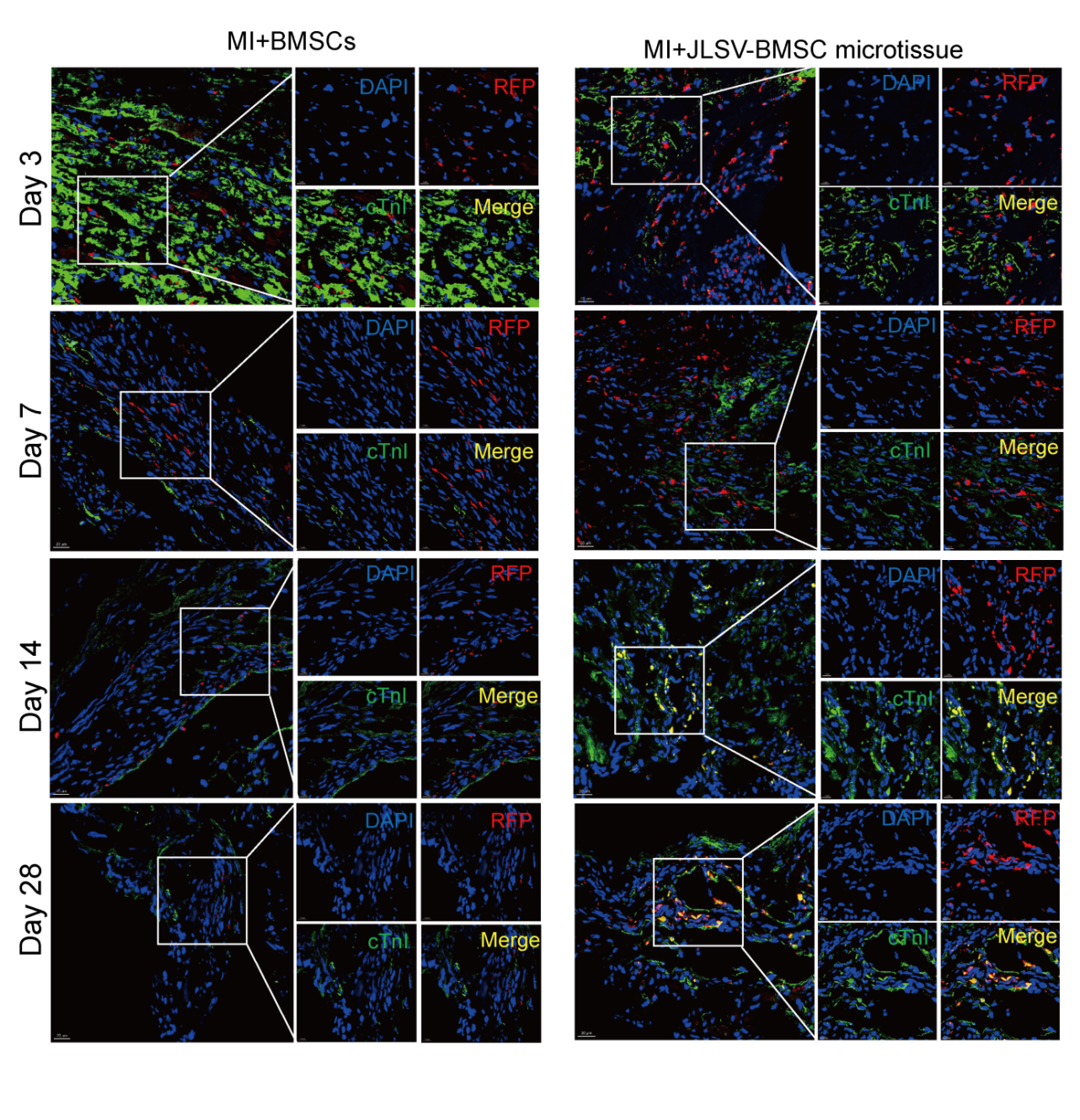


**Supplementary Figure 13.** Confocal immunofluorescence images of frozen sections demonstrating RFP-expressing BMSCs (red) colocalized with the cardiomyocyte-specific marker cTNI (green) at 3, 7, 14, and 28 days post-transplantation. Nuclei were counterstained with DAPI (blue).


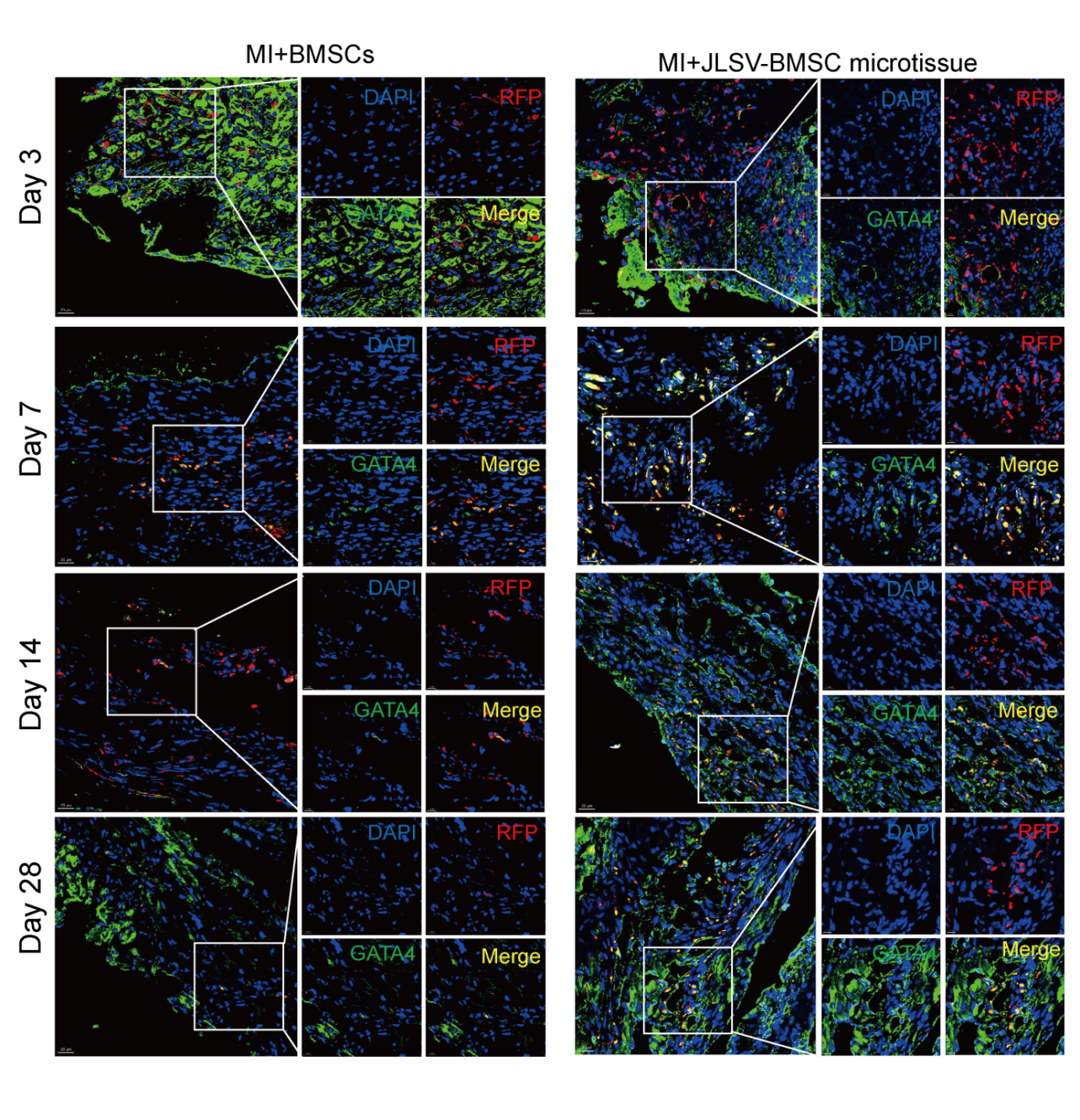


**Supplementary Figure 14.** Confocal immunofluorescence images of frozen sections demonstrating RFP-expressing BMSCs (red) colocalized with the cardiomyocyte-specific marker GATA4 (green) at 3, 7, 14, and 28 days post-transplantation. Nuclei were counterstained with DAPI (blue).


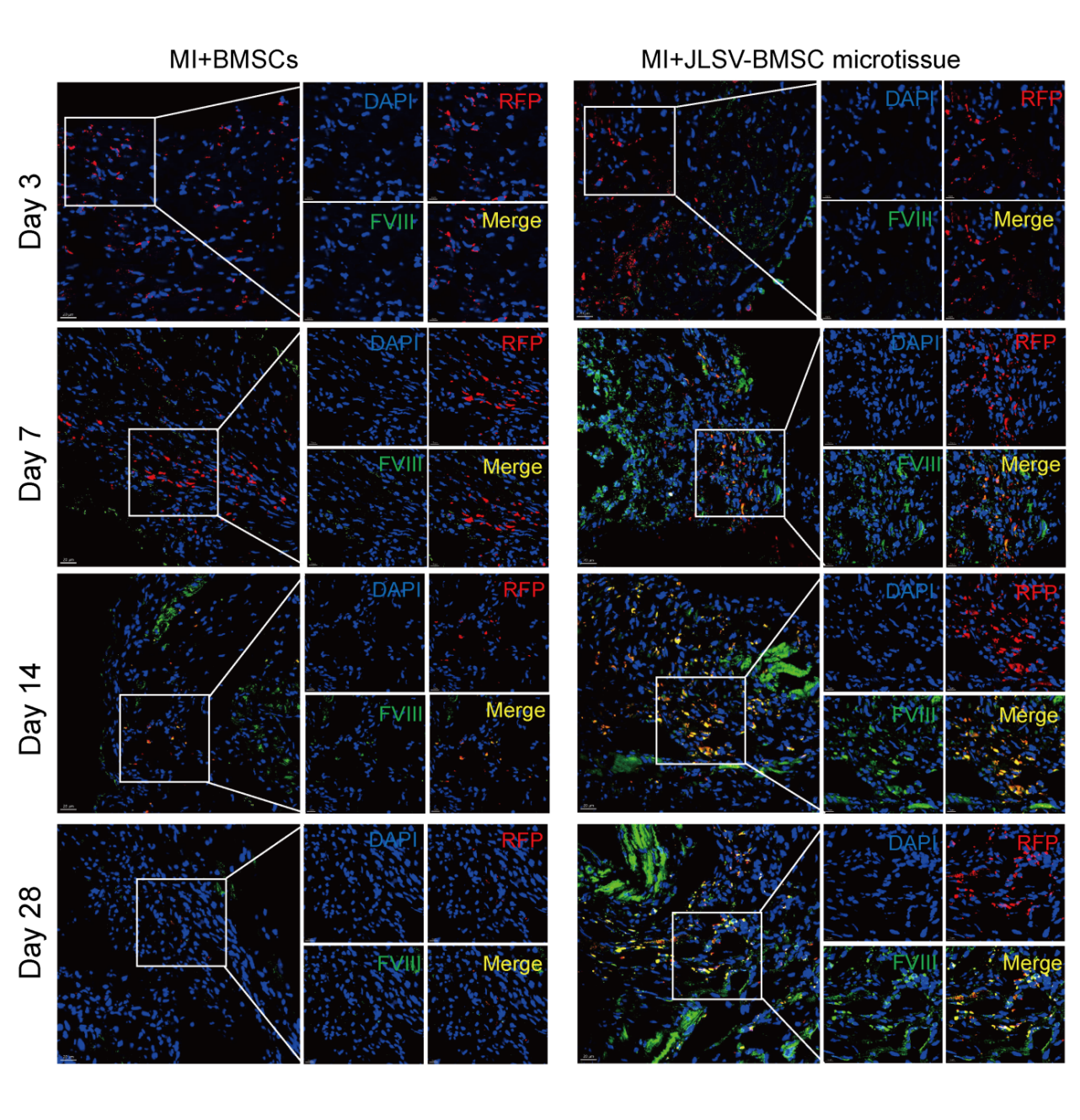


**Supplementary Figure 15.** Confocal immunofluorescence images of frozen sections demonstrating RFP-expressing BMSCs (red) colocalized with the endothelial-specific marker factor VIII (FVIII, green) at 3, 7, 14, and 28 days post-transplantation. Nuclei were counterstained with DAPI (blue).


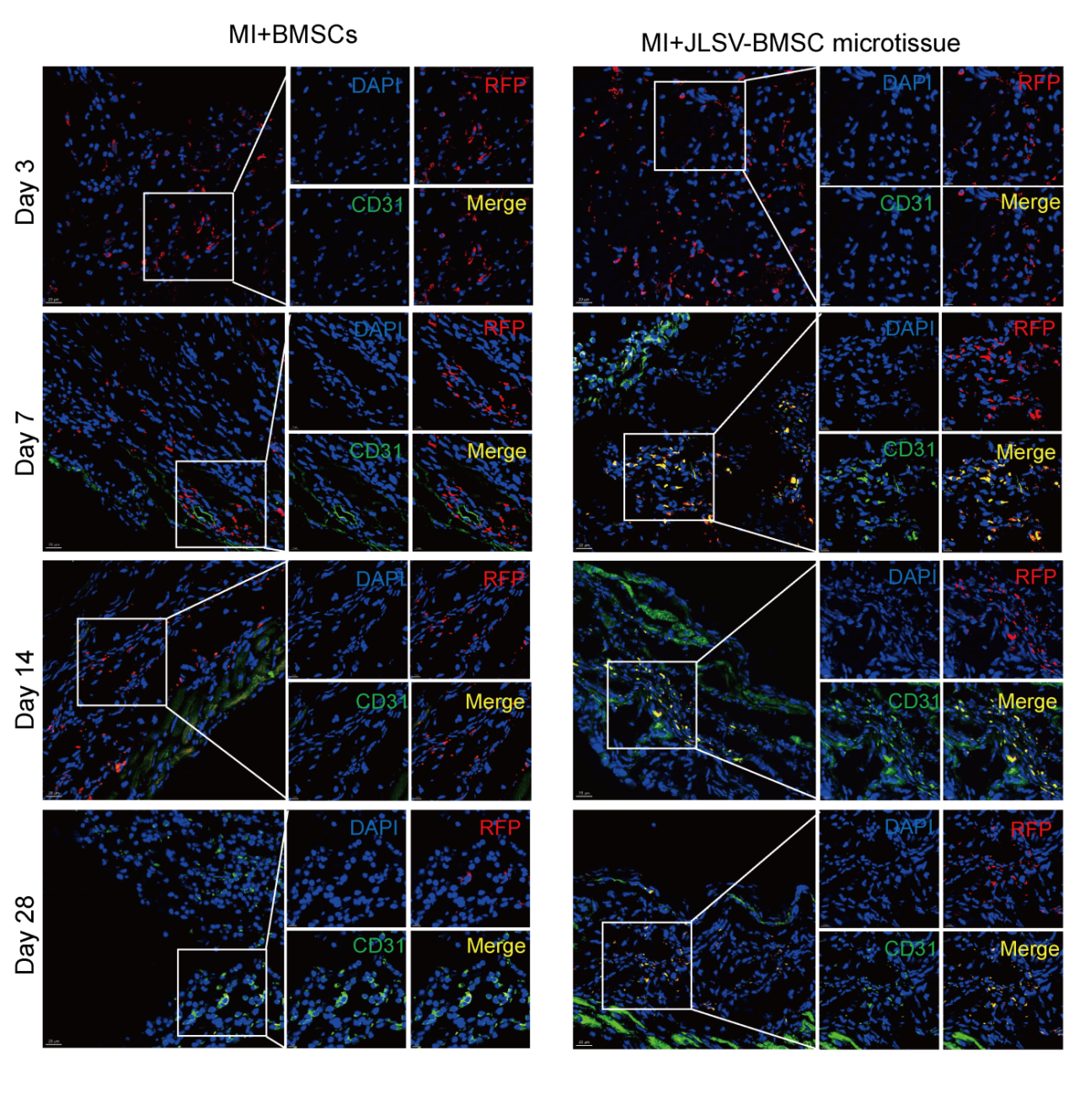


**Supplementary Figure 16.** Confocal immunofluorescence images of frozen sections demonstrating RFP-positive BMSCs (red) colocalized with the endothelial-specific marker CD31 (green) at 3, 7, 14, and 28 days post-transplantation. Nuclei were counterstained with DAPI (blue).


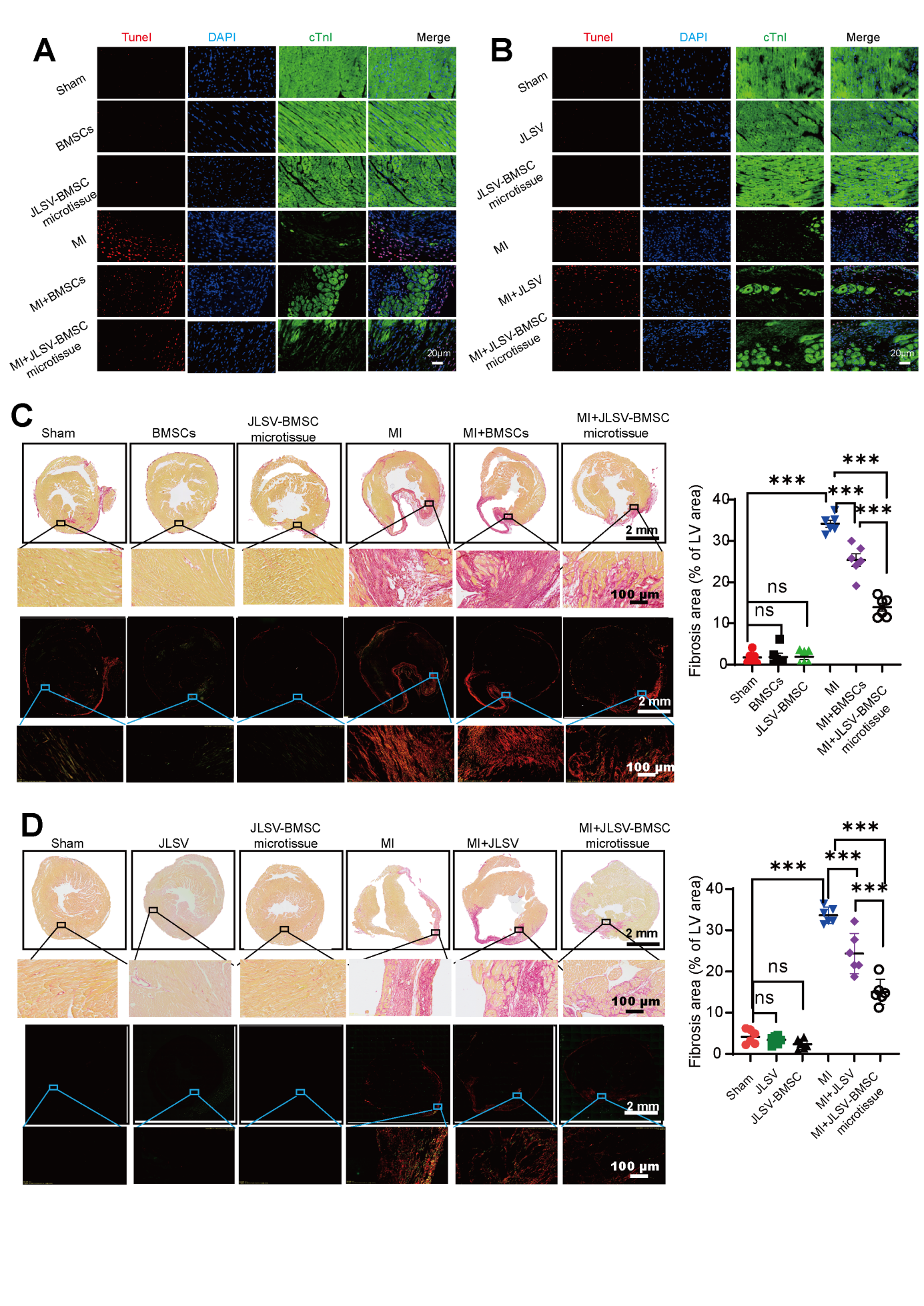


**Supplementary Figure 17.** JLSV-BMSC microtissue protects the myocardium from apoptosis and alleviates cardiac fibrosis post-myocardial infarction. A) Representative TUNEL staining images from the following groups: Sham, Sham + BMSCs, Sham + JLSV-BMSC microtissue, MI + saline, MI + BMSCs, and MI + JLSV-BMSC microtissue. B) Representative TUNEL staining images from the following groups: Sham, Sham + JLSV, Sham + JLSV-BMSC microtissue, MI + saline, MI + JLSV, and MI + JLSV-BMSC microtissue. C) Sirius red staining of the whole heart in the following groups: Sham, Sham + BMSCs, Sham + JLSV-BMSC microtissue, MI + saline, MI + BMSCs, and MI + JLSV-BMSC microtissue. Representative images of the infarct zone on day 28 after myocardial infarction are shown. Quantitative analysis is expressed as mean ± SEM of 6 independent mice per group. *p < 0.05, **p < 0.01, ***p < 0.001. D) Sirius red staining of the entire heart in the following groups: Sham, Sham + JLSV, Sham + JLSV-BMSC microtissue, MI + saline, MI + JLSV, and MI + JLSV-BMSC microtissue. Representative images of the infarct zone on day 28 after myocardial infarction are shown. Quantitative analysis is expressed as mean ± SEM of 6 independent mice per group. *p < 0.05, **p < 0.01, ***p < 0.001.

**Supplementary Table 1**  Blood Routine Examination on Day 1

| Parameter | Sham | Sham+JLSV | Sham+JLSV-BMSC | MI | MI+JLSV | MI+JLSV-BMSC |
| --- | --- | --- | --- | --- | --- | --- |
| WBC (10^9^ L^−1^) | 3.99±0.82 | 4.32±0.82 | 3.52±0.48 | 4.03±0.50 | 3.33±0.49 | 5.68±0.74 |
| Lymphocyte (10^9^ L^−1^) | 2.35±0.44 | 2.35±0.44 | 2.08±0.34 | 2.56±0.24 | 1.98±0.21 | 3.24±0.61 |
| Monocyte (10^9^ L^−1^) | 0.31±0.08 | 0.44±0.23 | 0.20±0.09 | 0.23±0.09 | 0.20±0.12 | 0.54±0.13 |
| Neutrophil (10^9^ L^−1^) | 1.08±0.27 | 1.25±0.12 | 1.03±0.19 | 0.81±0.21 | 0.99±0.13 | 1.35±0.12 |
| Lymphocyte (%) | 59.850±2.978 | 53.60±1.105 | 58.58±6.36 | 64.70±4.92 | 60.33±2.15 | 47.43±10.25 |
| Monocyte (%) | 8.20±1.72 | 8.23±3.65 | 5.23±1.83 | 5.50±1.62 | 4.88±2.28 | 7.50±1.95 |
| Neutrophil (%) | 25.85±4.57 | 31.35±4.52 | 30.18±6.40 | 23.53±2.99 | 29.98±1.21 | 34.33±7.73 |
| RBC (10^12^ L^−1^) | 5.21±0.60 | 6.60±0.98 | 5.85±0.63 | 6.61±0.21 | 5.77±0.43 | 5.56±0.63 |
| HGB (g L^−1^) | 127.30±12.71 | 134.00±16.01 | 147.50±14.71 | 166.70±2.15 | 143.60±9.93 | 141.00±14.26 |
| HCT (%) | 22.93±2.58 | 28.18±4.43 | 25.63±2.49 | 29.08±0.48 | 25.38±1.26 | 24.28±1.83 |
| MCV (fL) | 44.03±0.43 | 42.60±0.74 | 44.00±1.51 | 44.10±1.23 | 44.09±1.08 | 44.24±1.61 |
| MCH (pg) | 24.60±0.8396 | 21.75±3.60 | 25.25±0.41 | 25.25±0.47 | 24.88±0.16 | 25.59±0.37 |
| MCHC (g L^−1^) | 558.30±15.80 | 513.00±87.67 | 572.80±8.56 | 576.30±11.59 | 564.80±10.90 | 575.50±14.41 |
| PLT (10^9^ L^−1^) | 498.30±106.10 | 625.30±111.40 | 516.80±106.80 | 556.80±43.82 | 613.80±11.24 | 519.50±70.38 |
| MPV (fL) | 5.33±0.13 | 5.23±0.06 | 5.50±0.24 | 5.20±0.04 | 5.39±0.11 | 5.73±0.34 |
| PDW (%) | 14.70±0.11 | 14.65±0.10 | 14.55±0.07 | 14.58±0.05 | 14.55±0.07 | 15.15±0.39 |

**Supplementary Table 2** Blood Routine Examination on Days 3

| Parameter | Sham | Sham+JLSV | Sham+JLSV-BMSC | MI | MI+JLSV | MI+JLSV-BMSC |
| --- | --- | --- | --- | --- | --- | --- |
| WBC (10^9^ L^−1^) | 3.68±0.63 | 2.44±0.69 | 3.97±0.39 | 3.62±0.43 | 3.64±0.54 | 5.37±0.61 |
| Lymphocyte (10^9^ L^−1^) | 2.15±0.33 | 1.41±0.46 | 1.67±0.36 | 2.01±0.21 | 1.70±0.33 | 2.25±0.55 |
| Monocyte (10^9^ L^−1^) | 0.24±0.11 | 0.22±0.09 | 0.49±0.10 | 0.31±0.12 | 0.51±0.08 | 0.47±0.07 |
| Neutrophil (10^9^ L^−1^) | 1.05±0.25 | 0.65±0.18 | 1.65±0.11 | 1.07±0.20 | 1.21±0.36 | 2.13±0.36 |
| Lymphocyte (%) | 59.16±4.51 | 57.68±5.64 | 40.63±4.86 | 56.40±4.30 | 47.93±9.66 | 40.98±7.19 |
| Monocyte (%) | 6.22±1.97 | 8.38±2.54 | 12.04±1.90 | 8.00±2.81 | 9.18±3.09 | 9.33±2.21 |
| Neutrophil (%) | 28.43±4.12 | 26.38±6.12 | 43.16±6.40 | 28.99±4.27 | 32.20±8.40 | 39.87±5.95 |
| RBC (10^12^ L^−1^) | 5.74±0.48 | 3.47±0.95 | 6.20±0.36 | 5.23±0.94 | 4.88±0.46 | 6.03±0.43 |
| HGB (g L^−1^) | 144.40±10.52 | 89.25±24.41 | 153.10±7.03 | 133.30±17.37 | 148.60±10.53 | 148.2±9.94 |
| HCT (%) | 25.26±1.51 | 15.33±4.19 | 26.35±1.18 | 22.04±3.79 | 25.08±1.58 | 24.81±1.49 |
| MCV (fL) | 44.23±1.06 | 43.88±0.75 | 42.88±0.74 | 42.37±0.51 | 41.81±0.85 | 41.48±0.42 |
| MCH (pg) | 25.25±0.25 | 25.73±0.20 | 25.08±0.14 | 26.55±2.04 | 24.98±0.14 | 24.72±0.18 |
| MCHC (g L^−1^) | 571.50±9.21 | 586.30±10.16 | 579.80±6.99 | 628.30±40.77 | 591.00±5.35 | 592.00±0.696 |
| PLT (10^9^ L^−1^) | 525.50±41.93 | 536.30±96.28 | 555.80±46.49 | 463.80±78.75 | 542.50±42.69 | 718.50±111.20 |
| MPV (fL) | 5.19±0.14 | 5.55±0.19 | 5.73±0.30 | 5.51±0.37 | 5.73±0.32 | 5.65±0.15 |
| PDW (%) | 14.61±0.05 | 14.75±0.24 | 14.65±0.06 | 14.82±0.11 | 14.65±0.12 | 14.83±0.13 |

**Supplementary Table 3** Blood Routine Examination on Days 7

| Parameter | Sham | Sham+JLSV | Sham+JLSV-BMSC | MI | MI+JLSV | MI+JLSV-BMSC |
| --- | --- | --- | --- | --- | --- | --- |
| WBC (10^9^ L^−1^) | 5.38±0.74 | 8.49±0.48 | 5.08±1.12 | 4.97±1.53 | 7.58±1.90 | 8.01±2.22 |
| Lymphocyte (10^9^ L^−1^) | 3.96±0.79 | 5.15±0.85 | 3.43±0.89 | 3.12±1.04 | 5.23±1.60 | 4.91±1.27 |
| Monocyte (10^9^ L^−1^) | 0.22±0.14 | 0.61±0.11 | 0.22±0.12 | 0.31±0.17 | 0.48±0.17 | 0.65±0.31 |
| Neutrophil (10^9^ L^−1^) | 1.02±0.22 | 1.93±0.51 | 1.22±0.22 | 1.19±0.33 | 1.40±0.19 | 2.18±0.45 |
| Lymphocyte (%) | 3.96±0.79 | 5.15±0.85 | 3.43±0.89 | 3.12±1.04 | 5.23±1.60 | 4.91±1.27 |
| Monocyte (%) | 4.10±2.78 | 7.23±1.33 | 4.82±2.56 | 4.95±2.64 | 5.88±0.10 | 7.52±1.95 |
| Neutrophil (%) | 20.10±5.51 | 24.15±7.74 | 24.81±1.79 | 23.77±5.44 | 21.98±4.89 | 23.40±4.79 |
| RBC (10^12^ L^−1^) | 6.86±0.32 | 5.62±0.64 | 5.28±0.84 | 4.61±1.26 | 5.53±0.50 | 6.35±0.31 |
| HGB (g L^−1^) | 170.00±6.75 | 136.50±17.67 | 132.40±20.45 | 114.80±31.72 | 133.80±11.82 | 158.50±6.48 |
| HCT (%) | 27.91±1.13 | 23.73±2.11 | 21.39±3.50 | 19.45±5.19 | 23.30±2.18 | 27.23±0.71 |
| MCV (fL) | 40.53±0.90 | 42.60±1.32 | 40.43±0.49 | 42.39±0.85 | 42.05±0.49 | 43.05±1.26 |
| MCH (pg) | 24.95±0.39 | 24.10±0.54 | 25.07±0.27 | 24.98±0.79 | 24.28±0.7718 | 25.19±0.33 |
| MCHC (g L^−1^) | 609.20±7.90 | 568.80±28.53 | 621.40±9.10 | 586.60±12.65 | 577.50±24.81 | 583.10±13.84 |
| PLT (10^9^ L^−1^) | 683.80±66.00 | 541.30±45.54 | 541.80±89.31 | 525.05±69.26 | 545.00±48.72 | 543.00±125.70 |
| MPV (fL) | 5.54±0.14 | 5.50±0.15 | 5.32±0.12 | 5.48±0.17 | 5.55±0.10 | 5.57±0.25 |
| PDW (%) | 14.77±0.11 | 14.63±0.10 | 14.72±0.19 | 14.85±0.07 | 14.58±0.08 | 14.93±0.18 |

**Supplementary Table 4** Blood Routine Examination on Days 14

| Parameter | Sham | Sham+JLSV | Sham+JLSV-BMSC | MI | MI+JLSV | MI+JLSV-BMSC |
| --- | --- | --- | --- | --- | --- | --- |
| WBC (10^9^ L^−1^) | 5.81±1.15 | 3.37±0.70 | 3.218±0.7458 | 2.91±0.66 | 4.55±0.47 | 3.74±0.97 |
| Lymphocyte (10^9^ L^−1^) | 2.68±0.51 | 2.08±0.38 | 1.71±0.37 | 1.99±0.68 | 2.21±0.44 | 2.02±0.58 |
| Monocyte (10^9^ L^−1^) | 0.54±0.12 | 0.23±0.11 | 0.28±0.12 | 0.15±0.05 | 0.48±0.08 | 0.55±0.15 |
| Neutrophil (10^9^ L^−1^) | 2.11±0.54 | 0.94±0.22 | 1.06±0.28 | 0.66±0.26 | 1.54±0.15 | 1.01±0.24 |
| Lymphocyte (%) | 46.76±4.39 | 62.65±5.92 | 55.79±6.41 | 64.80±9.77 | 47.68±6.83 | 53.88±4.49 |
| Monocyte (%) | 9.47±1.12 | 6.21±1.63 | 7.80±2.19 | 7.28±3.18 | 10.49±1.54 | 14.10±1.91 |
| Neutrophil (%) | 35.75±4.64 | 27.79±5.58 | 31.55±6.66 | 23.90±8.78 | 34.80±4.85 | 27.18±2.37 |
| RBC (10^12^ L^−1^) | 6.60±0.34 | 6.36±0.31 | 6.26±0.304 | 5.33±0.54 | 6.05±0.39 | 5.34±0.27 |
| HGB (g L^−1^) | 162.25±7.97 | 155.94±7.17 | 152.39±7.17 | 124.75±15.75 | 148.05±9.51 | 127.50±7.44 |
| HCT (%) | 26.53±1.46 | 26.71±1.19 | 25.30±1.31 | 21.95±2.12 | 24.63±1.65 | 21.88±1.27 |
| MCV (fL) | 40.17±0.38 | 42.01±0.46 | 40.53±0.32 | 41.25±0.39 | 40.77±0.17 | 40.98±0.60 |
| MCH (pg) | 24.67±0.18 | 24.68±0.32 | 24.43±0.17 | 23.15±0.79 | 24.58±0.10 | 23.90±1.06 |
| MCHC (g L^−1^) | 613.68±10.38 | 585.45±4.73 | 604.13±3.62 | 562.00±22.51 | 603.88±3.63 | 584.00±22.79 |
| PLT (10^9^ L^−1^) | 784.50±123.03 | 625.00±115.98 | 853.25±98.11 | 887.25±123.39 | 777.25±64.52 | 743.00±102.82 |
| MPV (fL) | 5.39±0.19 | 5.10±0.11 | 5.03±0.05 | 5.43±0.34 | 5.01±0.08 | 5.28±0.18 |
| PDW (%) | 14.73±0.13 | 14.70±0.11 | 14.55±0.10 | 14.60±0.108 | 14.58±0.18 | 14.60±0.04 |

**Supplementary Table 5** Blood Routine Examination on Days 28

| Parameter | Sham | Sham+JLSV | Sham+JLSV-BMSC | MI | MI+JLSV | MI+JLSV-BMSC |
| --- | --- | --- | --- | --- | --- | --- |
| WBC (10^9^ L^−1^) | 3.95±0.70 | 3.31±0.57 | 3.60±0.65 | 5.97±2.56 | 6.75±1.06 | 4.40±2.21 |
| Lymphocyte (10^9^ L^−1^) | 2.19±0.54 | 1.75±0.30 | 1.93±0.36 | 4.46±2.22 | 4.87±0.63 | 3.06±1.35 |
| Monocyte (10^9^ L^−1^) | 0.37±0.09 | 0.36±0.08 | 0.39±0.09 | 0.50±0.19 | 0.60±0.23 | 0.20±0.10 |
| Neutrophil (10^9^ L^−1^) | 1.15±0.22 | 1.02±0.19 | 1.10±0.23 | 0.73±0.23 | 0.90±0.38 | 0.90±0.72 |
| Lymphocyte (%) | 53.97±6.29 | 53.83±4.20 | 53.80±3.40 | 71.08±5.77 | 74.60±7.44 | 74.80±7.73 |
| Monocyte (%) | 9.48±1.60 | 10.43±1.11 | 10.48±0.88 | 8.48±1.10 | 8.45±2.78 | 4.50±0.387 |
| Neutrophil (%) | 29.90±6.29 | 30.77±3.04 | 30.53±3.06 | 15.25±4.60 | 11.68±3.97 | 14.23±5.80 |
| RBC (10^12^ L^−1^) | 5.52±0.87 | 5.91±0.38 | 5.50±0.77 | 5.11±0.80 | 5.29±0.56 | 4.67±0.50 |
| HGB (g L^−1^) | 133.74±21.36 | 146.26±8.20 | 129.78±21.04 | 116.65±20.72 | 111.25±10.27 | 114.00±6.50 |
| HCT (%) | 22.08±3.50 | 24.75±1.42 | 22.06±3.37 | 21.58±3.24 | 23.05±2.34 | 23.75±2.72 |
| MCV (fL) | 40.13±0.81 | 41.80±0.37 | 40.04±0.89 | 42.15±0.77 | 43.62±0.69 | 42.18±1.91 |
| MCH (pg) | 24.44±0.46 | 24.85±0.45 | 23.47±0.82 | 22.69±0.67 | 23.40±1.03 | 22.50±0.95 |
| MCHC (g L^−1^) | 556.10±46.77 | 545.05±38.73 | 583.78±6.87 | 539.25±24.27 | 520.00±23.32 | 531.75±8.61 |
| PLT (10^9^ L^−1^) | 754.50±182.36 | 722.25±120.05 | 673.75±158.47 | 636.00±129.70 | 831.75±84.95 | 772.75±140.34 |
| MPV (fL) | 5.07±0.13 | 5.52±0.19 | 5.08±0.11 | 5.35±0.21 | 5.80±0.15 | 5.40±0.34 |
| PDW (%) | 14.68±0.16 | 14.69±0.03 | 14.74±0.19 | 14.91±0.08 | 14.85±0.09 | 14.68±0.18 |

**Supplementary Table 6** Serum Biochemical Parameters on Day 1

| Parameter | Sham | Sham+JLSV | Sham+JLSV-BMSC | MI | MI+JLSV | MI+JLSV-BMSC |
| --- | --- | --- | --- | --- | --- | --- |
| ALT (U L^−1^) | 58.34±8.471 | 36.21±3.607 | 39.27±12.59 | 63.16±26.25 | 88.23±11.58 | 73.20±11.41 |
| AST (U L^−1^) | 102.70±5.616 | 144.30±12.43 | 104.10±20.91 | 135.60±22.01 | 116.70±5.930 | 155.40±15.60 |
| ALB (g L^−1^) | 13.01±0.37 | 11.45±0.26 | 10.31±0.59 | 10.52±0.57 | 11.10±0.17 | 14.63±5.07 |
| ALP (U L^−1^) | 45.05±7.23 | 54.05±6.58 | 57.93±15.52 | 37.51±6.78 | 34.80±5.01 | 61.83±3.64 |
| TBIL(μmol L^−1^) | 14.57±0.82 | 12.54±1.72 | 13.24±2.05 | 10.65±1.26 | 9.350±0.76 | 8.639±3.45 |
| DBIL (μmol L^−1^) | 3.79±1.55 | 4.58±1.40 | 2.63±1.01 | 4.64±1.11 | 6.46±0.47 | 3.54±0.75 |
| GGT (U L^−1^) | 0.76±0.03 | 0.77±0.03 | 0.74±0.05 | 0.80±0.02 | 0.84±0.01 | 0.65±0.07 |
| UREA (mg dL^−1^) | 13.31±1.30 | 19.98±2.39 | 19.56±2.92 | 36.38±7.13* | 17.42±1.44 | 17.61±2.33 |
| CREA (mg dL^−1^) | 31.88±2.23 | 37.05±2.02 | 37.30±2.39 | 28.55±5.55 | 30.84±8.28 | 24.76±2.29 |

*p<0.05 indicates a significant difference compared with the Sham group

**Supplementary Table 7** Serum Biochemical Parameters on Day 3

| Parameter | Sham | Sham+JLSV | Sham+JLSV-BMSC | MI | MI+JLSV | MI+JLSV-BMSC |
| --- | --- | --- | --- | --- | --- | --- |
| ALT (U L^−1^) | 32.54±2.82 | 37.43±7.47 | 33.93±9.73 | 28.34±6.44 | 15.21±4.97 | 33.92±10.64 |
| AST (U L^−1^) | 94.22±16.12 | 101.00±9.40 | 100.50±4.35 | 102.20±20.69 | 126.70±17.87 | 77.37±38.70 |
| ALB (g L^−1^) | 15.84±0.33 | 14.91±1.04 | 14.39±0.81 | 12.62±2.42 | 10.28±1.92 | 15.33±0.58 |
| ALP (U L^−1^) | 31.26±5.04 | 32.11±7.75 | 23.29±6.79 | 25.82±5.00 | 26.96±1.56 | 39.46±16.44 |
| TBIL(μmol L^−1^) | 22.11±6.96 | 9.96±1.90 | 7.06±2.03 | 16.94±3.93 | 11.12±1.14 | 14.04±3.86 |
| DBIL (μmol L^−1^) | 3.71±0.19 | 8.02±2.82 | 3.50±0.70 | 5.30±0.58 | 3.75±0.89 | 6.08±1.90 |
| GGT (U L^−1^) | 0.84±0.01 | 0.73±0.06 | 0.80±0.02 | 0.79±0.04 | 0.75±0.10 | 0.74±0.04 |
| UREA (mg dL^−1^) | 19.07±12.22 | 17.77±3.54 | 22.86±1.08 | 27.56±3.94 | 23.58±1.01 | 22.10±2.55 |
| CREA (mg dL^−1^) | 38.35±1.39 | 41.03±2.92 | 44.11±8.50 | 35.13±2.20 | 32.14±5.74 | 45.00±2.33 |

**Supplementary Table 8** Serum Biochemical Parameters on Day 7

| Parameter | Sham | Sham+JLSV | Sham+JLSV-BMSC | MI | MI+JLSV | MI+JLSV-BMSC |
| --- | --- | --- | --- | --- | --- | --- |
| ALT (U L^−1^) | 39.01±7.41 | 65.93±20.22 | 78.07±11.86 | 52.20±12.92 | 34.90±8.06 | 33.20±13.42 |
| AST (U L^−1^) | 102.50±7.67 | 121.60±31.75 | 121.50±19.71 | 119.40±31.25 | 144.50±10.72 | 106.70±16.89 |
| ALB (g L^−1^) | 11.72±0.81 | 11.44±2.40 | 9.23±0.58 | 10.13±0.33 | 10.76±0.60 | 12.17±1.54 |
| ALP (U L^−1^) | 33.85±5.13 | 39.72±7.82 | 20.17±6.10 | 41.07±3.39 | 24.70±5.77 | 47.68±8.82 |
| TBIL(μmol L^−1^) | 6.29±2.08 | 5.69±0.47 | 4.43±0.47 | 7.44±0.63 | 6.82±0.95 | 7.44±0.63 |
| DBIL (μmol L^−1^) | 3.04±1.28 | 1.63±0.45 | 5.83±1.51 | 1.99±0.43 | 1.88±0.68 | 2.63±0.55 |
| GGT (U L^−1^) | 1.42±0.57 | 0.94±0.36 | 1.80±0.44 | 1.25±0.32 | 0.93±0.38 | 3.29±1.02 |
| UREA (mg dL^−1^) | 24.50±1.78 | 23.06±1.37 | 21.67±1.65 | 26.01±1.93 | 24.21±2.03 | 25.22±1.25 |
| CREA (mg dL^−1^) | 17.47±2.62 | 23.62±5.58 | 22.92±2.15 | 21.13±1.73 | 21.02±2.77 | 21.15±2.42 |

**Supplementary Table 9** Serum Biochemical Parameters on Day 14.

| Parameter | Sham | Sham+JLSV | Sham+JLSV-BMSC | MI | MI+JLSV | MI+JLSV-BMSC |
| --- | --- | --- | --- | --- | --- | --- |
| ALT (U L^−1^) | 77.01±23.97 | 44.00±12.09 | 63.17±15.42 | 86.76±27.56 | 77.06±14.70 | 84.58±23.34 |
| AST (U L^−1^) | 158.00±33.70 | 175.80±22.78 | 191.90±25.27 | 176.90±38.96 | 144.80±10.85 | 153.00±16.86 |
| ALB (g L^−1^) | 15.43±0.74 | 16.08±0.54 | 16.61±0.42 | 14.97±0.93 | 17.17±0.60 | 16.07±0.51 |
| ALP (U L^−1^) | 92.17±13.42 | 136.40±8.93 | 105.00±14.78 | 110.60±8.22 | 126.10±14.02 | 91.95±15.11 |
| TBIL(μmol L^−1^) | 9.99±1.33 | 15.14±1.42 | 16.38±1.51 | 11.82±1.14 | 7.81±0.69 | 10.26±2.22 |
| DBIL (μmol L^−1^) | 3.78±0.98 | 5.44±1.40 | 7.10±1.30 | 5.92±0.86 | 4.77±0.56 | 7.28±1.80 |
| GGT (U L^−1^) | 2.447±0.77 | 4.620±1.06 | 2.905±1.46 | 4.587±1.52 | 5.324±0.52 | 4.822±0.876 |
| UREA (mg dL^−1^) | 3.78±0.98 | 5.44±1.40 | 7.10±1.30 | 5.92±0.86 | 4.77±0.56 | 7.28±1.81 |
| CREA (mg dL^−1^) | 22.84±1.94 | 23.00±1.66 | 22.94±1.19 | 26.15±2.68 | 25.25±2.29 | 20.89±3.79 |

**Supplementary Table 10** Serum Biochemical Parameters on Day 28.

| Parameter | Sham | Sham+JLSV | Sham+JLSV-BMSC | MI | MI+JLSV | MI+JLSV-BMSC |
| --- | --- | --- | --- | --- | --- | --- |
| ALT (U L^−1^) | 30.29±3.48 | 43.06±14.20 | 28.10±6.61 | 29.64±4.16 | 32.82±7.34 | 32.39±2.87 |
| AST (U L^−1^) | 116.90±14.58 | 174.60±20.77 | 117.00±13.84 | 133.60±14.36 | 127.10±15.42 | 152.50±26.89 |
| ALB (g L^−1^) | 26.31±3.38 | 29.21±1.40 | 28.80±4.41 | 25.95±3.22 | 25.52±3.35 | 29.83±1.76 |
| ALP (U L^−1^) | 99.67±17.85 | 91.46±9.20 | 81.91±5.74 | 103.70±19.53 | 64.61±11.15 | 110.1±15.60 |
| TBIL(μmol L^−1^) | 16.48±2.24 | 21.86±2.61 | 15.43±1.38 | 21.76±5.05 | 15.86±2.84 | 15.90±15.90 |
| DBIL (μmol L^−1^) | 6.460±0.53 | 8.60±1.29 | 6.27±1.00 | 7.57±2.31 | 5.95±0.82 | 6.60±0.84 |
| GGT (U L^−1^) | 0.36±0.16 | 0.63±0.13 | 0.66±0.11 | 0.61±0.10 | 0.62±0.07 | 0.64±0.11 |
| UREA (mg dL^−1^) | 21.88±2.56 | 21.76±1.37 | 28.23±2.32 | 28.59±3.42 | 25.23±1.70 | 24.67±0.75 |
| CREA (mg dL^−1^) | 26.44±0.91 | 24.93±2.79 | 18.05±2.43 | 26.88±2.01 | 27.35±1.04 | 17.38±3.93 |

**Supplementary Table 11** Inflammatory factors from Day 1 to Day 28.

| Parameter | Sham | Sham+JLSV | Sham+JLSV-BMSC | MI | MI+JLSV | MI+JLSV-BMSC |
| --- | --- | --- | --- | --- | --- | --- |
| **Day 1** |  |  |  |  |  |  |
| CRP  (μg mL^−1^) | 1.25±0.13 | 1.27±0.20 | 1.78±0.29 | 1.07±0.09 | 1.21±0.35 | 1.22±0.23 |
| TNF-α  (pg mL^−1^) | 4.58±0.78* | 5.78±2.37* | 9.00±2.56 * | 45.37±12.10 | 28.56±16.47 | 3.49±1.24** |
| IL-6  (pg mL^−1^) | 26.11±13.07* | 22.17±8.34** | 25.57±11.30 * | 125.96±42.71 | 44.41±18.59 | 12.67±5.26** |
| **Day 3** |  |  |  |  |  |  |
| CRP  (μg mL^−1^) | 1.36±0.48 | 1.42±0.46 | 1.17±0.17 | 1.15±0.14 | 1.58±0.27 | 1.35±0.32 |
| TNF-α  (pg mL^−1^) | 9.02±4.08* | 8.67±6.97 * | 6.58±1.00 * | 46.29±14.95 | 18.14±6.74 | 5.92±2.41* |
| IL-6  (pg mL^−1^) | 33.60±19.80 * | 19.03±11.12 ** | 9.82±4.98 ** | 116.55±33.03 | 51.71±14.78 | 9.17±4.42 ** |
| **Day 7** |  |  |  |  |  |  |
| CRP  (μg mL^−1^) | 2.04±0.19 | 2.08±0.26 | 2.19±0.16 | 1.79±0.09 | 2.6±0.49 | 1.86±0.39 |
| TNF-α  (pg mL^−1^) | 5.33±1.00 * | 3.13±0.91 * | 3.06±0.70 * | 71.97±12.31 | 50.44±29.19 | 4.11±1.41 * |
| IL-6  (pg mL^−1^) | 10.91±4.36 ** | 8.14±6.58 ** | 9.81±4.30 ** | 40.84±4.47 | 23.32±4.25 | 12.06±2.984* |
| **Day 14** |  |  |  |  |  |  |
| CRP  (μg mL^−1^) | 3.59±0.2 | 3.98±0.08 | 3.18±0.31 | 3.61±0.29 | 4.27±0.41 | 3.51±0.33 |
| TNF-α  (pg mL^−1^) | 7.53±1.11 *** | 11.40±5.23 ** | 8.15±2.02 | 43.45±5.87 | 20.11±4.83 * | 6.38±4.44*** |
| IL-6  (pg mL^−1^) | 2.28±0.11 ** | 2.77±1.55 ** | 1.85±0.32 *** | 10.85±1.07 | 5.90±1.62 | 4.35±1.175 * |
| **Day 28** |  |  |  |  |  |  |
| CRP  (μg mL^−1^) | 1.73±0.18 | 1.84±0.21 | 1.77±0.12 | 2.23±0.12 | 2±0.2 | 1.87±0.31 |
| TNF-α  (pg mL^−1^) | 3.62±0.89* | 5.08±1.31* | 5.41±0.45* | 15.55±4.33 | 7.4±2.01 | 3.52±1.31** |
| IL-6  (pg mL^−1^) | 3.81±1.71*** | 5.24±2.75*** | 2.08±0.42*** | 18.19±1.01 | 12.67±1.13 | 9.25±0.93* |

*p<0.05 **p<0.01 ***p<0.001 indicates a significant difference compared with the MI group.
